# Supplementary material for: A bacterial network of T3SS effectors counteracts host pro-inflammatory responses and cell death to promote infection
Source: EMBO J. 2025 Mar 24;44(9):2424–45. doi: 10.1038/s44318-025-00412-5 (PMC12048508; doi:10.1038/s44318-025-00412-5)
Supplement: Supplementary file 1 — Appendix [file 44318_2025_412_MOESM1_ESM.pdf]

Appendix for

A bacterial network of T3SS effectors counteracts host pro-inflammatory responses and cell death to promote infection

Hui Wen Yeap[1,2], Ghin Ray Goh[1,2], Safwah Nasuha Rosli[1,2], Hai Shin Pung[1,2], Cristina Giogha[3,4], Vik Ven Eng[3,5], Jaclyn S Pearson[3,5,6], Elizabeth L Hartland[3,4,5], Kaiwen W Chen[1,2,#]

Appendix Figure S1……………………………………………………………… Page 2
Appendix Figure S2……………………………………………………………… Page 2
Appendix Figure S3...…………………………………………………………… Page 3
Appendix Table S1……………………………………………………………...Page 4

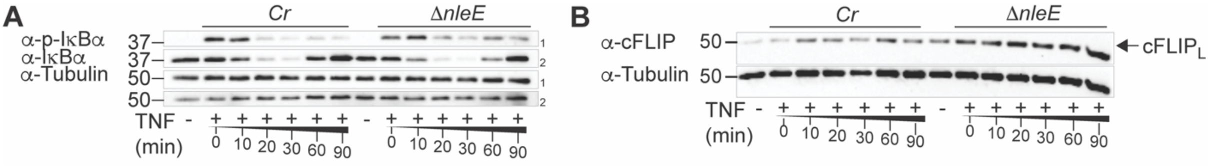

Appendix Figure S1. NleE suppresses TNF-induced NF-κB signalling and expression of the anti-apoptotic protein FLIP in colonic epithelial cells. (A-B) CMT-93 cells were infected with log phase *C. rodentium* (*Cr*) or Δ*nleE* for 1 hour and stimulated with TNF (100 ng/ml) for the indicated time points. Cell lysates were analysed by immunoblot.

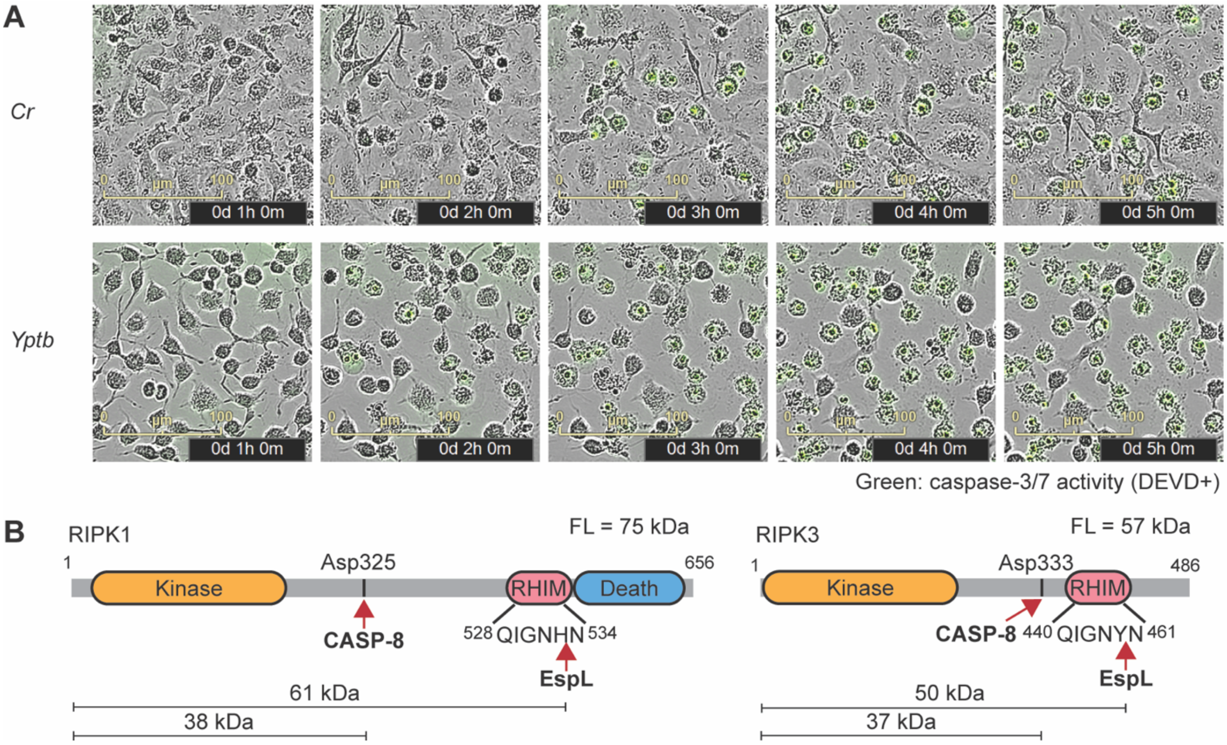

Appendix Figure S2. *C. rodentium* triggers slower apoptotic kinetics compared to *Y. pseudotuberculosis*. (A) IncuCyte images of *C. rodentium* (*Cr*) or *Y. pseudotuberculosis* (*Yptb*)-infected unprimed macrophages over time in the presence of a fluorogenic caspase-3/7 activity dye (DEVD-positive). **(B)** Schematics showing mouse RIPK1 and RIPK3 protein domains, caspase-8 and EspL cleavage sites and the predicted molecular weight for each cleavage product.

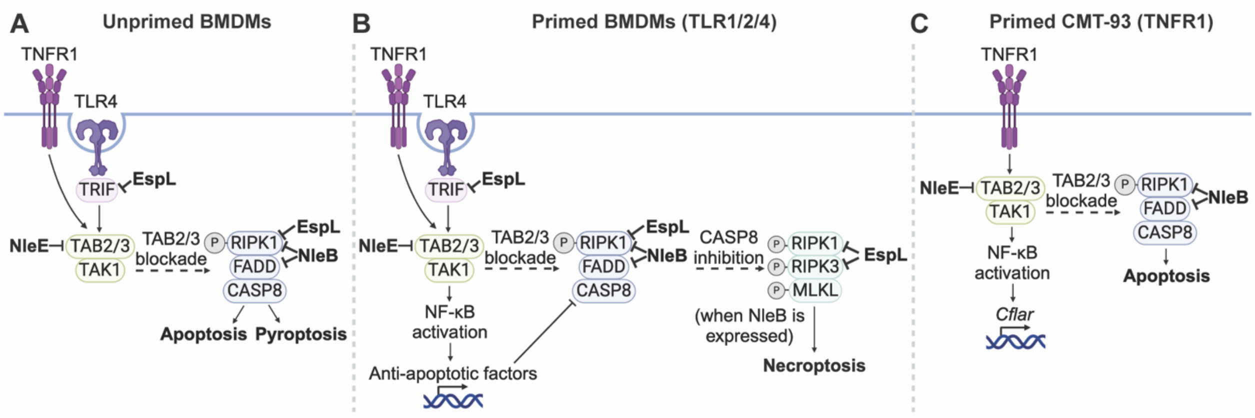

Appendix Figure S3. Summary of *C. rodentium*-induced cell death in macrophages and colonic epithelial cells. (A) Upon macrophage infection, *C. rodentium* injects NleE to target TAB2/3 and subvert TLR4 and TNFR1-induced NF-κB activation. Macrophages respond by triggering RIPK1 kinase-dependent caspase-8 activation, which is subverted by a second effector, NleB. (B) Under conditions where macrophages express high anti-apoptotic proteins, such as upon TLR1/2 or TLR4 priming, NleB blockade of caspase-8 sensitises macrophages to RIPK3-dependent necroptosis, and *C. rodentium* uses a third effector, EspL to cleave and inactivate RIPK1 and RIPK3 to subvert necroptosis. In the absence of NleB, EspL can cleave RHIM-containing proteins to further subvert apoptosis. (C) Injection of NleE in CMT-93 cells to suppress TNF-induced TAB2/3 recruitment and downstream NF-κB activation likewise promotes RIPK1 kinase-dependent caspase-8 activation in colonic epithelial cells that are suppressed by injection of a second effector, NleB. Unlike macrophages, CMT-93 cells do not undergo necroptosis in the absence of EspL, even under conditions where RIPK3 is overexpressed.

Appendix Table S1

Primer	Sequence (5'-> 3')
Ripk3$_F$	aattatgcggccgcgccatgtcttctgtcaagttatggc
Ripk3$_R$	ctagtctagatacttgtggaagggctgccagcccctac
pEF6-F	tcaagcctcagacagtggttc
pEF6-R	ttaggaaaggacagtgggagtggc
CRespL-5'HR-F	aaagacgatgacgataaaatagagagttgtgcacaaaaca
CRespL-5'HR-R	aagcagctccagcctacacaatgtcctataagatgtccttgac
CRespL-3'HR-F	ctaaggaggatattcatatgtataacaattaaaggaagggcac
CRespL-3'HR-R	agggccctctagatgcatgcatgatctgcgcagcattcag
CRespL-HR-Cm-F	aaggacatcttataggacattgtgtaggctggagctgctt
CRespL-HR-Cm-R	cccttcctttaattgttatacatatgaatatcctccttagttcctattc
pcDNA-F	tctggctaactagagaaccc
pcDNA-R	tagaaggcacagtcgagg
pcDNA-Cm-F	atacgtaattccggatgagc
EspLHR-F	agagagttgtgcacaaaacagtc
EspLHR-R	atgatctgcgcagcattcag
Cr-S12-F	acgttttaattaatcagatccgcgatcgttgtatatttct
Cr-S12-R1	acatttaatggagataacattaaatagctcctggttttagct
Cr-S12-R2	gtattatttactactggcattaaatagctcctggttttagct
S12-Cr-nleB-F	ctaaaaccaggagctatttaatgttatctccattaaatgttcttca
S12-Cr-nleB-R	gtggcgcgcctcctaggtgcttaccatgaactgttggtataca
S12-Cr-espL-F	ctaaaaccaggagctatttaatgccagtagtaaataatactgaatac
S12-Cr-espL-R	gtggcgcgcctcctaggtgcttaattgttatattcattagagtaataccc
pGRG36-F	atcaaagctgccgacaacac
pGRG36-R	cggagtagcatagggtttgc
Check-Cr-nleB-F2	ccaaagagcatccgtatttgagc
Check-Cr-espL-F2	tgaaccgtgtctgaaatgttatgg
Check-Tn7-F2	atccaacatcgaagaggtac
Check-Tn7-R	tggcttataatctgatacgg
