# Supplementary figures and images for: A bacterial network of T3SS effectors counteracts host pro-inflammatory responses and cell death to promote infection (part 1 of 2)
Source: EMBO J. 2025 Mar 24;44(9):2424–45. doi: 10.1038/s44318-025-00412-5 (PMC12048508; doi:10.1038/s44318-025-00412-5)

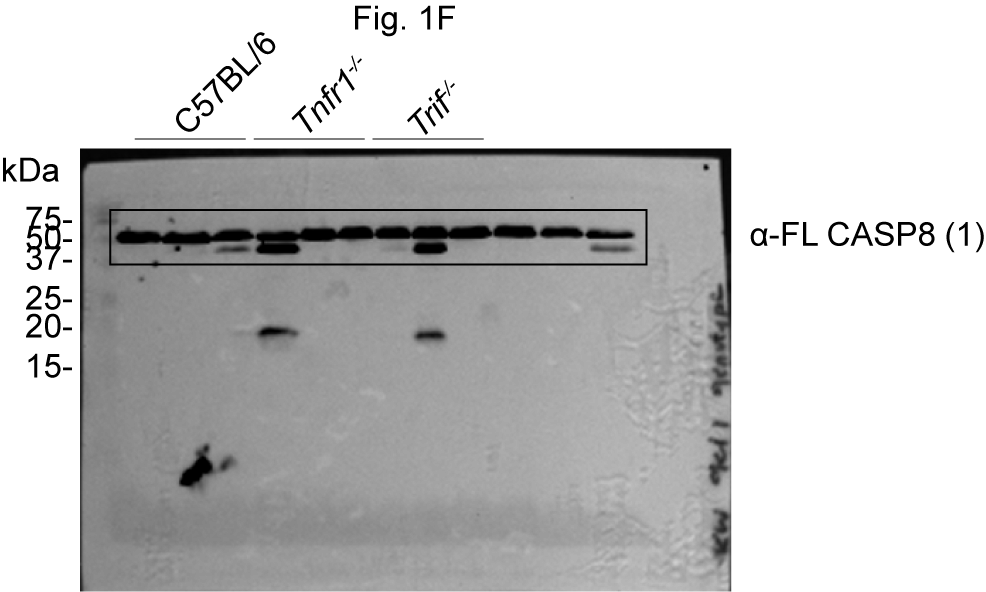

Supplement: Supplementary file 3 — Source data Fig. 1 [file 44318_2025_412_MOESM3_ESM.zip › Figure 1/1F/Fig1F FL Casp8.tif]

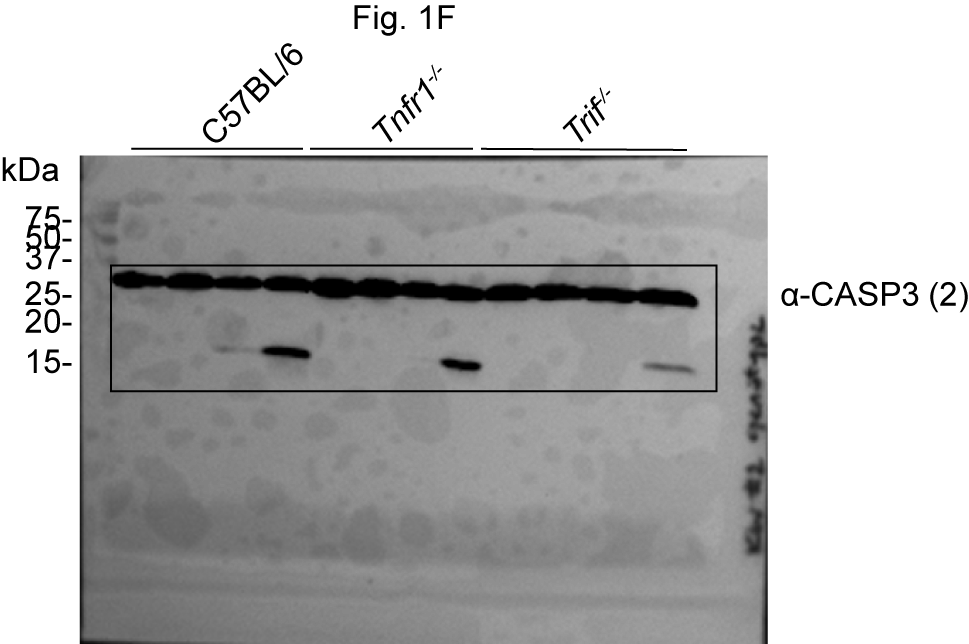

Supplement: Supplementary file 3 — Source data Fig. 1 [file 44318_2025_412_MOESM3_ESM.zip › Figure 1/1F/Fig1F Casp3.tif]

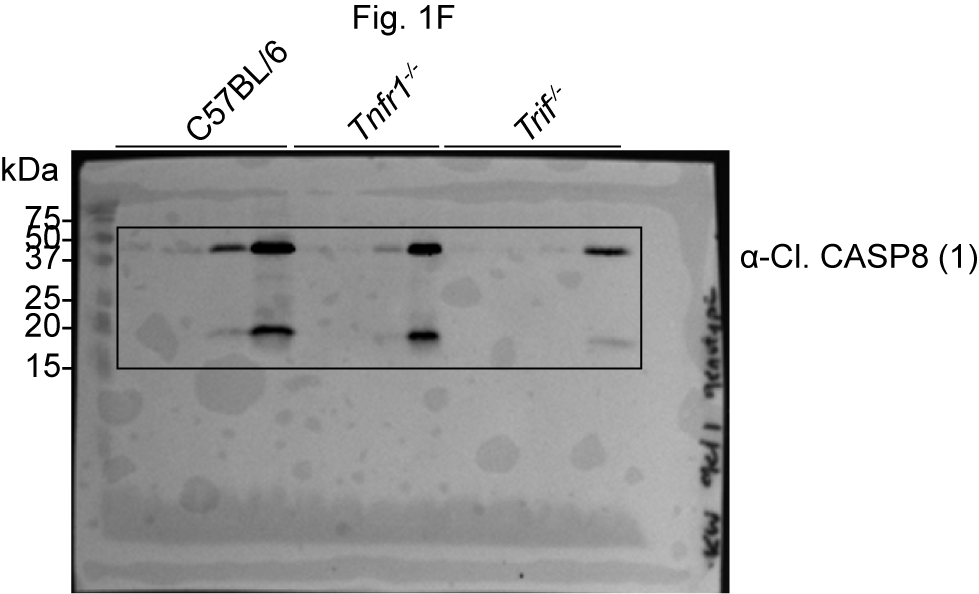

Supplement: Supplementary file 3 — Source data Fig. 1 [file 44318_2025_412_MOESM3_ESM.zip › Figure 1/1F/Fig1F Cl Casp8.tif]

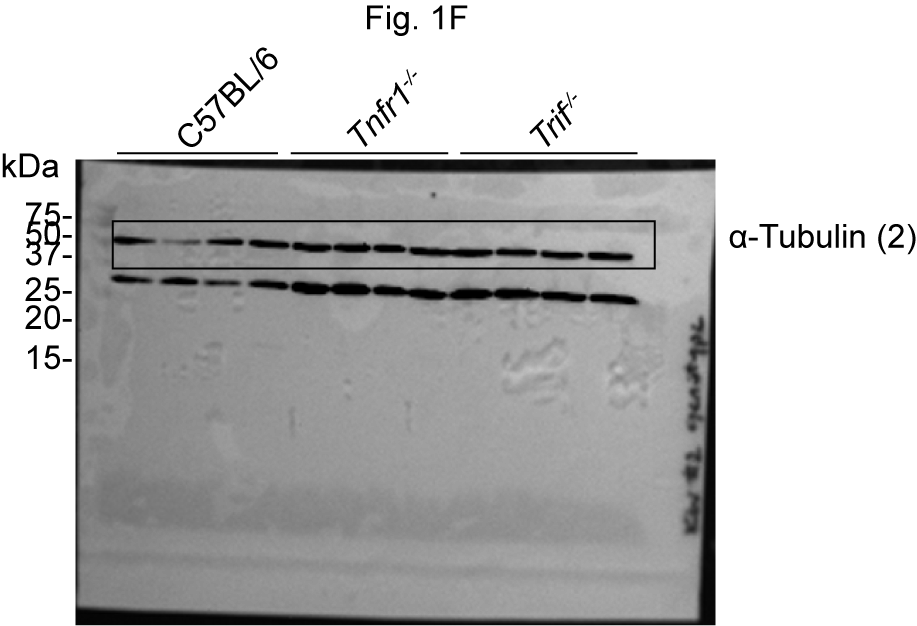

Supplement: Supplementary file 3 — Source data Fig. 1 [file 44318_2025_412_MOESM3_ESM.zip › Figure 1/1F/Fig1F Tubulin 2.tif]

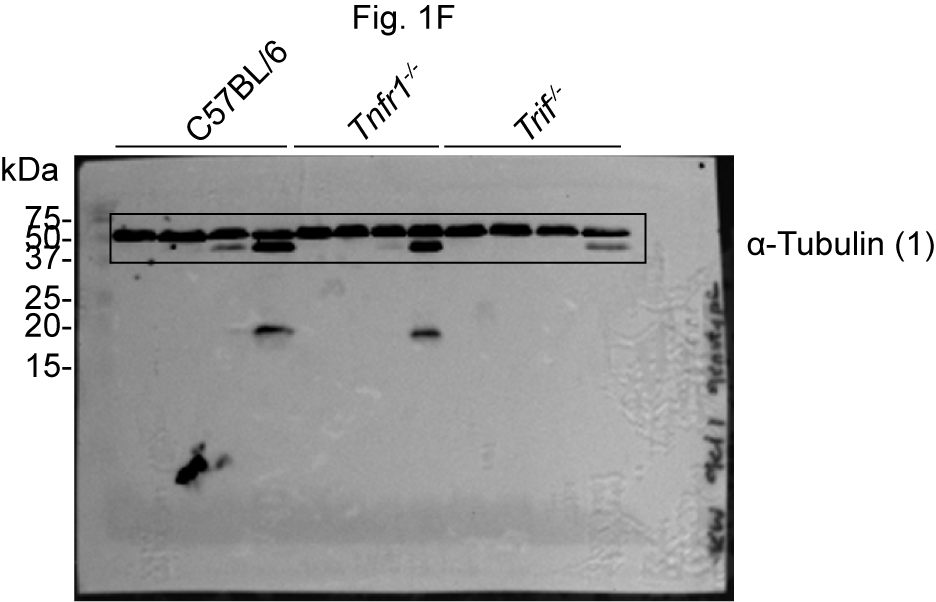

Supplement: Supplementary file 3 — Source data Fig. 1 [file 44318_2025_412_MOESM3_ESM.zip › Figure 1/1F/Fig1F Tubulin 1.tif]

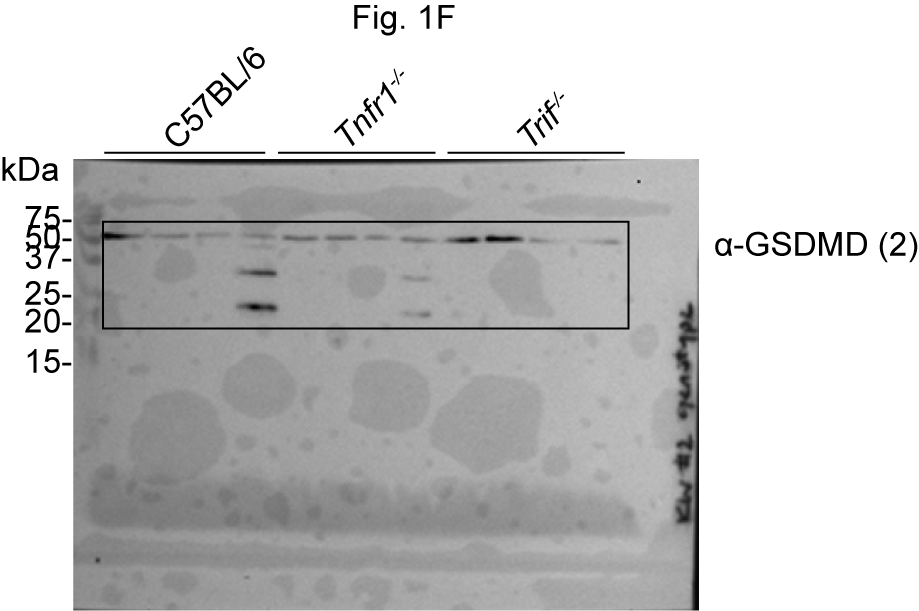

Supplement: Supplementary file 3 — Source data Fig. 1 [file 44318_2025_412_MOESM3_ESM.zip › Figure 1/1F/Fig1F GSDMD.tif]

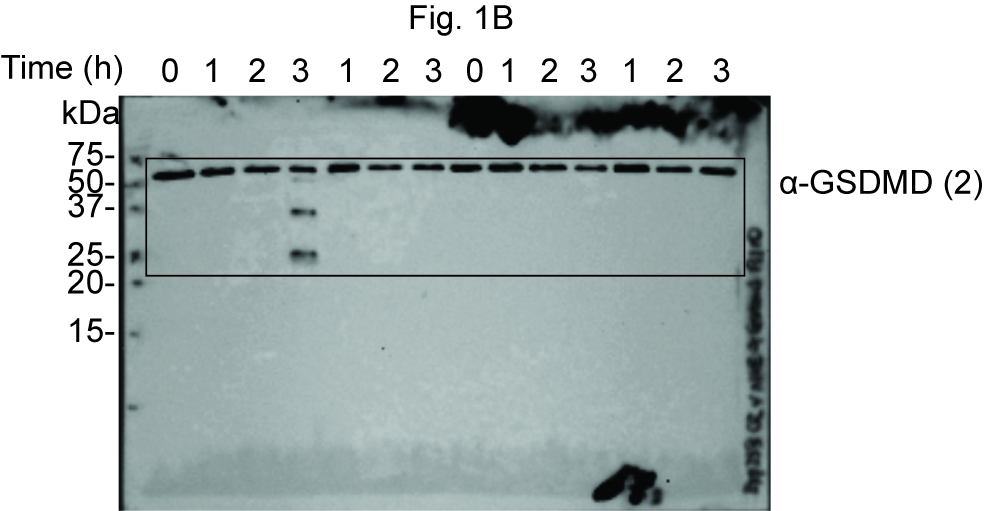

Supplement: Supplementary file 3 — Source data Fig. 1 [file 44318_2025_412_MOESM3_ESM.zip › Figure 1/1B/Fig1B GSDMD.tif]

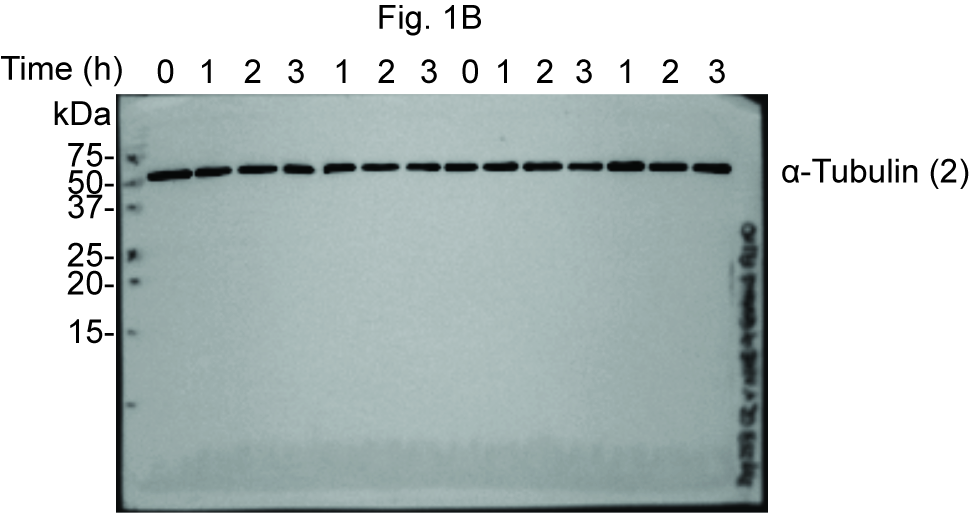

Supplement: Supplementary file 3 — Source data Fig. 1 [file 44318_2025_412_MOESM3_ESM.zip › Figure 1/1B/Fig1B Tubulin 2.tif]

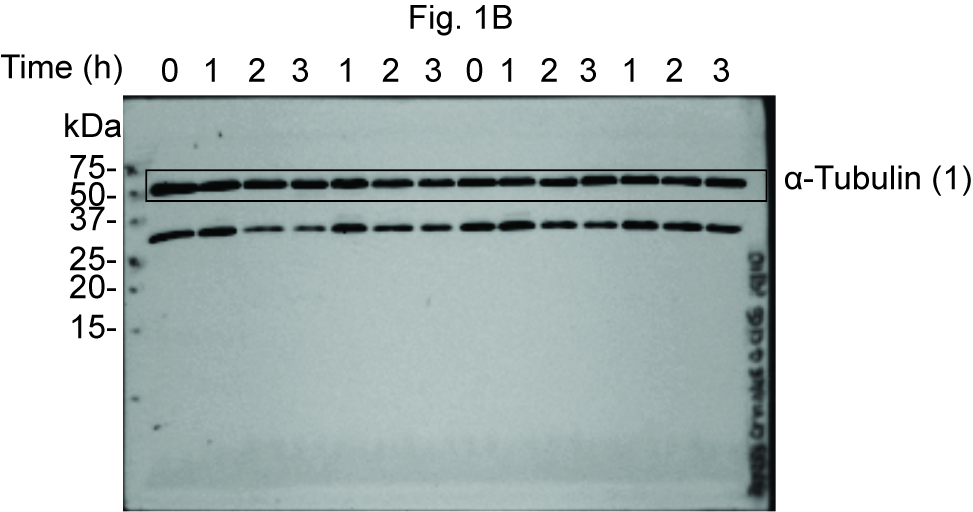

Supplement: Supplementary file 3 — Source data Fig. 1 [file 44318_2025_412_MOESM3_ESM.zip › Figure 1/1B/Fig1B Tubulin 1.tif]

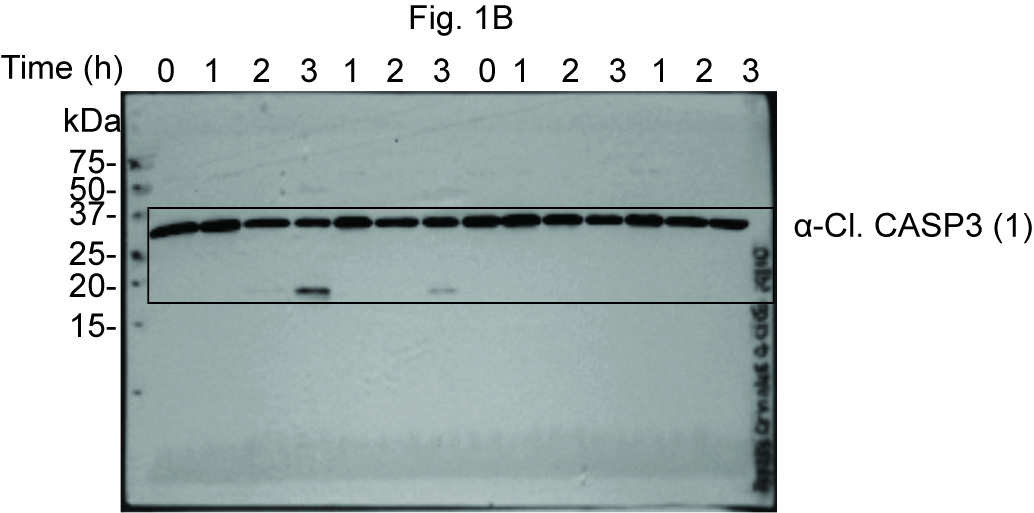

Supplement: Supplementary file 3 — Source data Fig. 1 [file 44318_2025_412_MOESM3_ESM.zip › Figure 1/1B/Fig1B Casp3.tif]

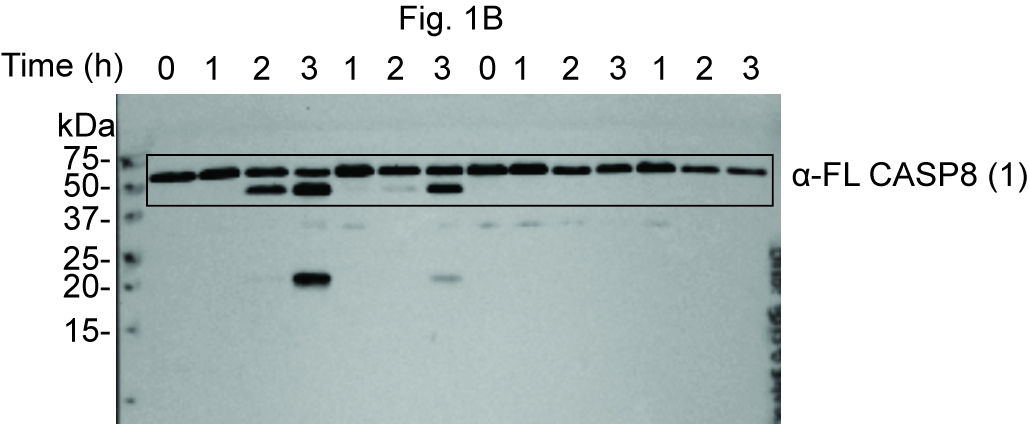

Supplement: Supplementary file 3 — Source data Fig. 1 [file 44318_2025_412_MOESM3_ESM.zip › Figure 1/1B/Fig1B FL Casp8.tif]

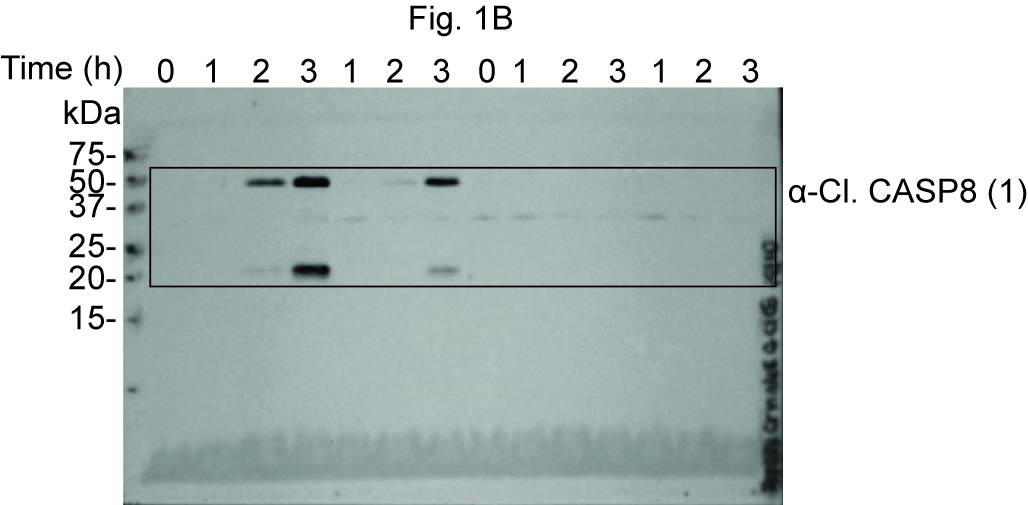

Supplement: Supplementary file 3 — Source data Fig. 1 [file 44318_2025_412_MOESM3_ESM.zip › Figure 1/1B/Fig1B Cl Casp8.tif]

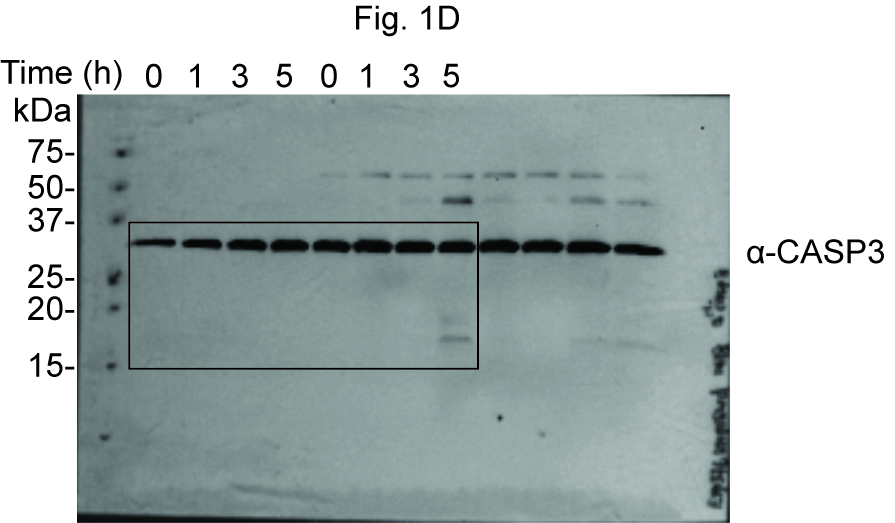

Supplement: Supplementary file 3 — Source data Fig. 1 [file 44318_2025_412_MOESM3_ESM.zip › Figure 1/1D/Fig1D Casp3.tif]

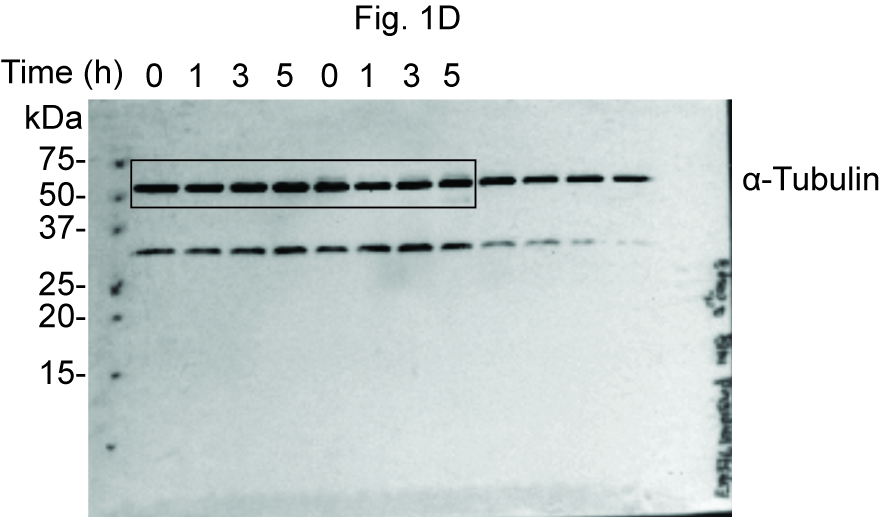

Supplement: Supplementary file 3 — Source data Fig. 1 [file 44318_2025_412_MOESM3_ESM.zip › Figure 1/1D/Fig1D Tubulin.tif]

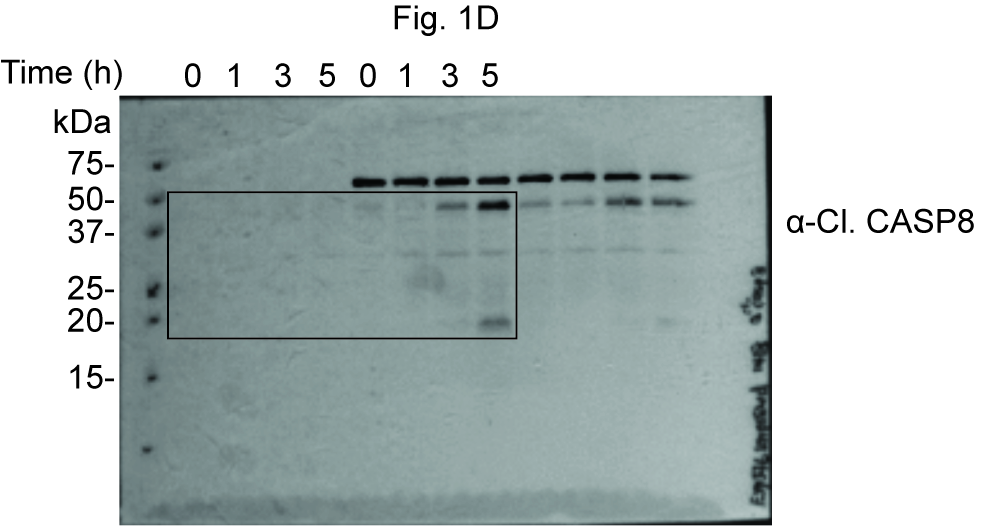

Supplement: Supplementary file 3 — Source data Fig. 1 [file 44318_2025_412_MOESM3_ESM.zip › Figure 1/1D/Fig1D Cl Casp8.tif]

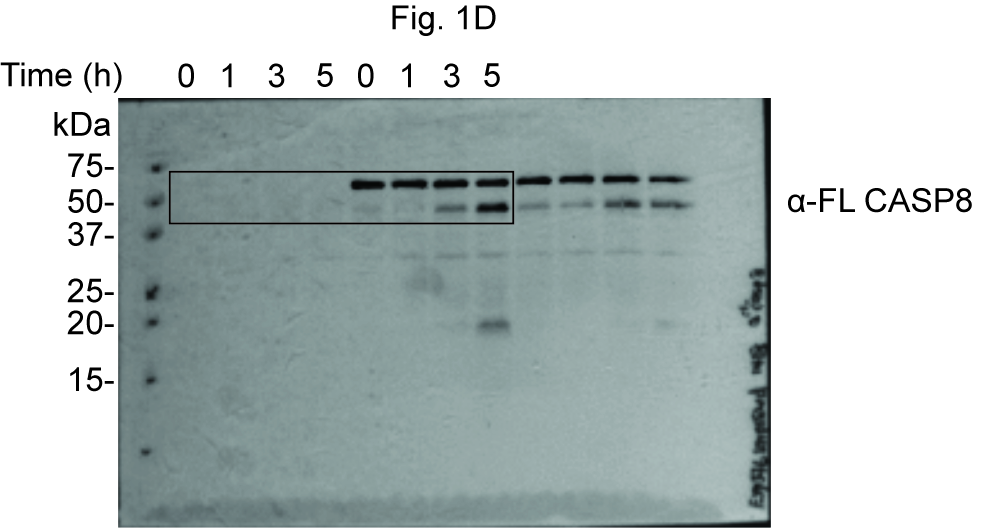

Supplement: Supplementary file 3 — Source data Fig. 1 [file 44318_2025_412_MOESM3_ESM.zip › Figure 1/1D/Fig1D FL Casp8.tif]

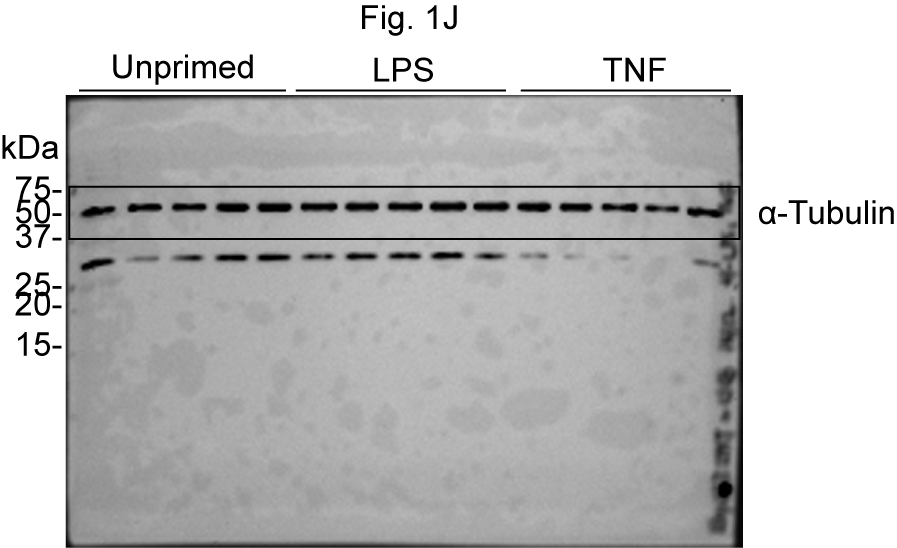

Supplement: Supplementary file 3 — Source data Fig. 1 [file 44318_2025_412_MOESM3_ESM.zip › Figure 1/1J/Fig1J Tubulin.tif]

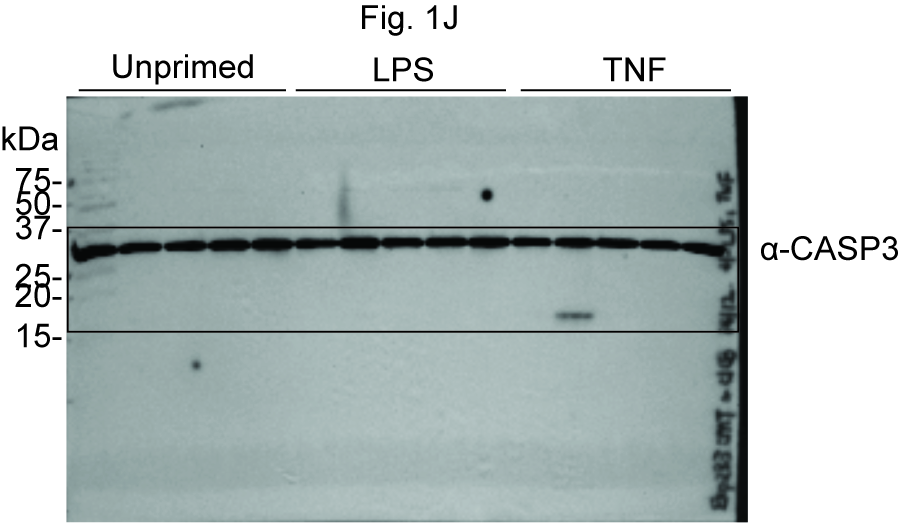

Supplement: Supplementary file 3 — Source data Fig. 1 [file 44318_2025_412_MOESM3_ESM.zip › Figure 1/1J/Fig1J Casp3.tif]

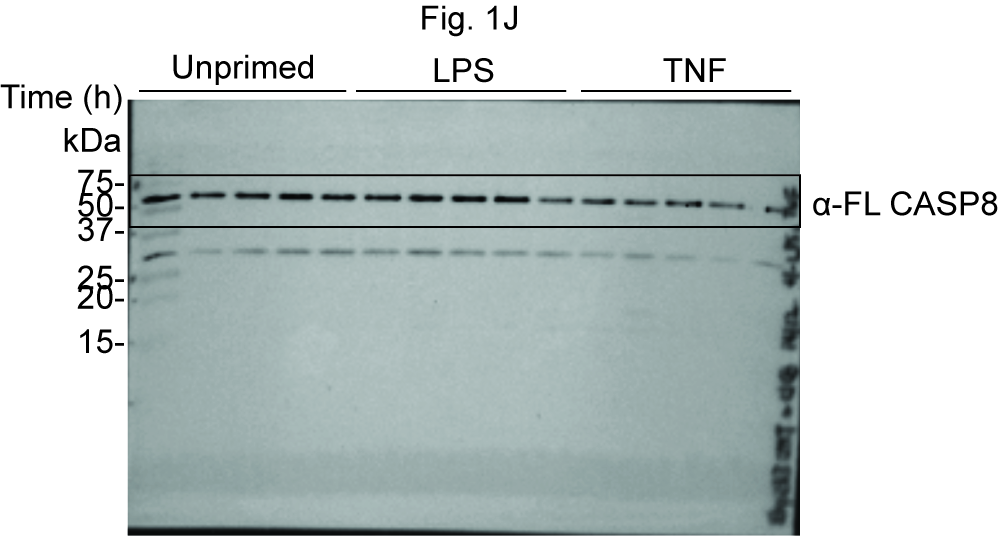

Supplement: Supplementary file 3 — Source data Fig. 1 [file 44318_2025_412_MOESM3_ESM.zip › Figure 1/1J/Fig1J FL Casp8.tif]

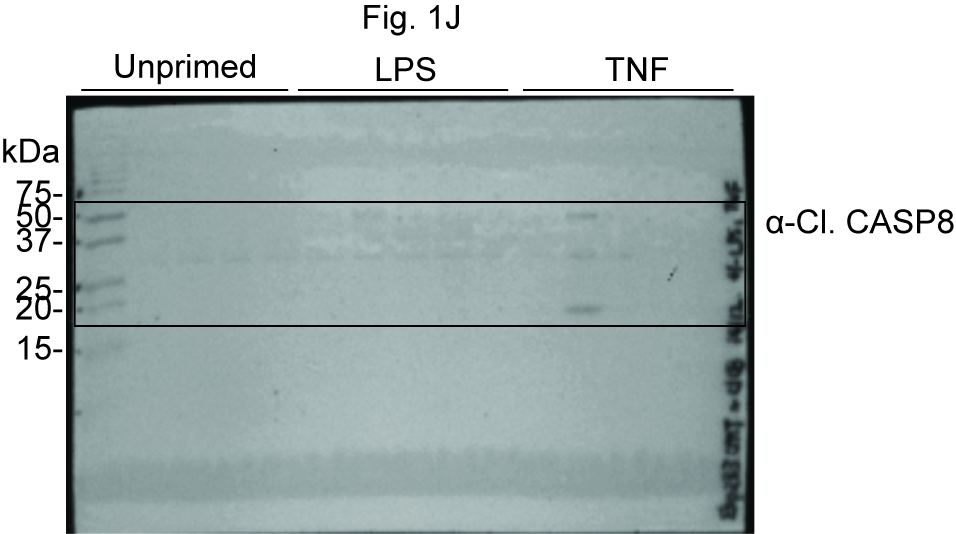

Supplement: Supplementary file 3 — Source data Fig. 1 [file 44318_2025_412_MOESM3_ESM.zip › Figure 1/1J/Fig1J Cl Casp8.tif]

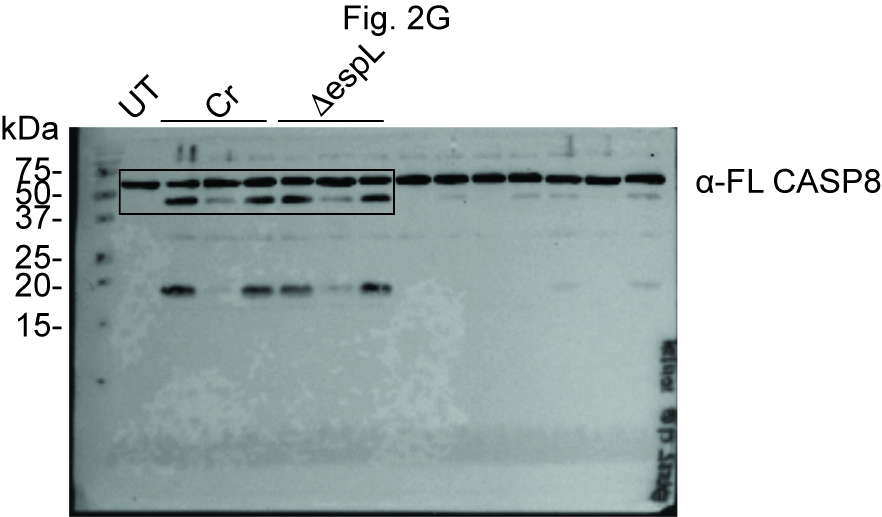

Supplement: Supplementary file 4 — Source data Fig. 2 [file 44318_2025_412_MOESM4_ESM.zip › Figure 2/2G/Fig2G FL Casp8.tif]

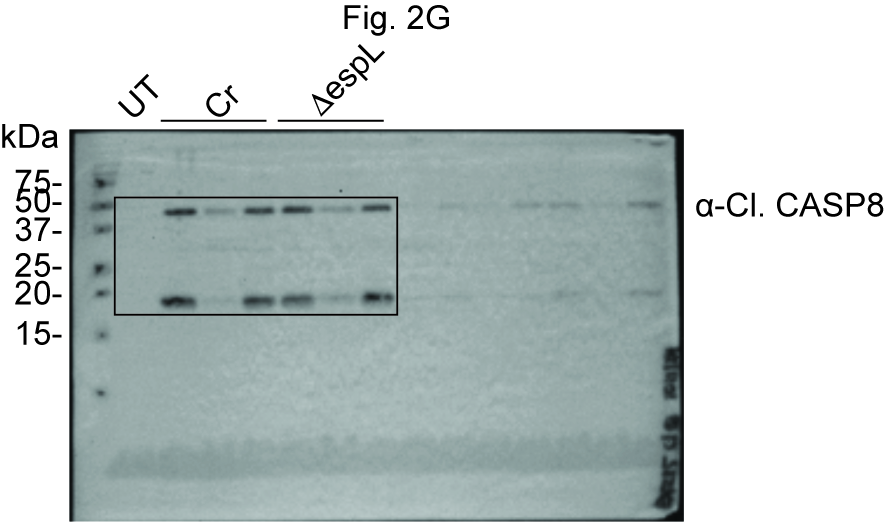

Supplement: Supplementary file 4 — Source data Fig. 2 [file 44318_2025_412_MOESM4_ESM.zip › Figure 2/2G/Fig2G Cl Casp8.tif]

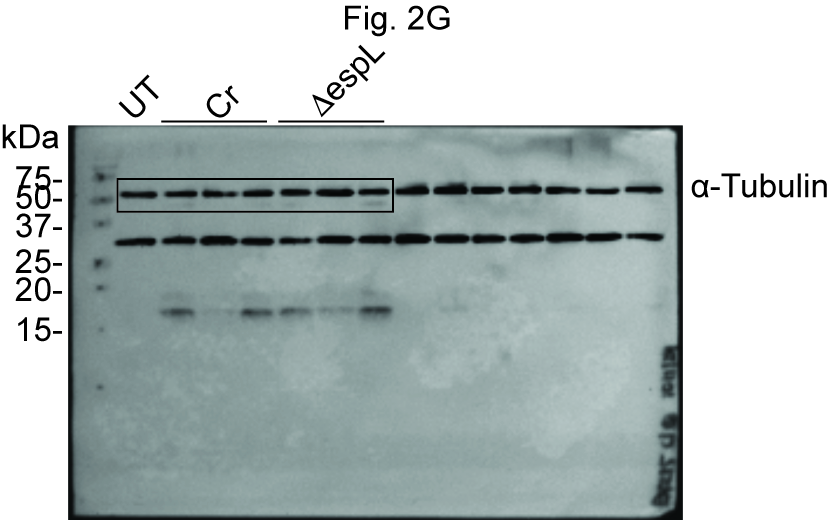

Supplement: Supplementary file 4 — Source data Fig. 2 [file 44318_2025_412_MOESM4_ESM.zip › Figure 2/2G/Fig2G Tubulin.tif]

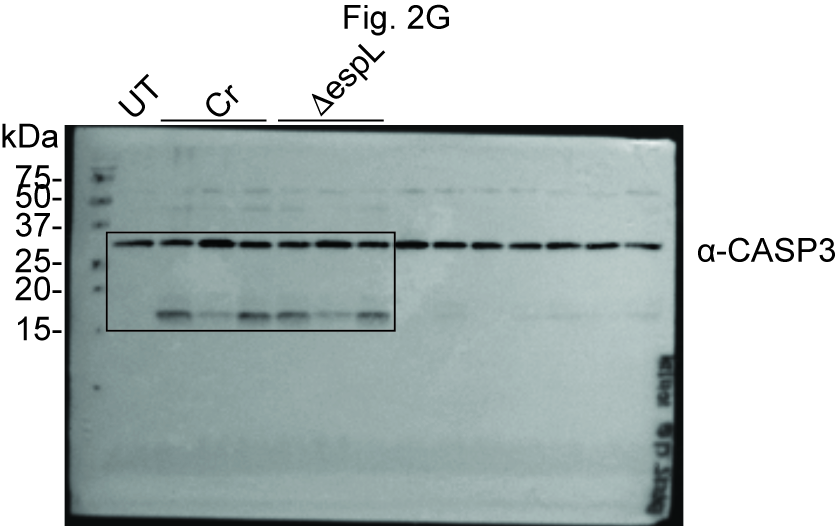

Supplement: Supplementary file 4 — Source data Fig. 2 [file 44318_2025_412_MOESM4_ESM.zip › Figure 2/2G/Fig2G Casp3.tif]

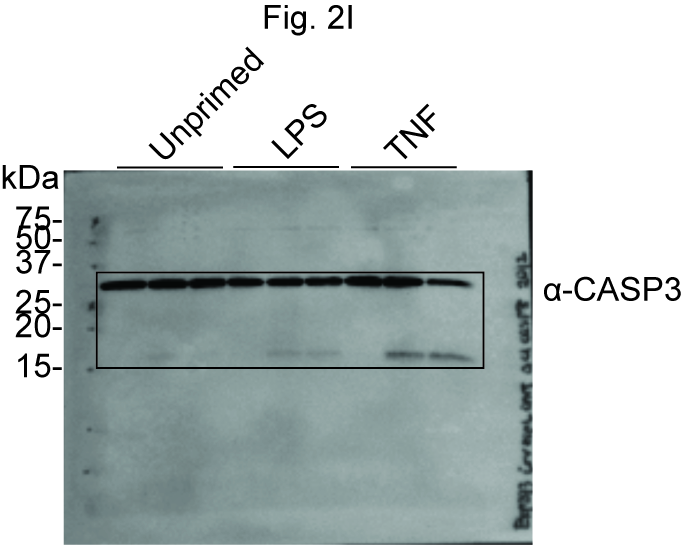

Supplement: Supplementary file 4 — Source data Fig. 2 [file 44318_2025_412_MOESM4_ESM.zip › Figure 2/2I/Fig2I Casp3.tif]

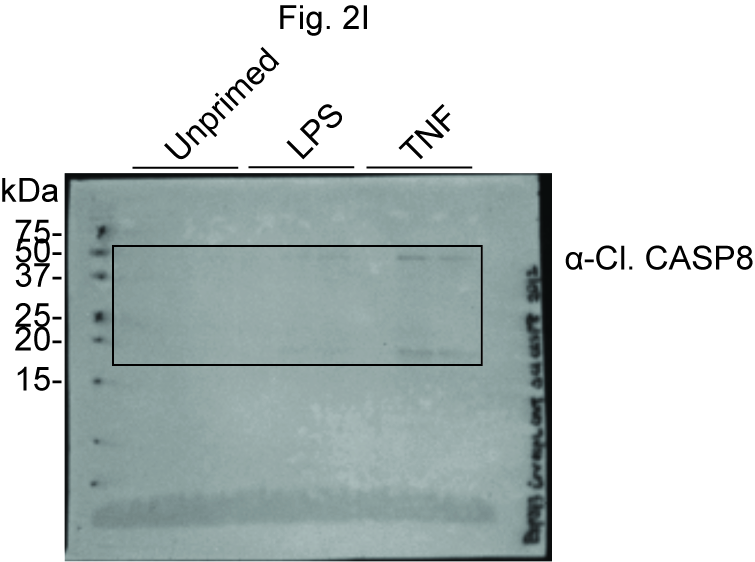

Supplement: Supplementary file 4 — Source data Fig. 2 [file 44318_2025_412_MOESM4_ESM.zip › Figure 2/2I/Fig2I Cl Casp8.tif]

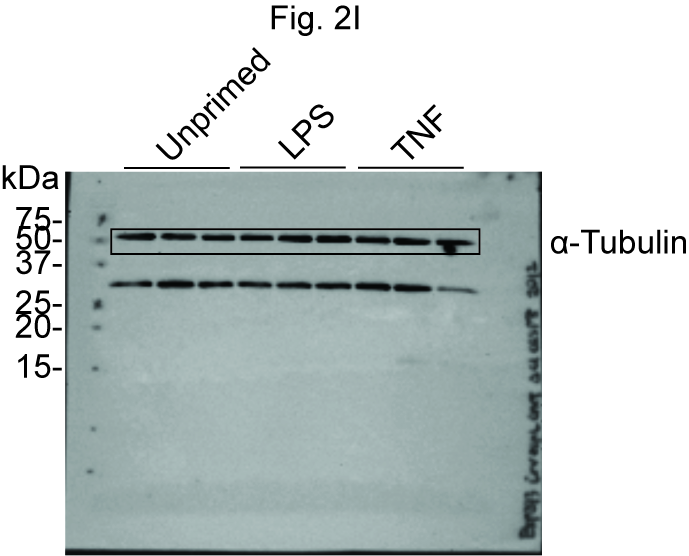

Supplement: Supplementary file 4 — Source data Fig. 2 [file 44318_2025_412_MOESM4_ESM.zip › Figure 2/2I/Fig2I Tubulin.tif]

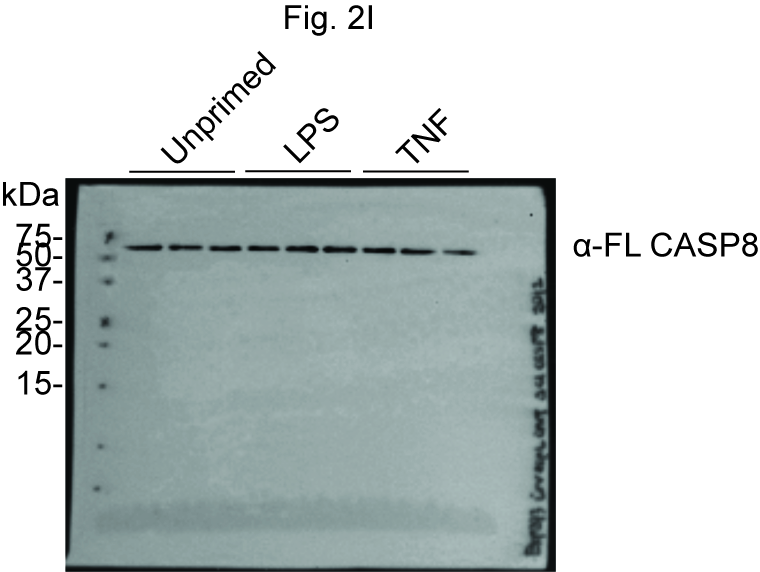

Supplement: Supplementary file 4 — Source data Fig. 2 [file 44318_2025_412_MOESM4_ESM.zip › Figure 2/2I/Fig2I FL Casp8.tif]

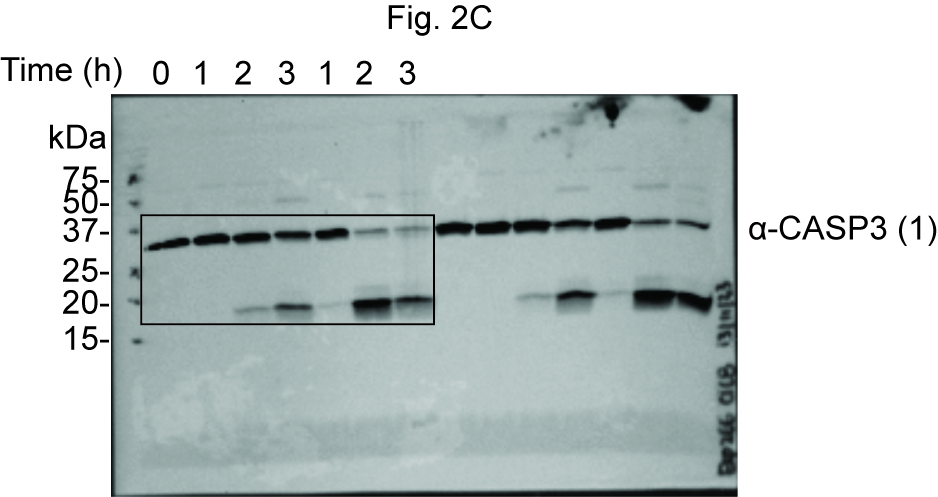

Supplement: Supplementary file 4 — Source data Fig. 2 [file 44318_2025_412_MOESM4_ESM.zip › Figure 2/2C/Fig2C Casp3.tif]

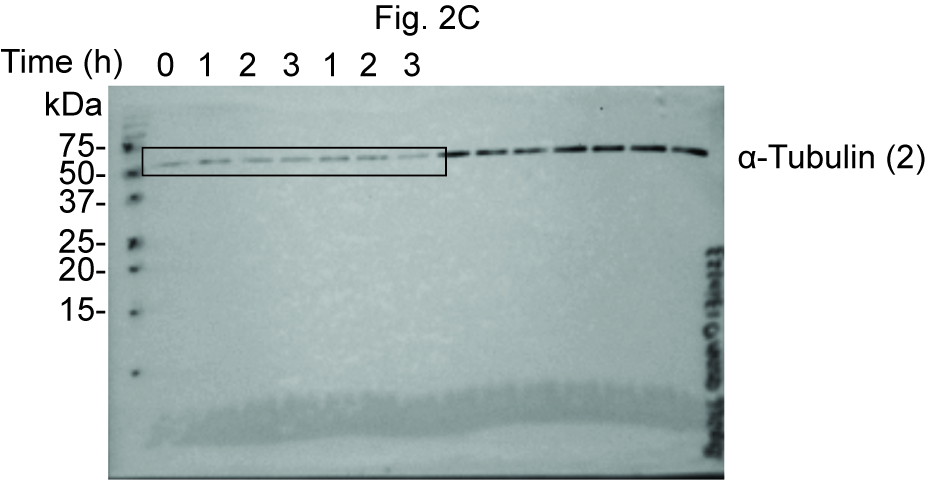

Supplement: Supplementary file 4 — Source data Fig. 2 [file 44318_2025_412_MOESM4_ESM.zip › Figure 2/2C/Fig2C Tubulin 2.tif]

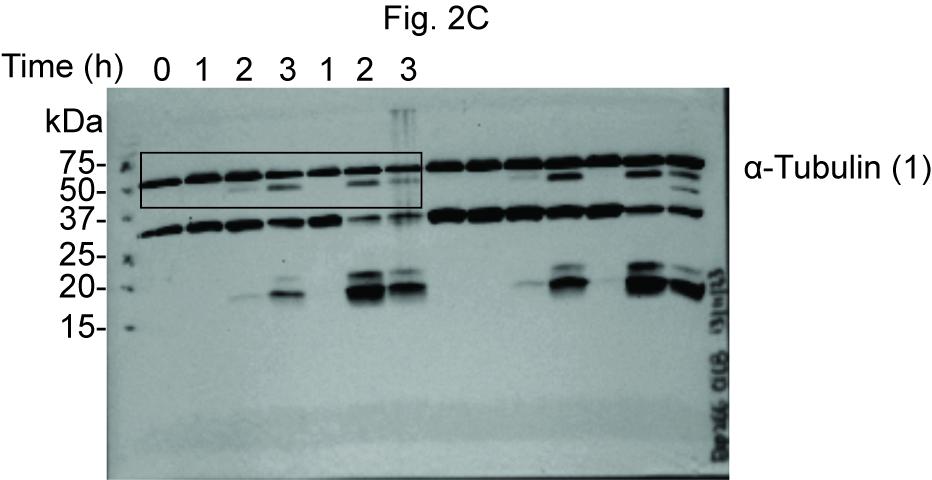

Supplement: Supplementary file 4 — Source data Fig. 2 [file 44318_2025_412_MOESM4_ESM.zip › Figure 2/2C/Fig2C Tubulin 1.tif]

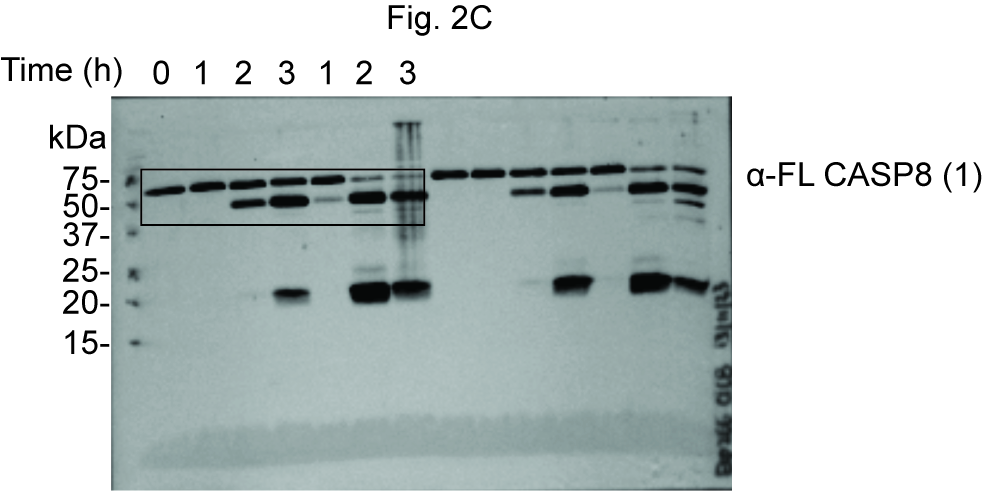

Supplement: Supplementary file 4 — Source data Fig. 2 [file 44318_2025_412_MOESM4_ESM.zip › Figure 2/2C/Fig2C FL Casp8.tif]

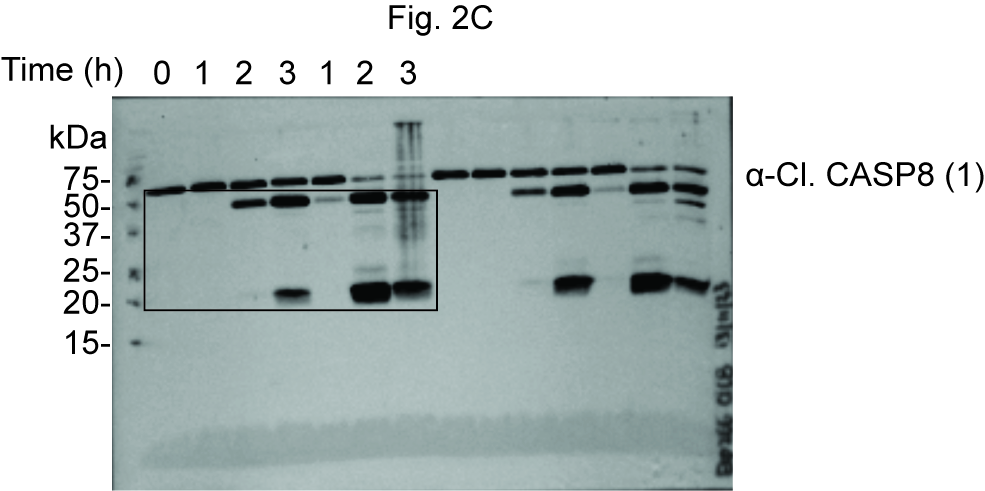

Supplement: Supplementary file 4 — Source data Fig. 2 [file 44318_2025_412_MOESM4_ESM.zip › Figure 2/2C/Fig2C Cl Casp8.tif]

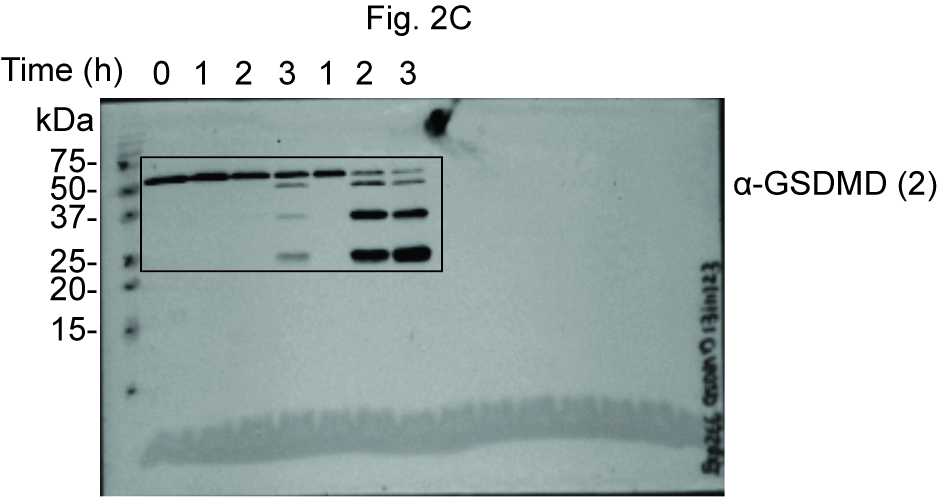

Supplement: Supplementary file 4 — Source data Fig. 2 [file 44318_2025_412_MOESM4_ESM.zip › Figure 2/2C/Fig2C GSDMD.tif]

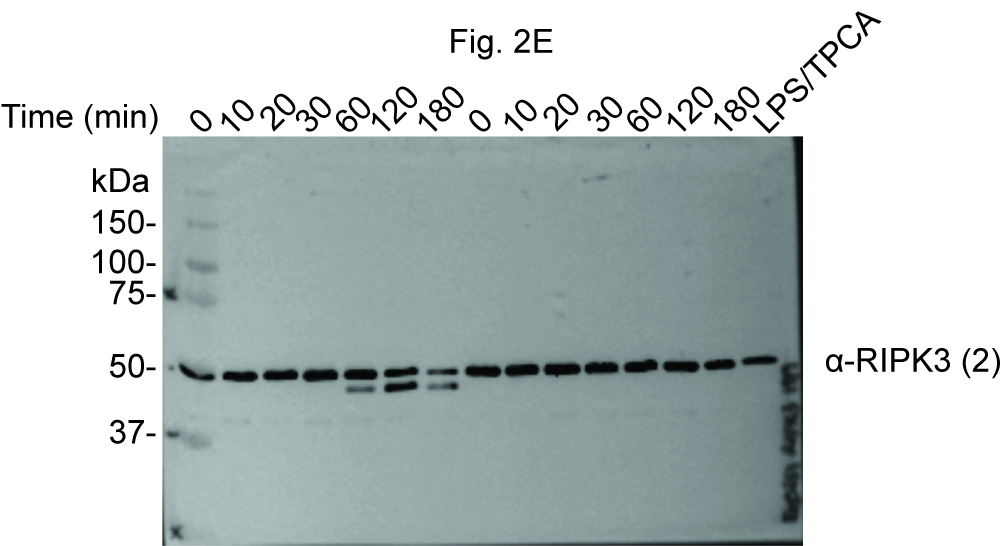

Supplement: Supplementary file 4 — Source data Fig. 2 [file 44318_2025_412_MOESM4_ESM.zip › Figure 2/2E/Fig2E RIPK3.tif]

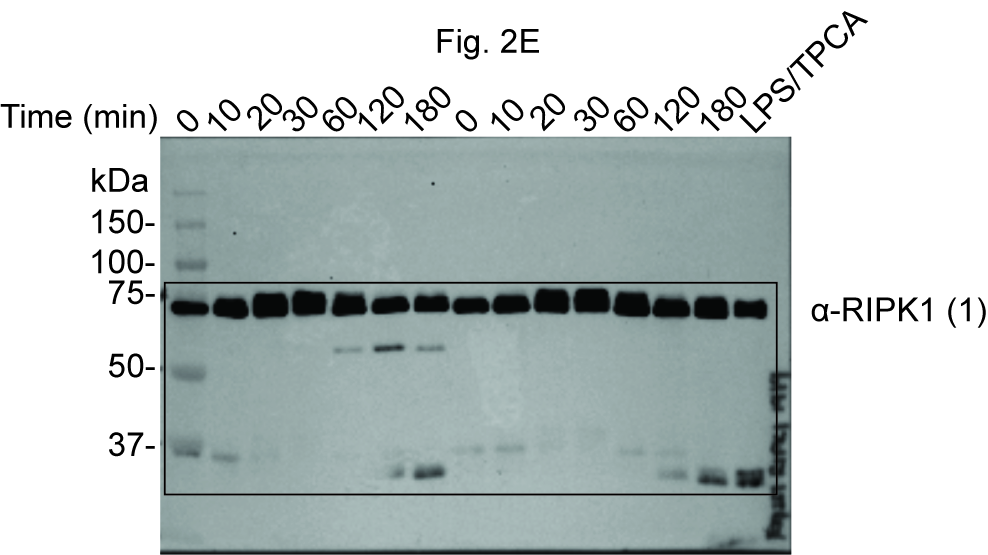

Supplement: Supplementary file 4 — Source data Fig. 2 [file 44318_2025_412_MOESM4_ESM.zip › Figure 2/2E/Fig2E RIPK1.tif]

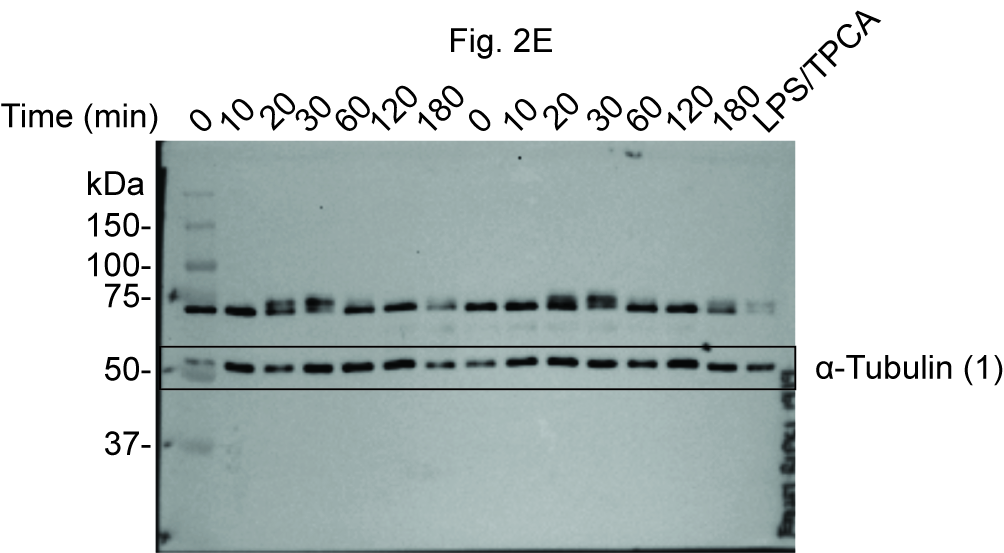

Supplement: Supplementary file 4 — Source data Fig. 2 [file 44318_2025_412_MOESM4_ESM.zip › Figure 2/2E/Fig2E Tubulin 1.tif]

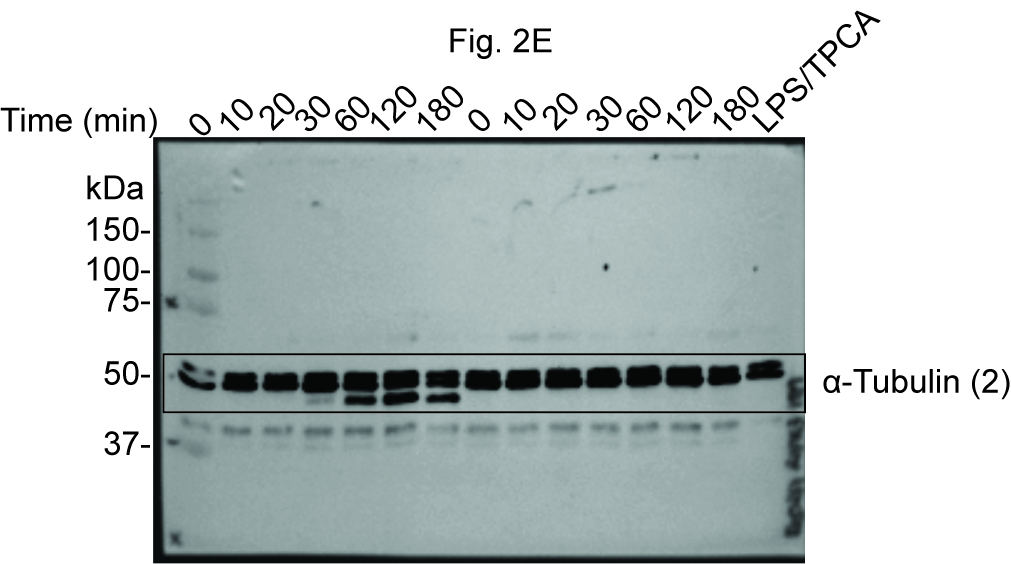

Supplement: Supplementary file 4 — Source data Fig. 2 [file 44318_2025_412_MOESM4_ESM.zip › Figure 2/2E/Fig2E Tubulin 2.tif]

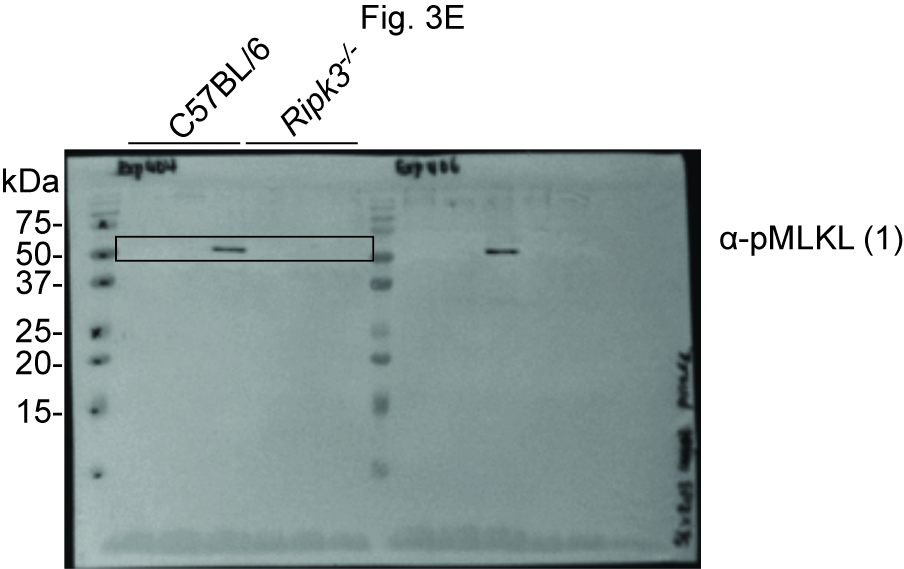

Supplement: Supplementary file 5 — Source data Fig. 3 [file 44318_2025_412_MOESM5_ESM.zip › Figure 3/3E/Fig3E pMLKL.tif]

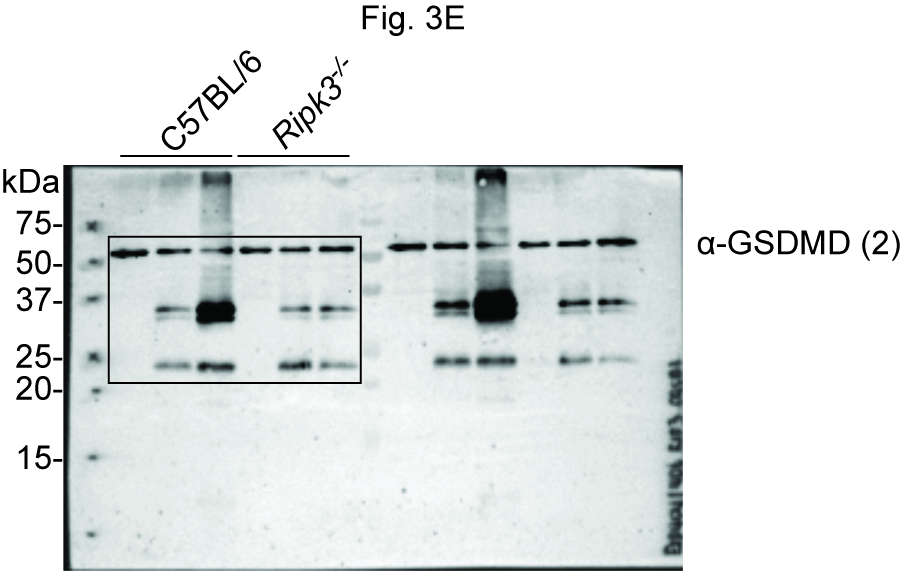

Supplement: Supplementary file 5 — Source data Fig. 3 [file 44318_2025_412_MOESM5_ESM.zip › Figure 3/3E/Fig3E GSDMD.tif]

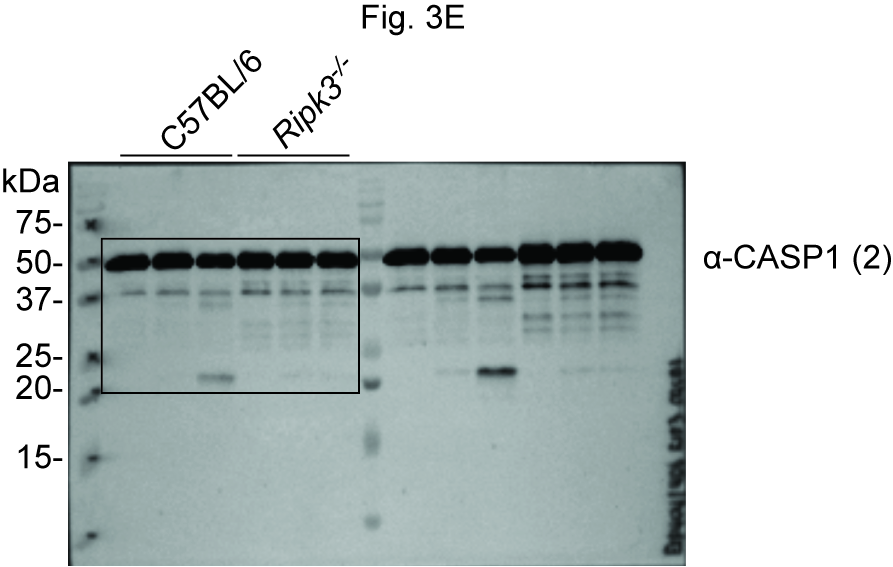

Supplement: Supplementary file 5 — Source data Fig. 3 [file 44318_2025_412_MOESM5_ESM.zip › Figure 3/3E/Fig3E Casp1.tif]

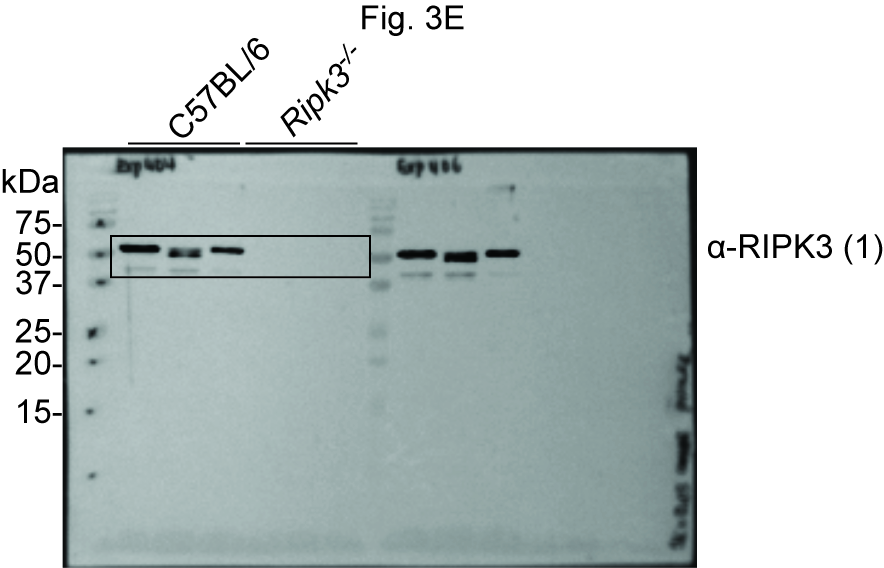

Supplement: Supplementary file 5 — Source data Fig. 3 [file 44318_2025_412_MOESM5_ESM.zip › Figure 3/3E/Fig3E RIPK3.tif]

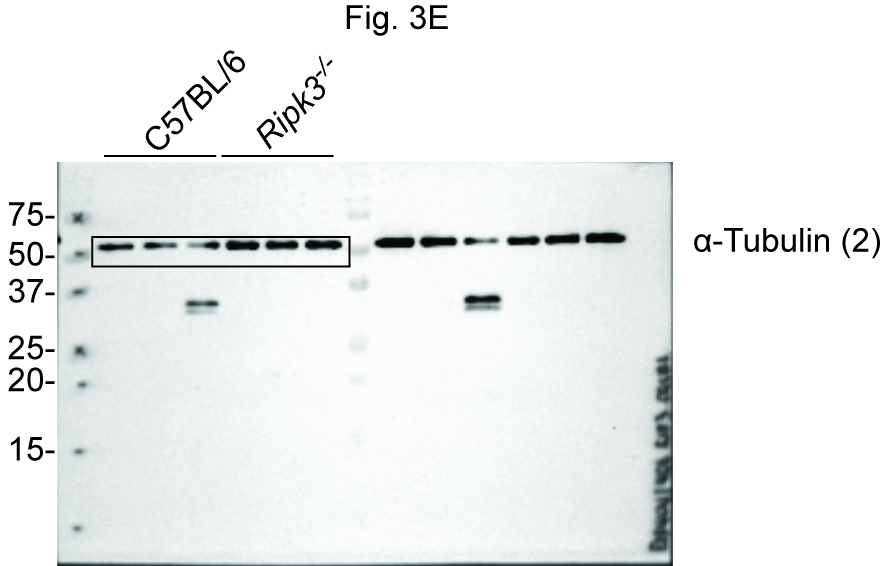

Supplement: Supplementary file 5 — Source data Fig. 3 [file 44318_2025_412_MOESM5_ESM.zip › Figure 3/3E/Fig3E Tubulin 2.tif]

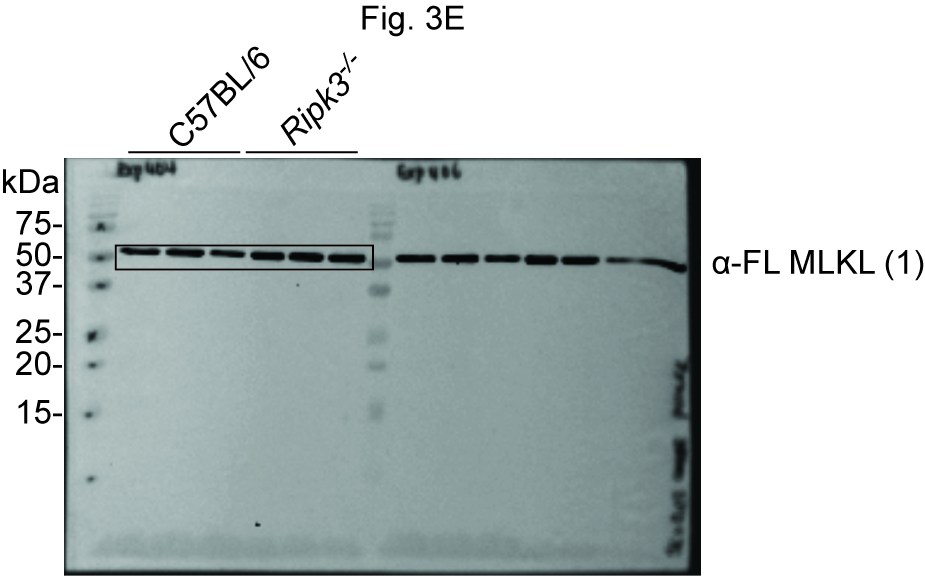

Supplement: Supplementary file 5 — Source data Fig. 3 [file 44318_2025_412_MOESM5_ESM.zip › Figure 3/3E/Fig3E MLKL.tif]

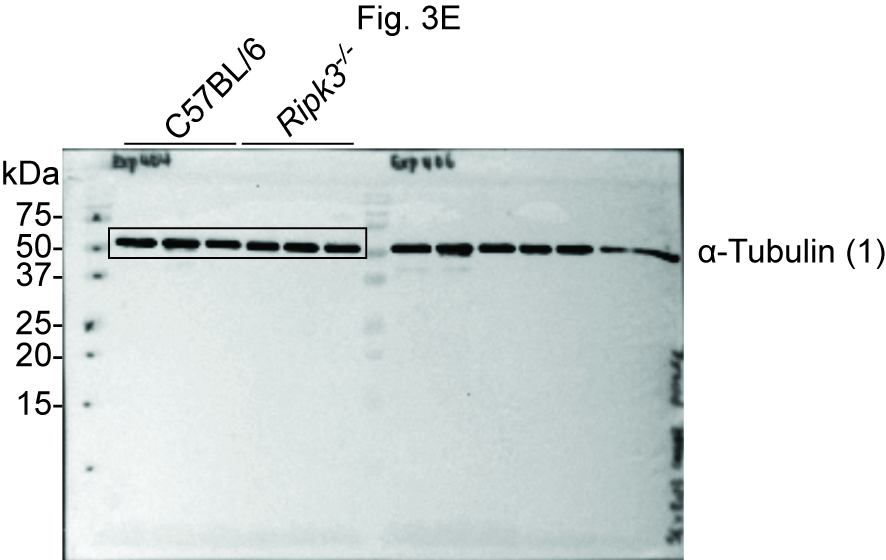

Supplement: Supplementary file 5 — Source data Fig. 3 [file 44318_2025_412_MOESM5_ESM.zip › Figure 3/3E/Fig3E Tubulin 1.tif]

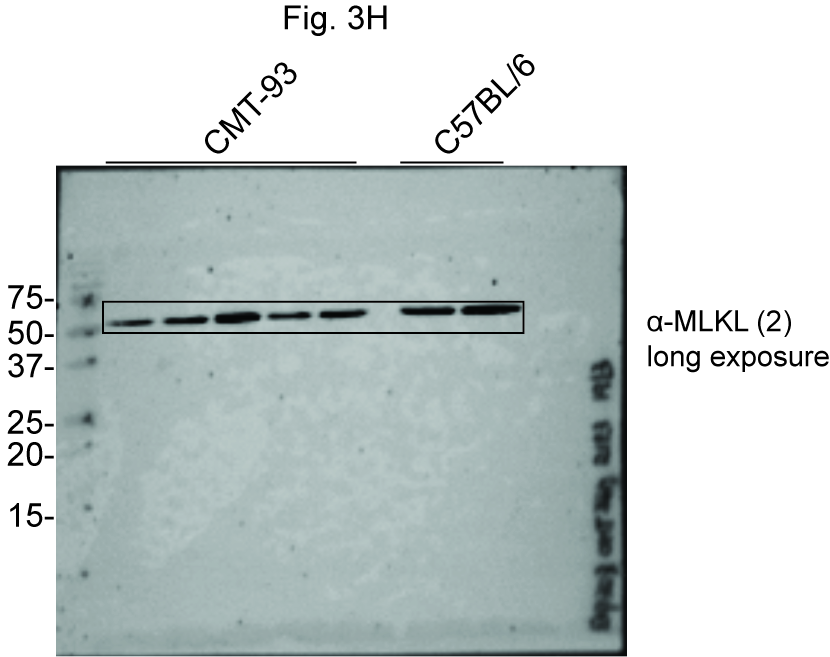

Supplement: Supplementary file 5 — Source data Fig. 3 [file 44318_2025_412_MOESM5_ESM.zip › Figure 3/3H/Fig3H MLKL long exposure.tif]

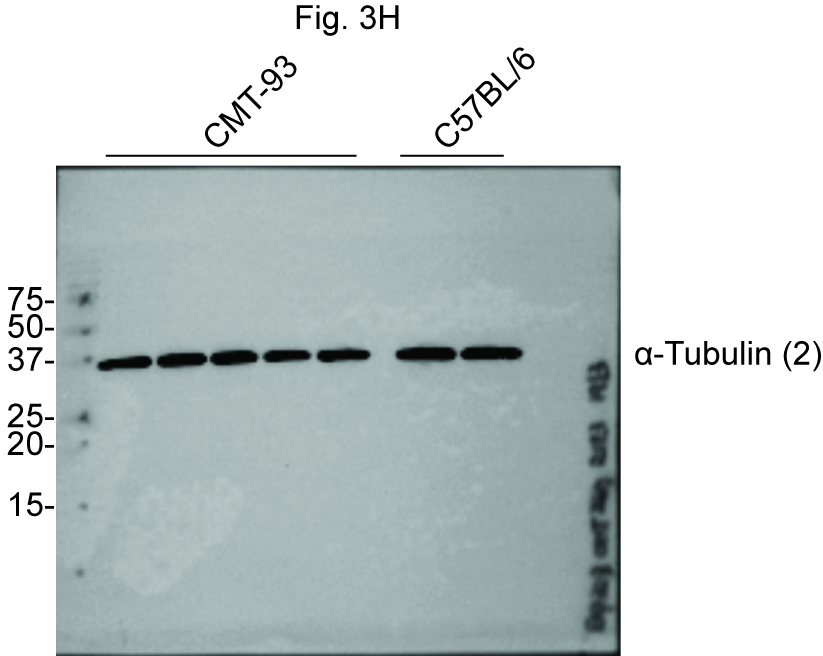

Supplement: Supplementary file 5 — Source data Fig. 3 [file 44318_2025_412_MOESM5_ESM.zip › Figure 3/3H/Fig3H Tubulin 2.tif]

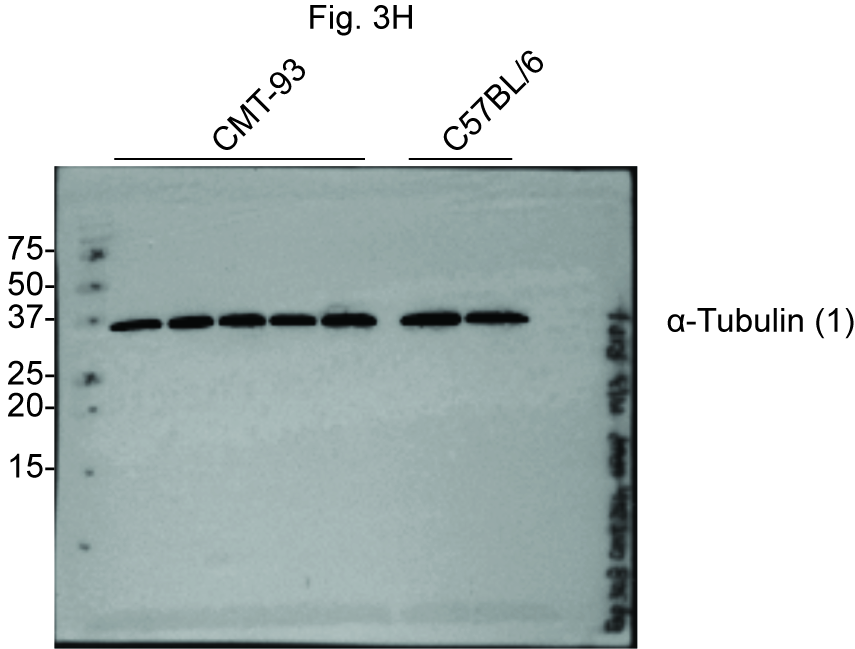

Supplement: Supplementary file 5 — Source data Fig. 3 [file 44318_2025_412_MOESM5_ESM.zip › Figure 3/3H/Fig3H Tubulin 1.tif]

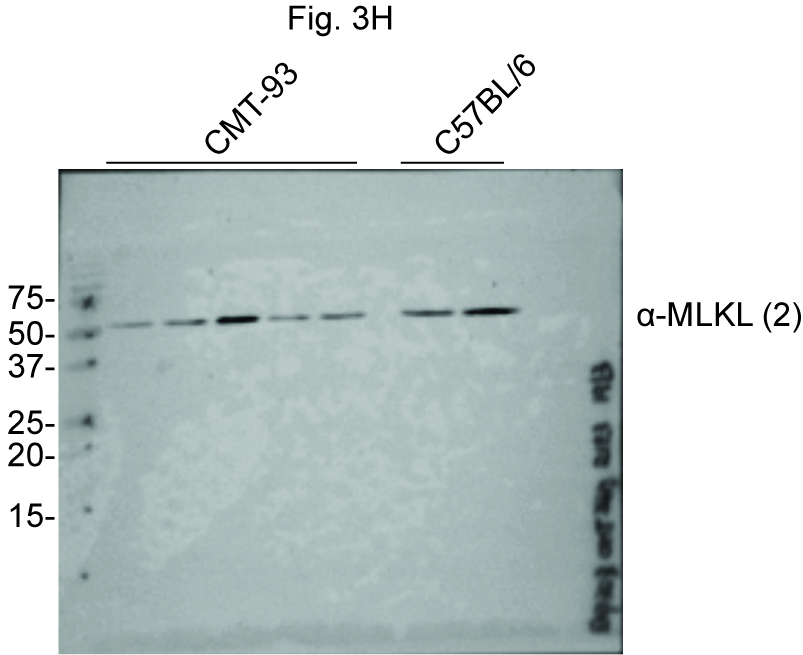

Supplement: Supplementary file 5 — Source data Fig. 3 [file 44318_2025_412_MOESM5_ESM.zip › Figure 3/3H/Fig3H MLKL.tif]

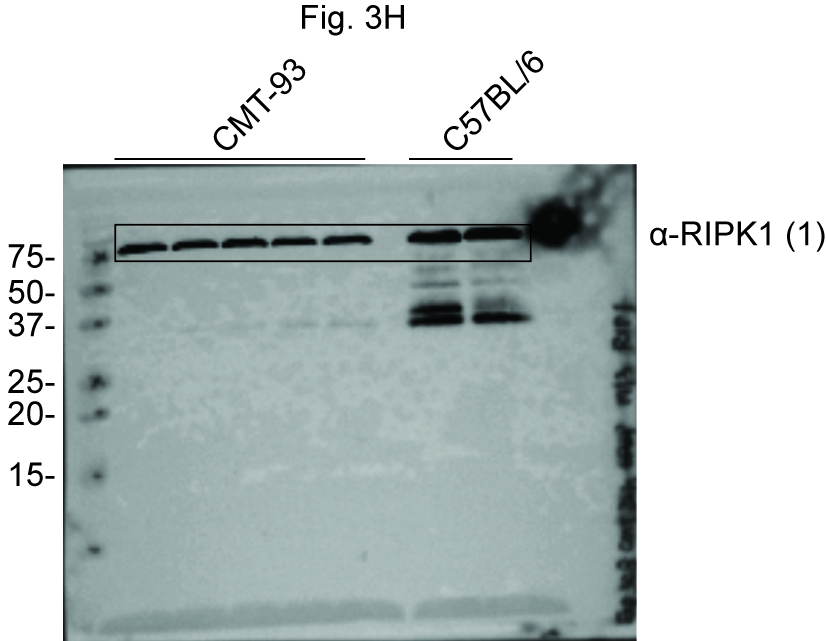

Supplement: Supplementary file 5 — Source data Fig. 3 [file 44318_2025_412_MOESM5_ESM.zip › Figure 3/3H/Fig3H RIPK1.tif]

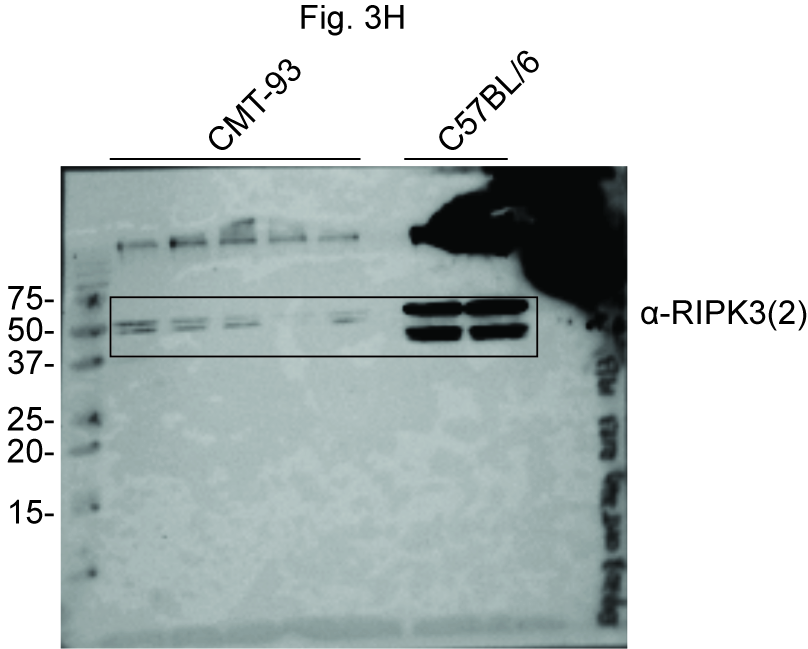

Supplement: Supplementary file 5 — Source data Fig. 3 [file 44318_2025_412_MOESM5_ESM.zip › Figure 3/3H/Fig3H RIPK3.tif]

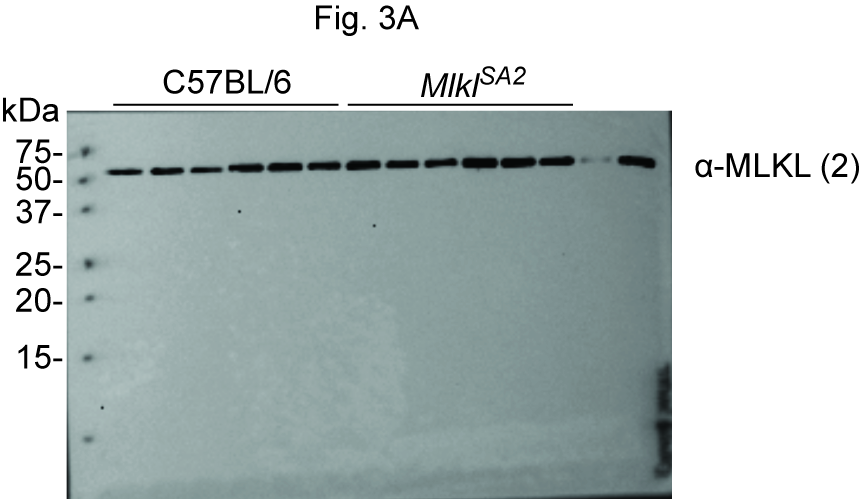

Supplement: Supplementary file 5 — Source data Fig. 3 [file 44318_2025_412_MOESM5_ESM.zip › Figure 3/3A/Fig3A MLKL.tif]

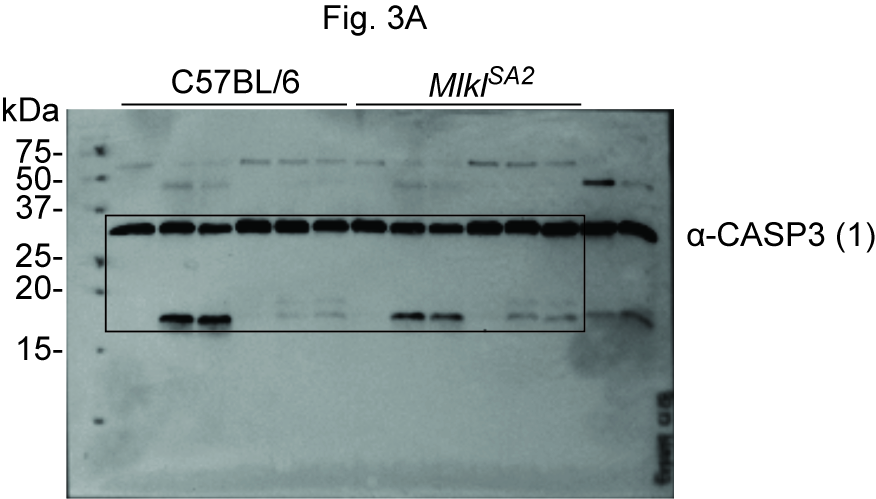

Supplement: Supplementary file 5 — Source data Fig. 3 [file 44318_2025_412_MOESM5_ESM.zip › Figure 3/3A/Fig3A Casp3.tif]

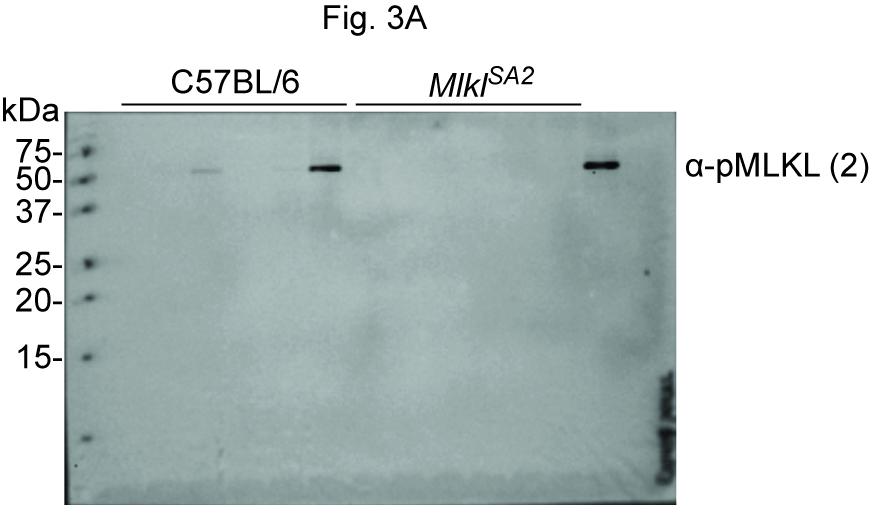

Supplement: Supplementary file 5 — Source data Fig. 3 [file 44318_2025_412_MOESM5_ESM.zip › Figure 3/3A/Fig3A pMLKL.tif]

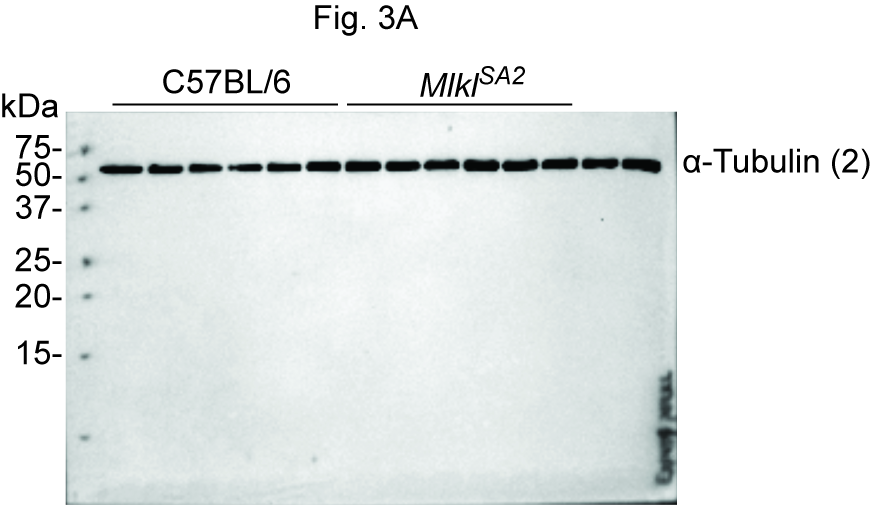

Supplement: Supplementary file 5 — Source data Fig. 3 [file 44318_2025_412_MOESM5_ESM.zip › Figure 3/3A/Fig3A Tubulin 2.tif]

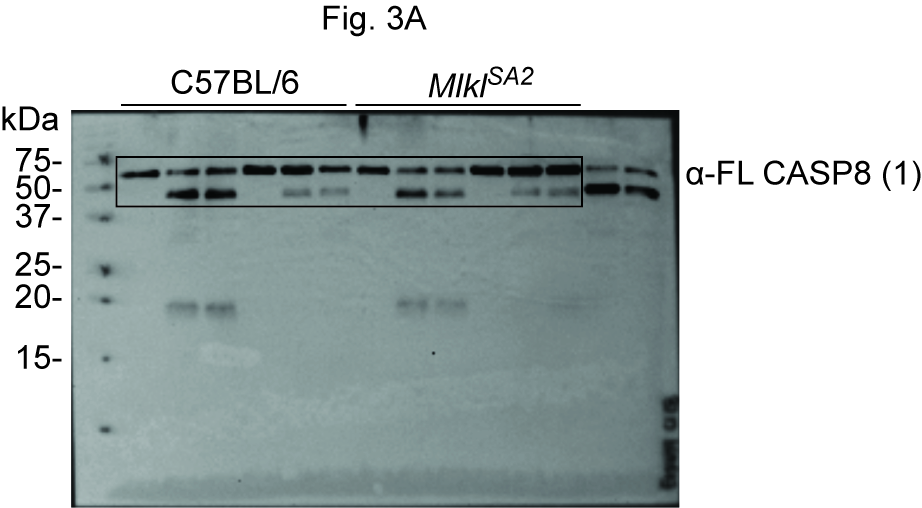

Supplement: Supplementary file 5 — Source data Fig. 3 [file 44318_2025_412_MOESM5_ESM.zip › Figure 3/3A/Fig3A FL Casp8.tif]

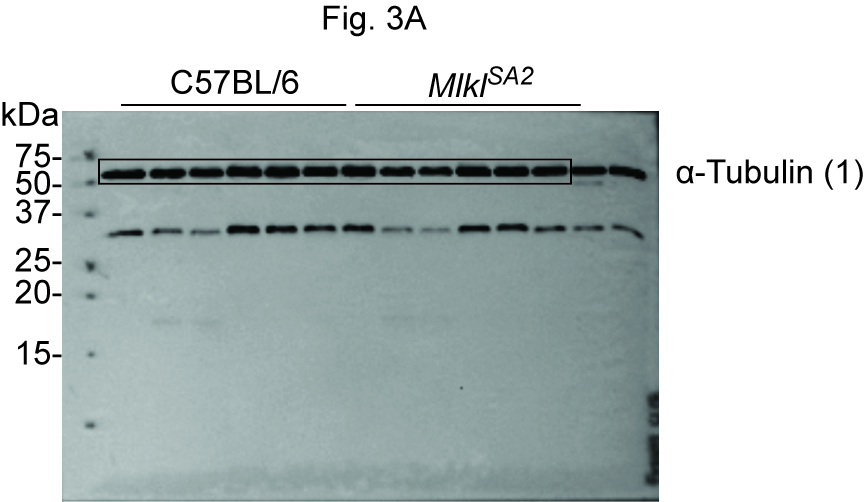

Supplement: Supplementary file 5 — Source data Fig. 3 [file 44318_2025_412_MOESM5_ESM.zip › Figure 3/3A/Fig3A Tubulin 1.tif]

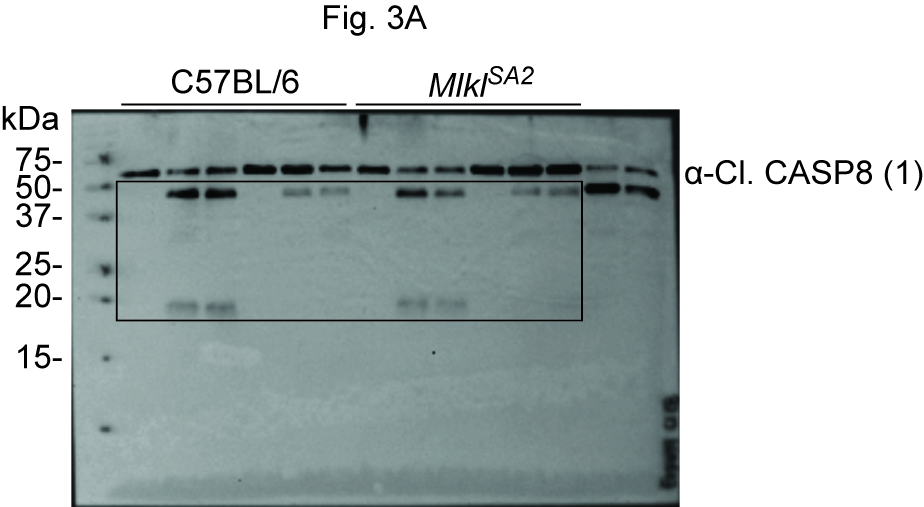

Supplement: Supplementary file 5 — Source data Fig. 3 [file 44318_2025_412_MOESM5_ESM.zip › Figure 3/3A/Fig3A Cl Casp8.tif]

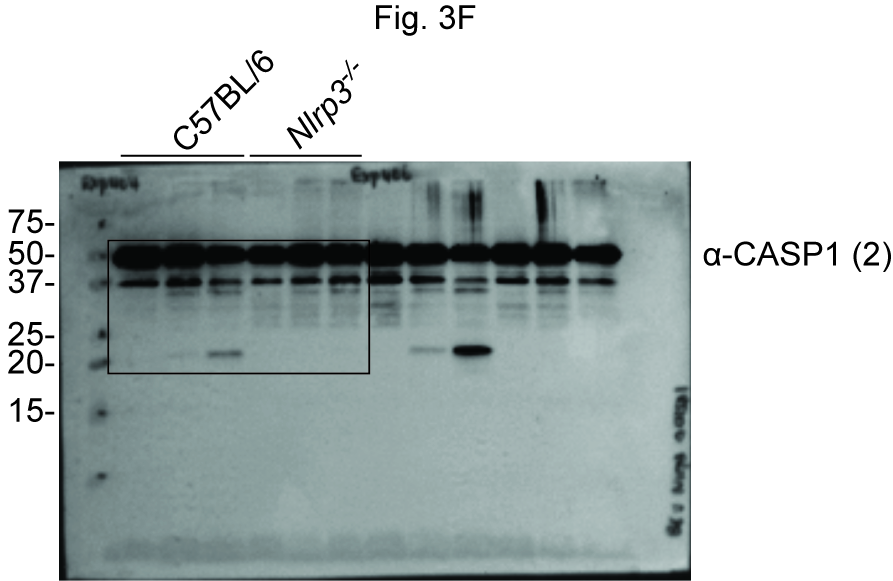

Supplement: Supplementary file 5 — Source data Fig. 3 [file 44318_2025_412_MOESM5_ESM.zip › Figure 3/3F/Fig3F Casp1.tif]

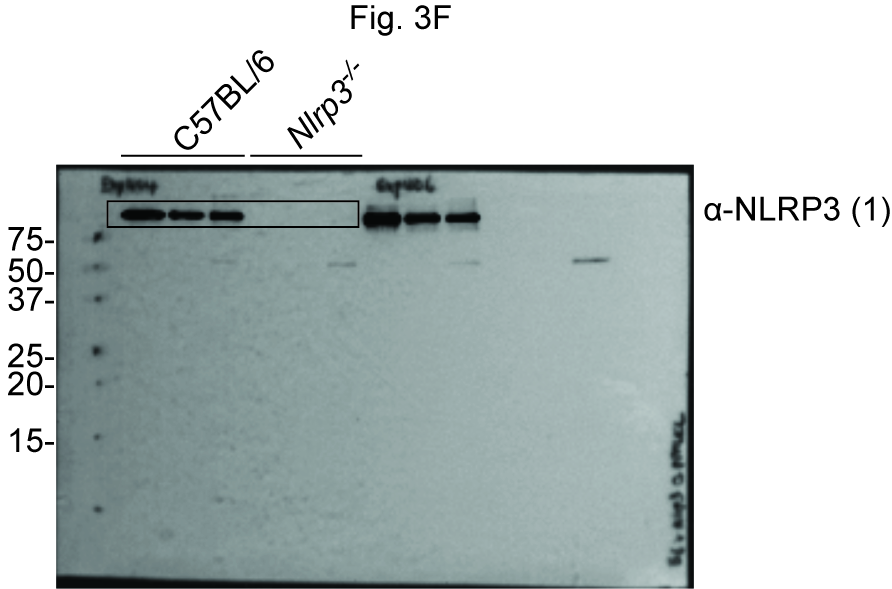

Supplement: Supplementary file 5 — Source data Fig. 3 [file 44318_2025_412_MOESM5_ESM.zip › Figure 3/3F/Fig3F NLRP3.tif]

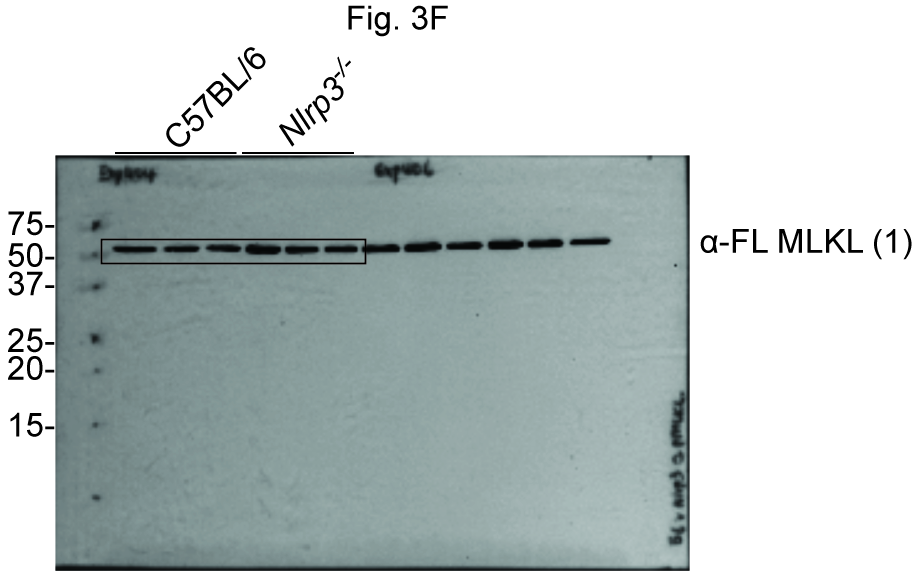

Supplement: Supplementary file 5 — Source data Fig. 3 [file 44318_2025_412_MOESM5_ESM.zip › Figure 3/3F/Fig3F MLKL.tif]

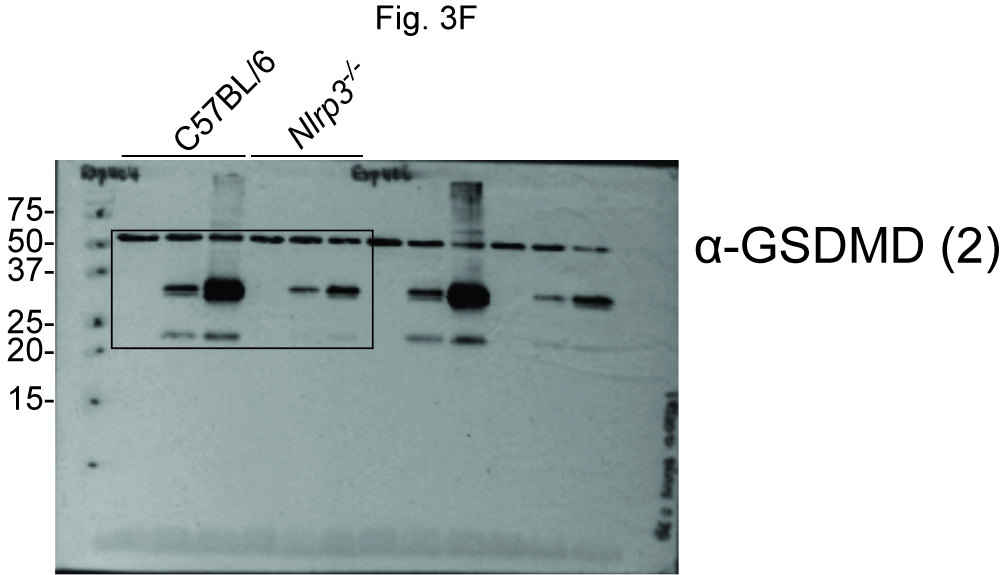

Supplement: Supplementary file 5 — Source data Fig. 3 [file 44318_2025_412_MOESM5_ESM.zip › Figure 3/3F/Fig3F GSDMD.tif]

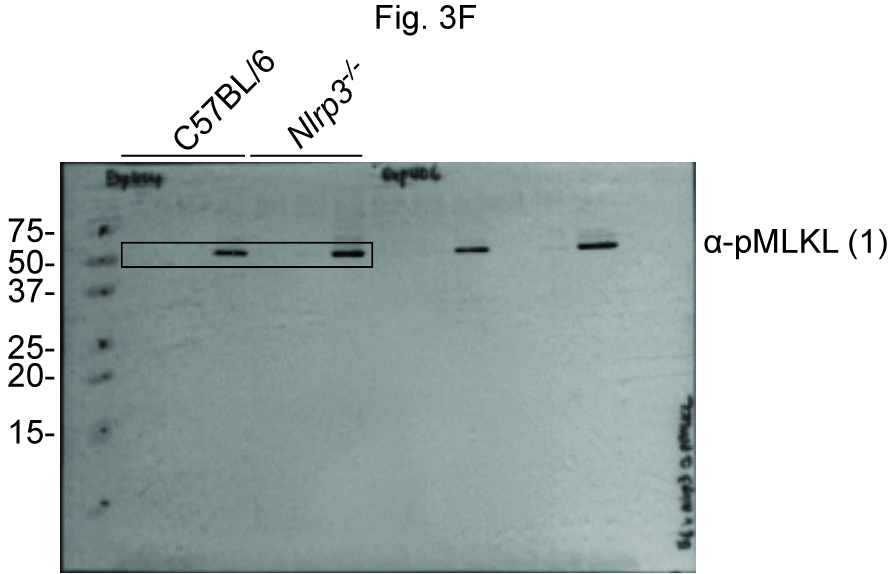

Supplement: Supplementary file 5 — Source data Fig. 3 [file 44318_2025_412_MOESM5_ESM.zip › Figure 3/3F/Fig3F pMLKL.tif]

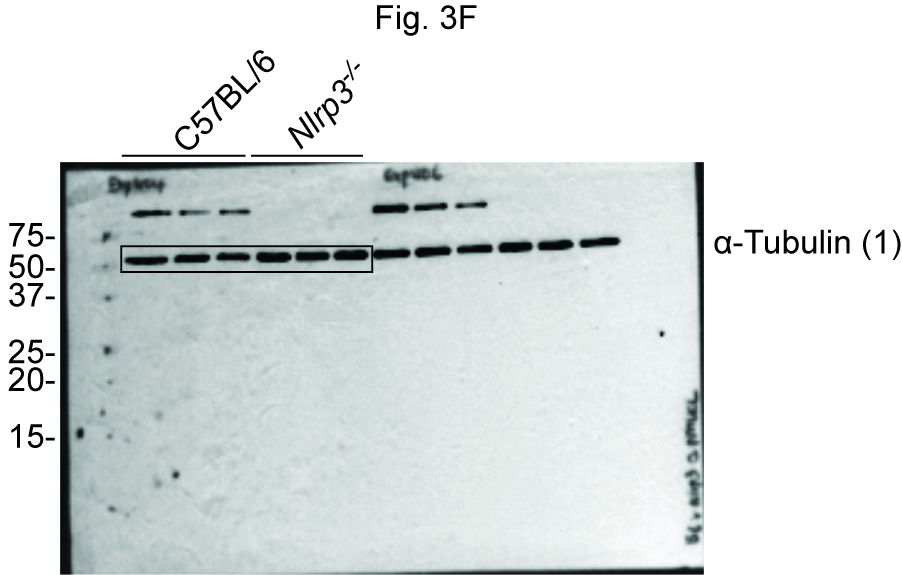

Supplement: Supplementary file 5 — Source data Fig. 3 [file 44318_2025_412_MOESM5_ESM.zip › Figure 3/3F/Fig3F Tubulin 1.tif]

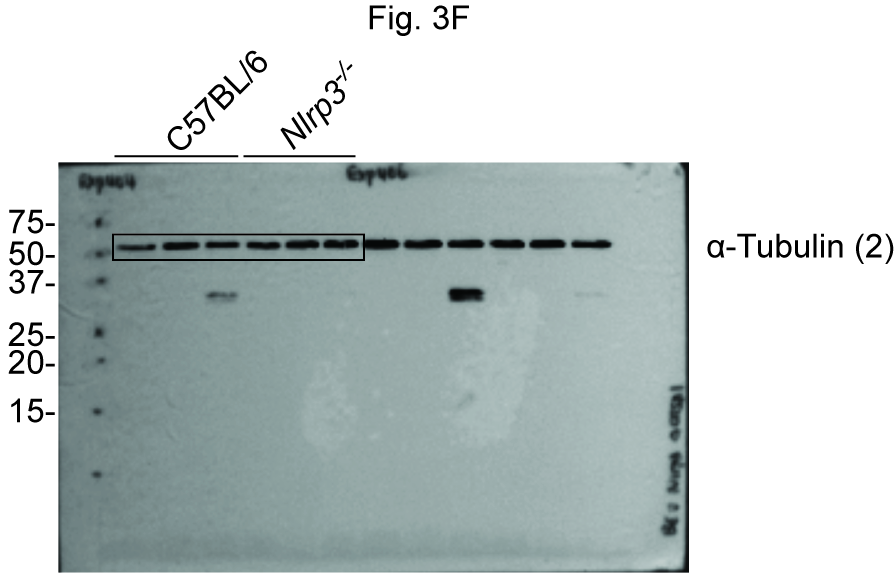

Supplement: Supplementary file 5 — Source data Fig. 3 [file 44318_2025_412_MOESM5_ESM.zip › Figure 3/3F/Fig3F Tubulin 2.tif]

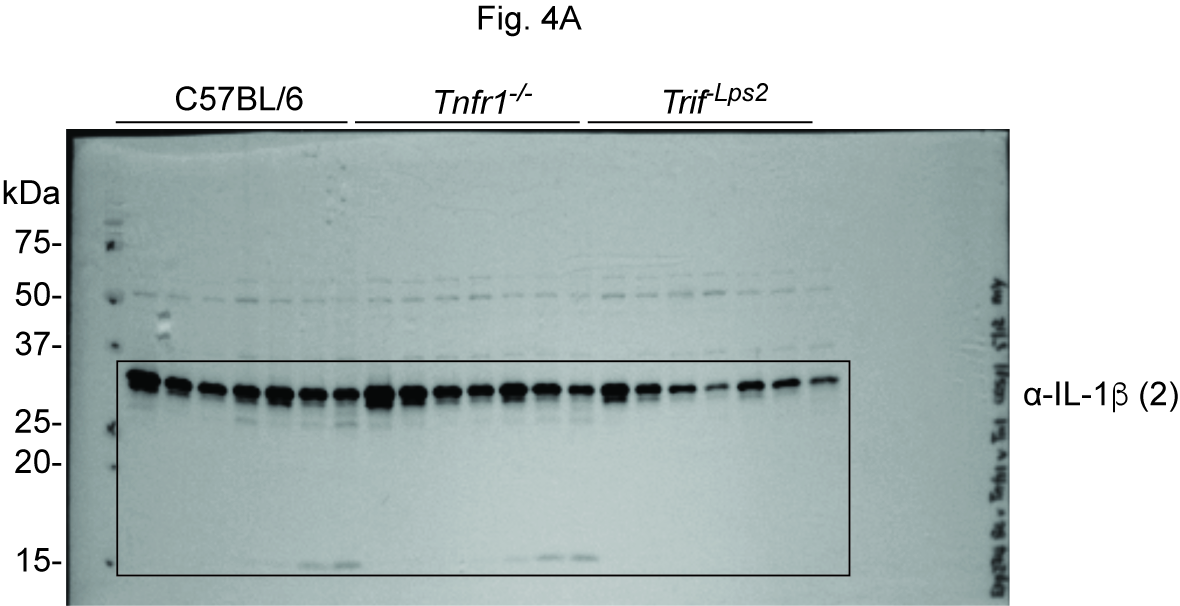

Supplement: Supplementary file 6 — Source data Fig. 4 [file 44318_2025_412_MOESM6_ESM.zip › Figure 4/4A/Fig4A IL-1b.tif]

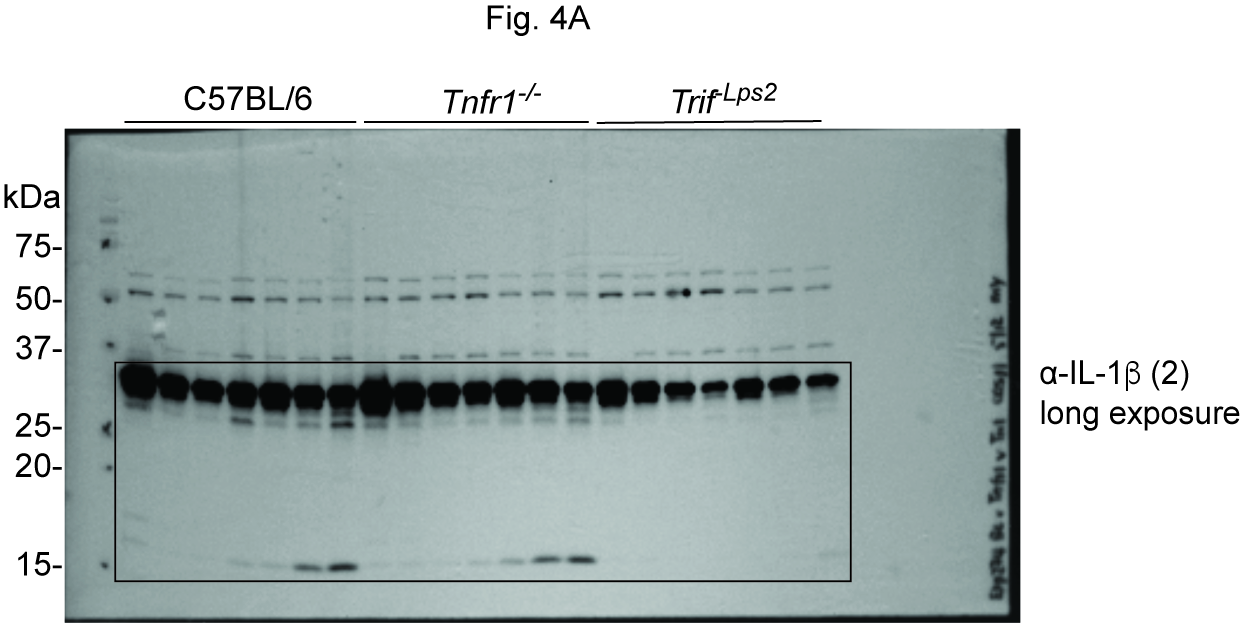

Supplement: Supplementary file 6 — Source data Fig. 4 [file 44318_2025_412_MOESM6_ESM.zip › Figure 4/4A/Fig4A IL-1b long exposure.tif]

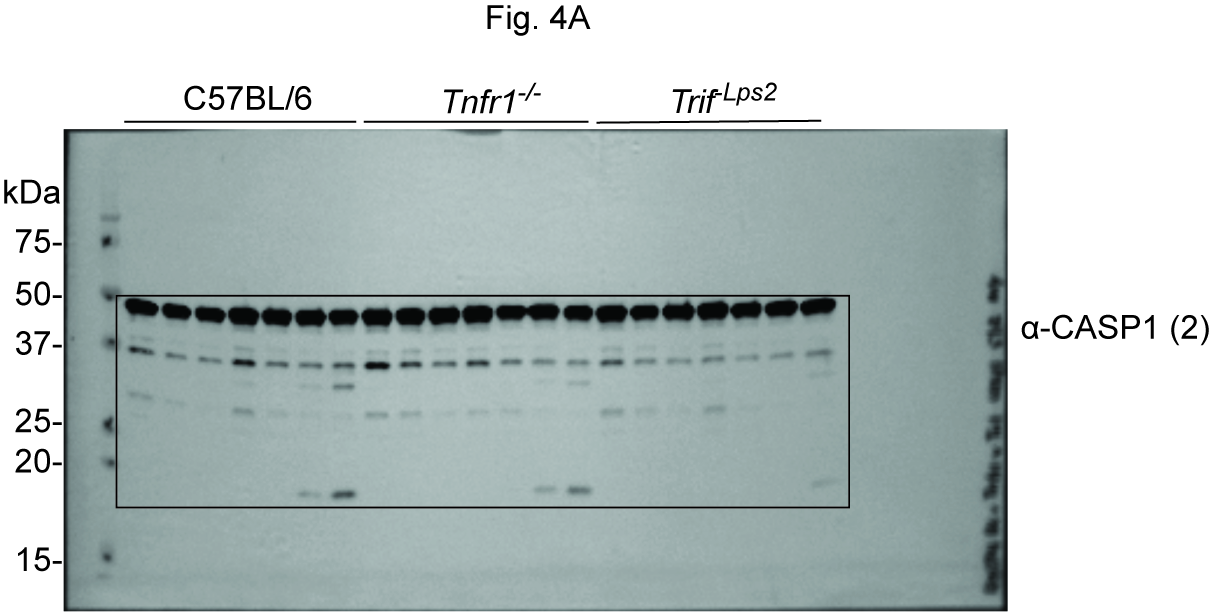

Supplement: Supplementary file 6 — Source data Fig. 4 [file 44318_2025_412_MOESM6_ESM.zip › Figure 4/4A/Fig4A Casp1.tif]

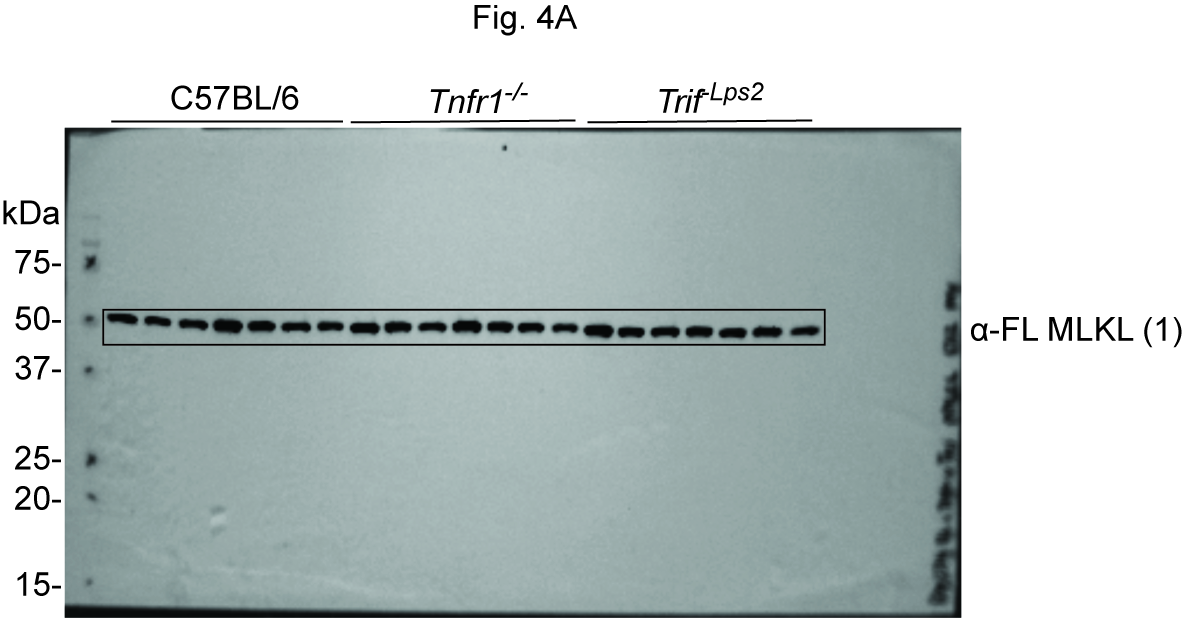

Supplement: Supplementary file 6 — Source data Fig. 4 [file 44318_2025_412_MOESM6_ESM.zip › Figure 4/4A/Fig4A MLKL.tif]

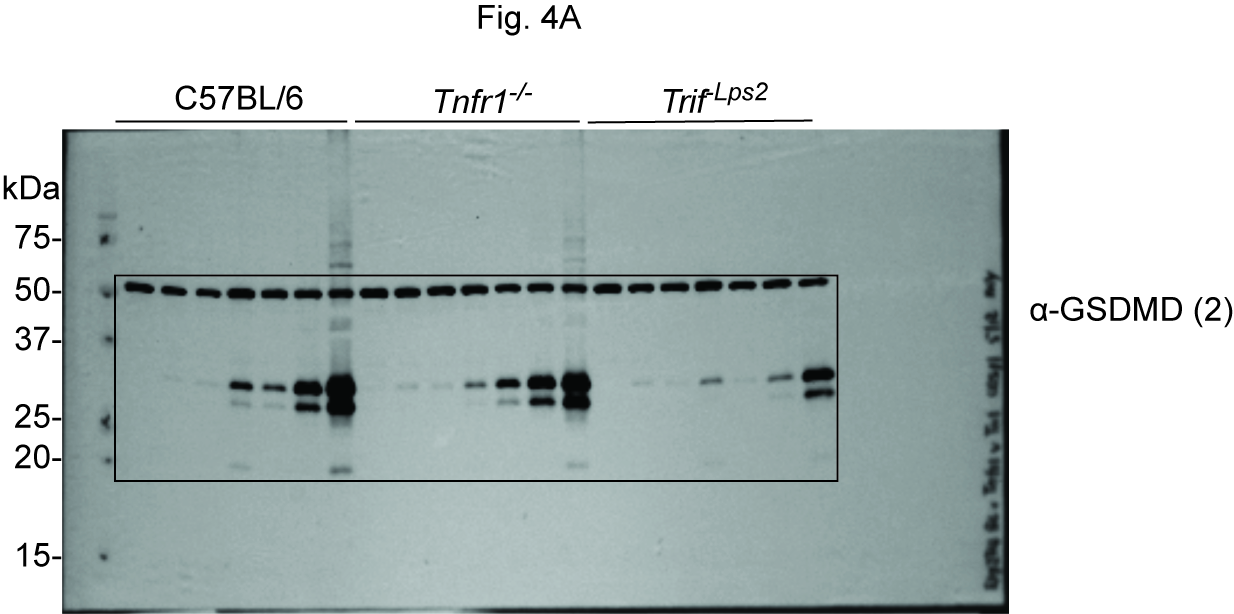

Supplement: Supplementary file 6 — Source data Fig. 4 [file 44318_2025_412_MOESM6_ESM.zip › Figure 4/4A/Fig4A GSDMD.tif]

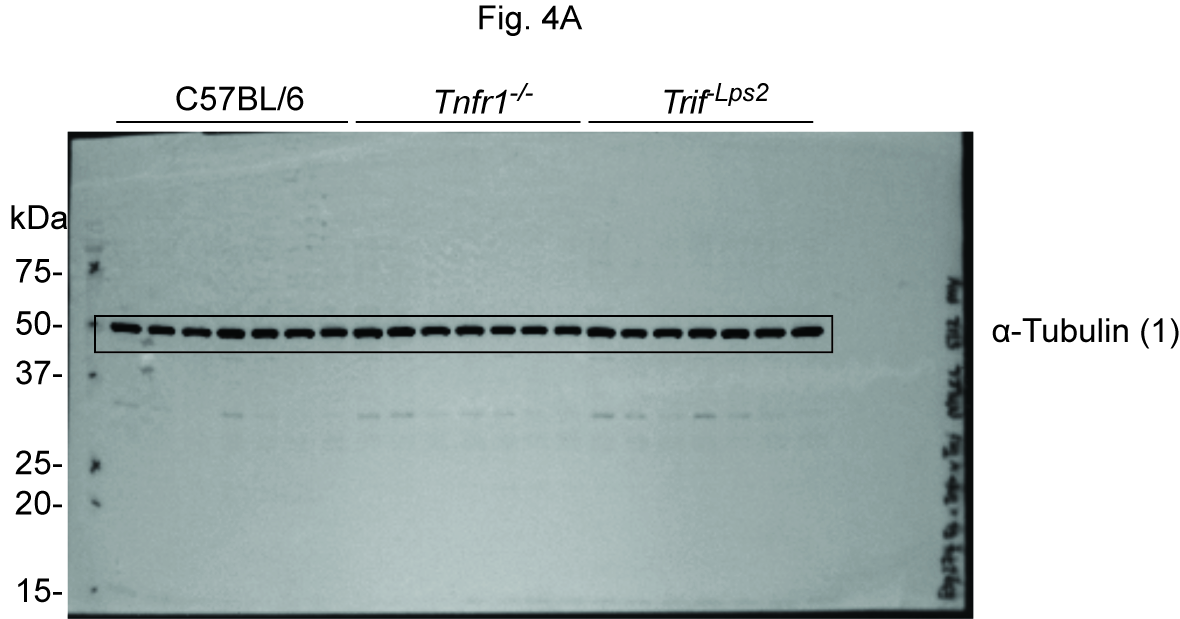

Supplement: Supplementary file 6 — Source data Fig. 4 [file 44318_2025_412_MOESM6_ESM.zip › Figure 4/4A/Fig4A Tubulin 1.tif]

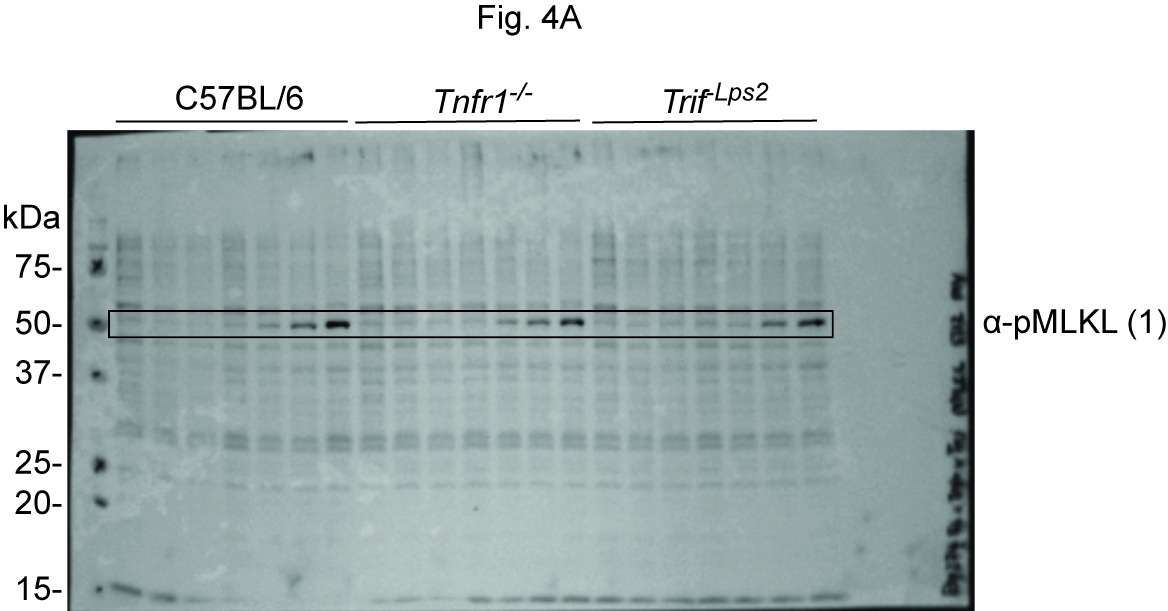

Supplement: Supplementary file 6 — Source data Fig. 4 [file 44318_2025_412_MOESM6_ESM.zip › Figure 4/4A/Fig4A pMLKL.tif]

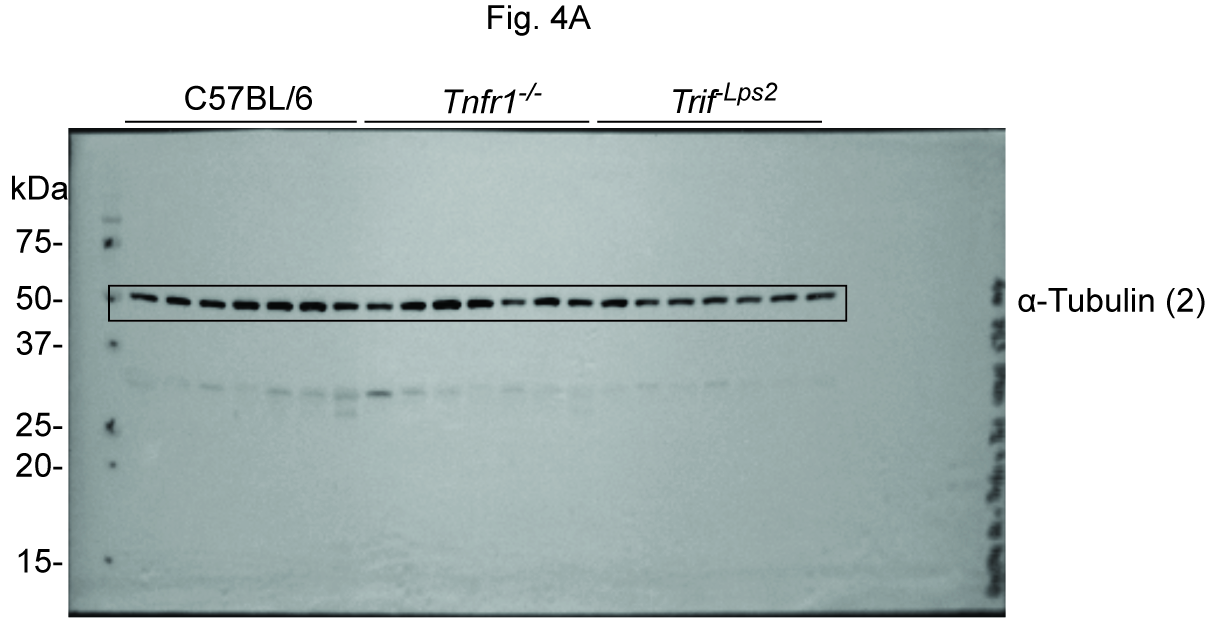

Supplement: Supplementary file 6 — Source data Fig. 4 [file 44318_2025_412_MOESM6_ESM.zip › Figure 4/4A/Fig4A Tubulin 2.tif]

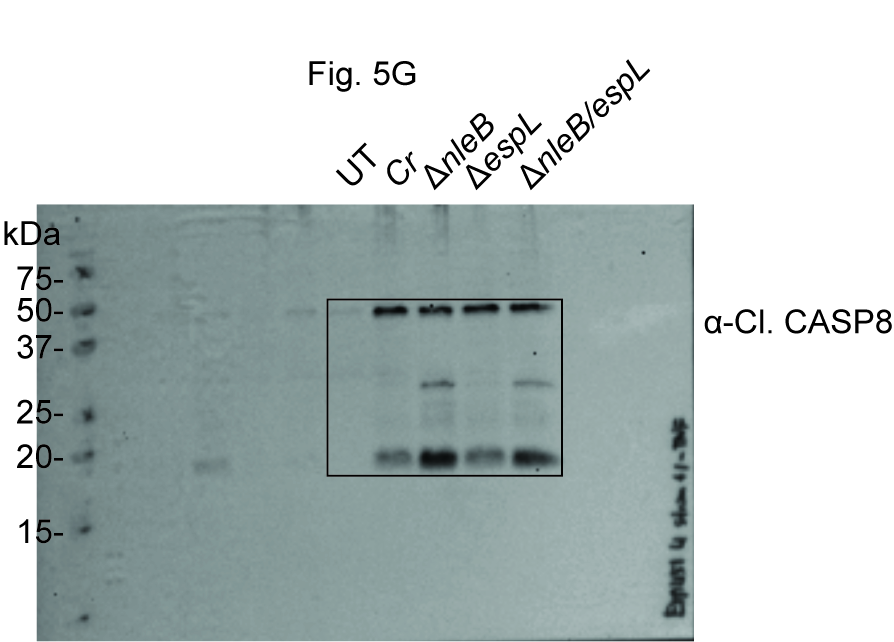

Supplement: Supplementary file 7 — Source data Fig. 5 [file 44318_2025_412_MOESM7_ESM.zip › Figure 5/5G/Fig5G Cl Casp8.tif]

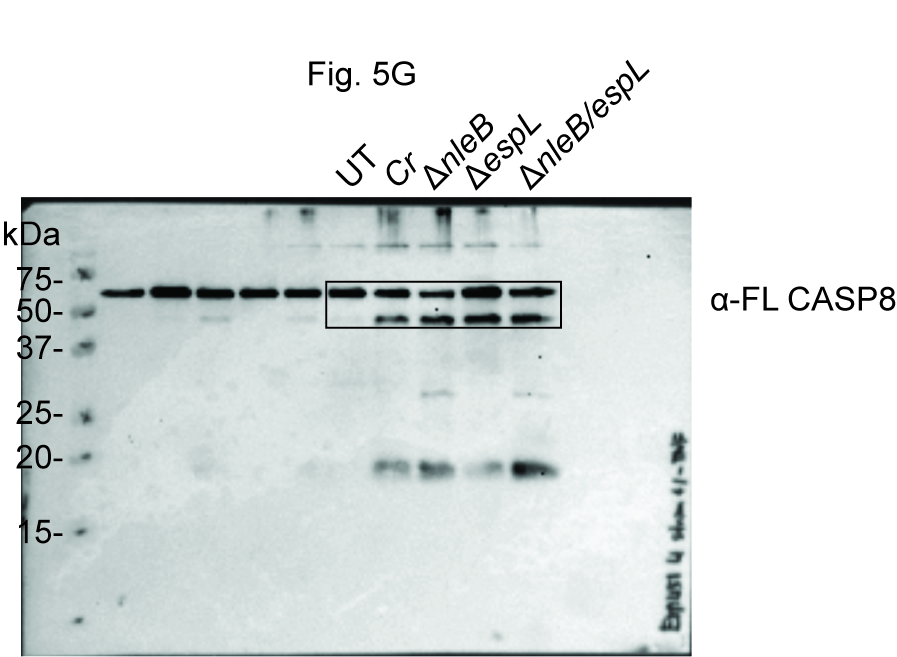

Supplement: Supplementary file 7 — Source data Fig. 5 [file 44318_2025_412_MOESM7_ESM.zip › Figure 5/5G/Fig5G FL Casp8.tif]

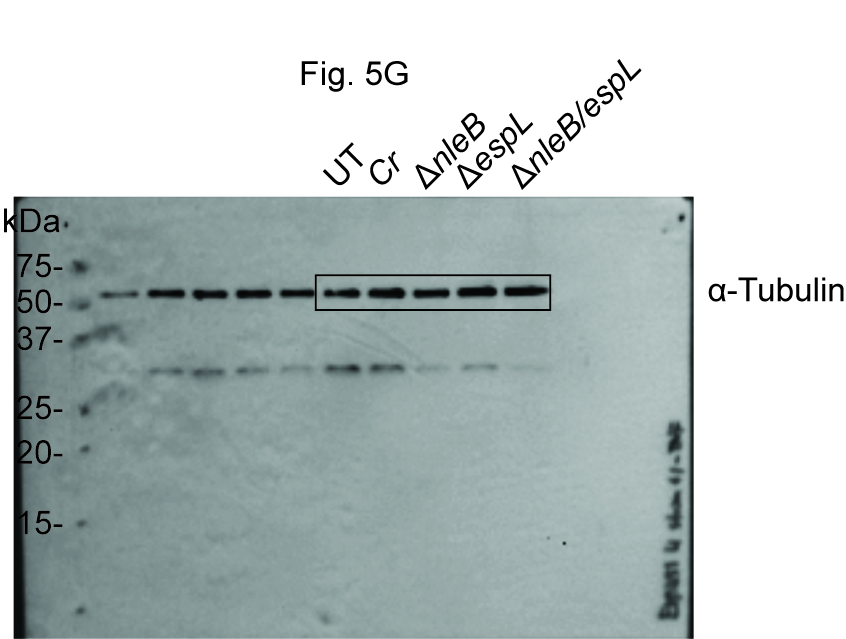

Supplement: Supplementary file 7 — Source data Fig. 5 [file 44318_2025_412_MOESM7_ESM.zip › Figure 5/5G/Fig5G Tubulin.tif]

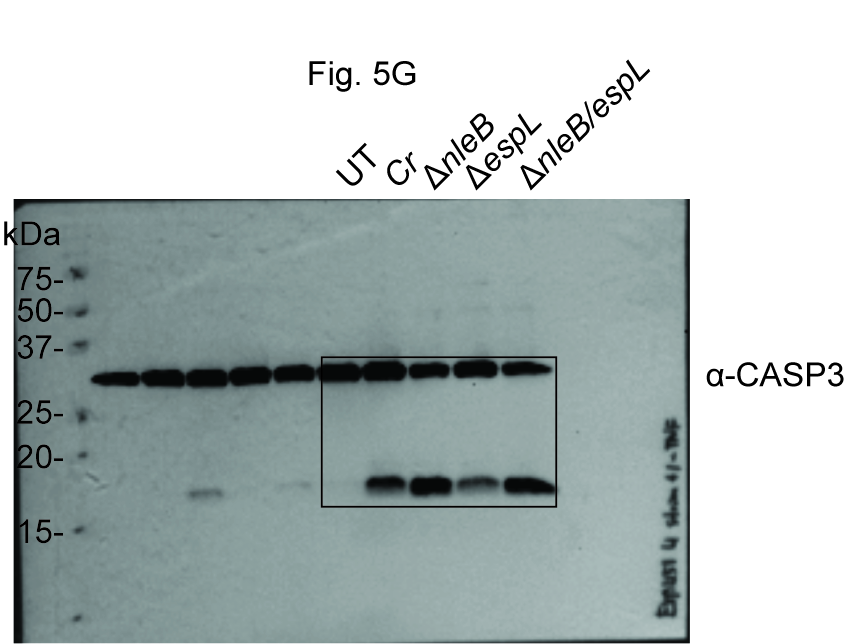

Supplement: Supplementary file 7 — Source data Fig. 5 [file 44318_2025_412_MOESM7_ESM.zip › Figure 5/5G/Fig5G Casp3.tif]

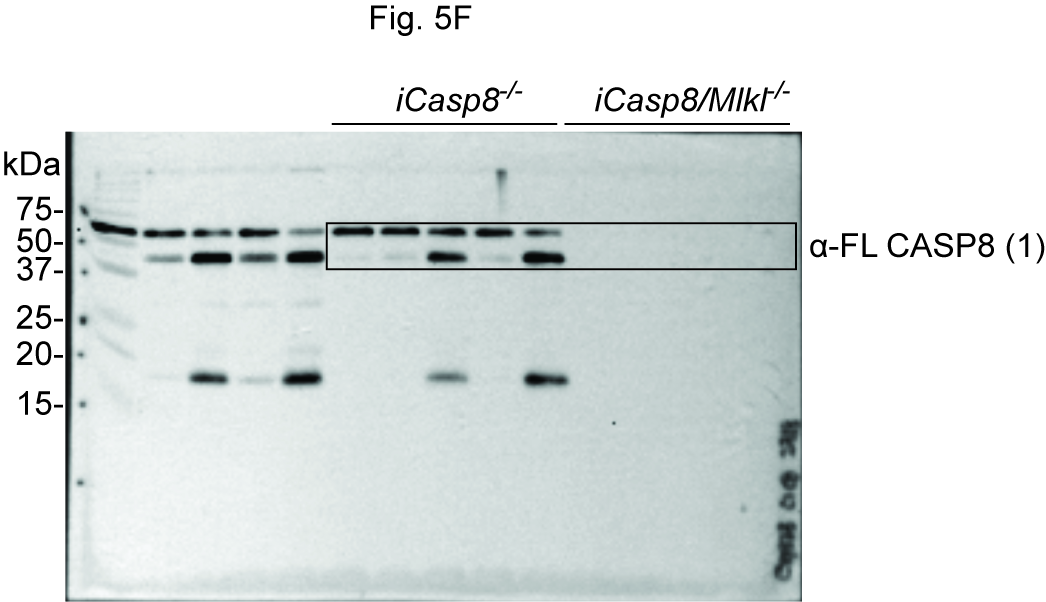

Supplement: Supplementary file 7 — Source data Fig. 5 [file 44318_2025_412_MOESM7_ESM.zip › Figure 5/5F/Fig5F FL Casp8.tif]

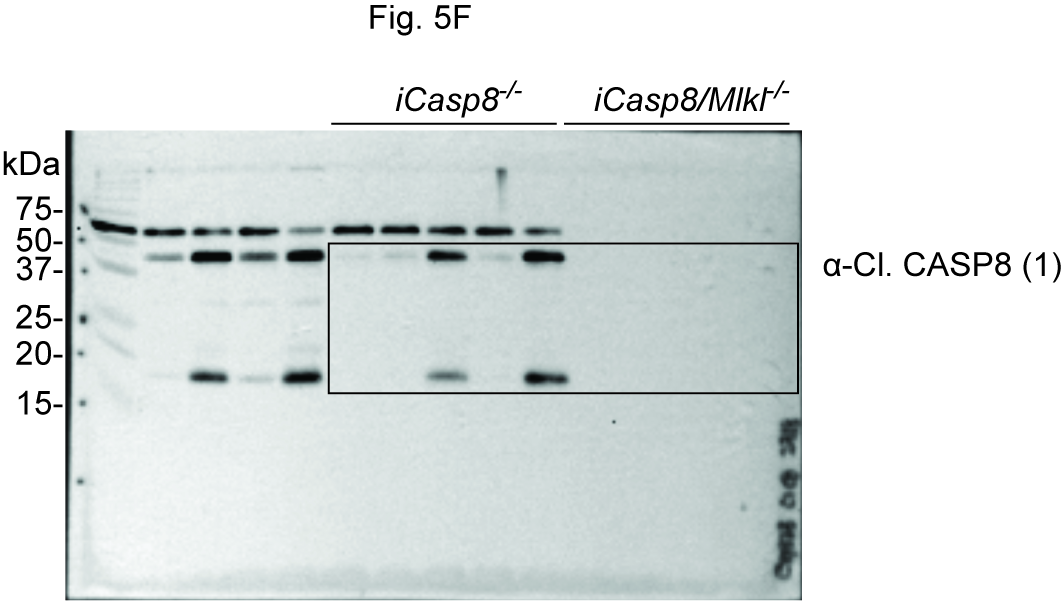

Supplement: Supplementary file 7 — Source data Fig. 5 [file 44318_2025_412_MOESM7_ESM.zip › Figure 5/5F/Fig5F Cl Casp8.tif]

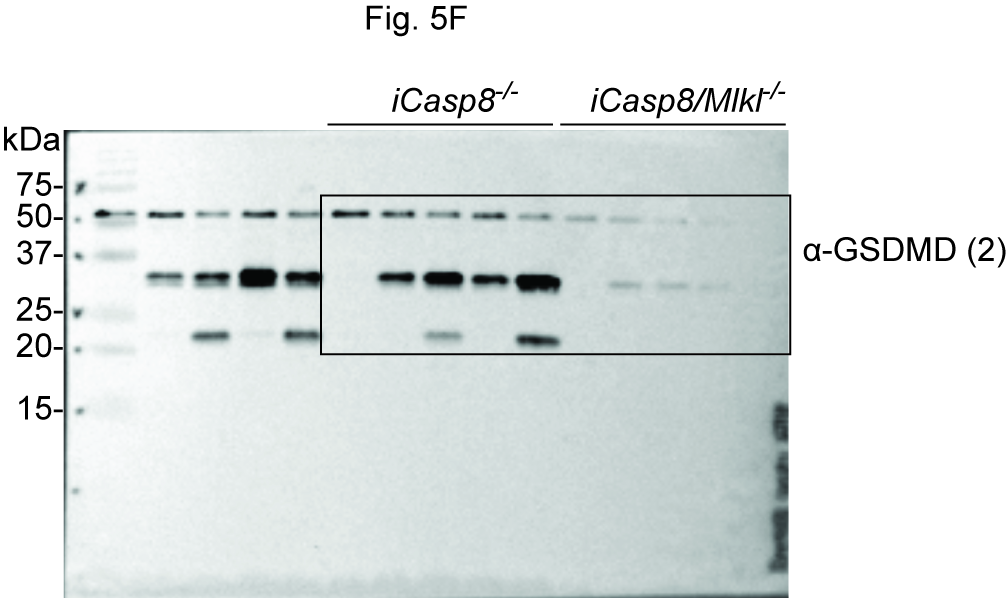

Supplement: Supplementary file 7 — Source data Fig. 5 [file 44318_2025_412_MOESM7_ESM.zip › Figure 5/5F/Fig5F GSDMD.tif]

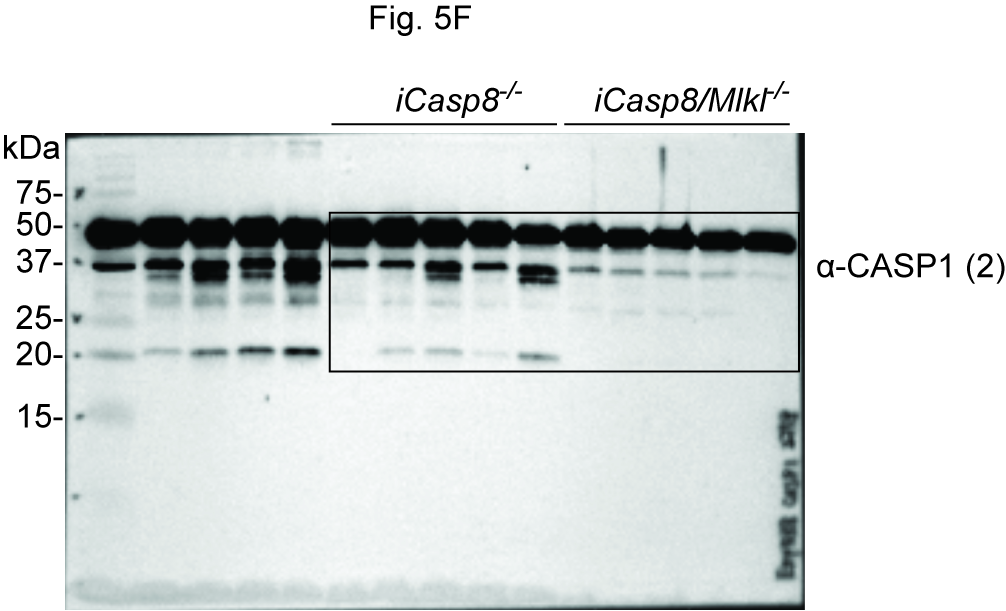

Supplement: Supplementary file 7 — Source data Fig. 5 [file 44318_2025_412_MOESM7_ESM.zip › Figure 5/5F/Fig5F Casp1 long exposure.tif]

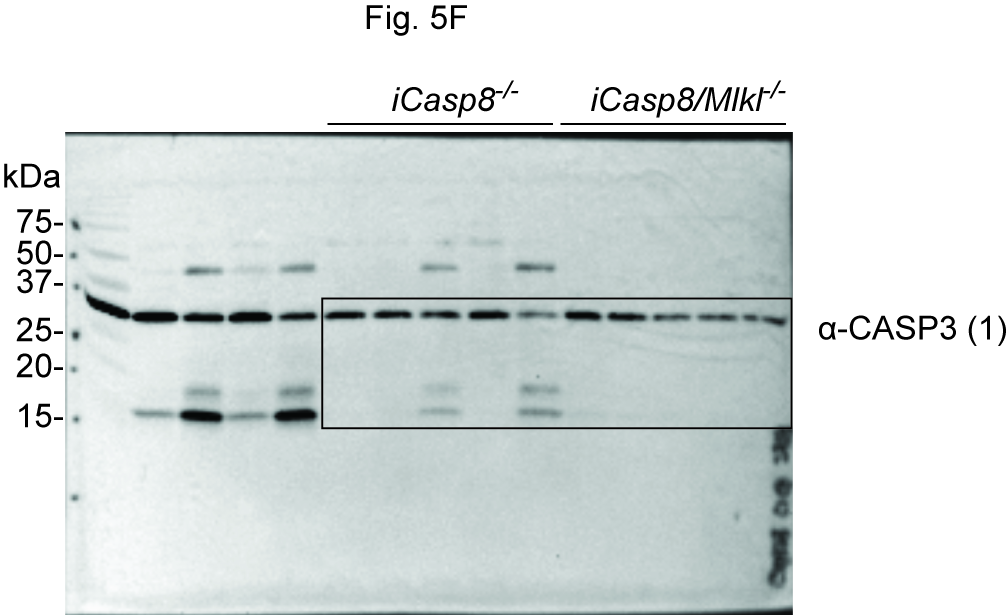

Supplement: Supplementary file 7 — Source data Fig. 5 [file 44318_2025_412_MOESM7_ESM.zip › Figure 5/5F/Fig5F Casp3.tif]

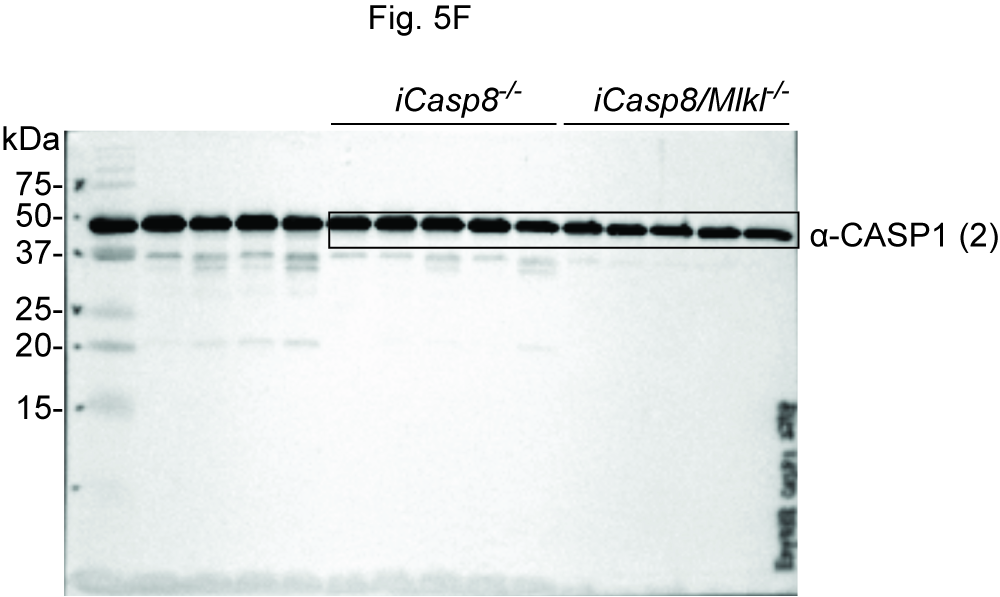

Supplement: Supplementary file 7 — Source data Fig. 5 [file 44318_2025_412_MOESM7_ESM.zip › Figure 5/5F/Fig5F Casp1.tif]

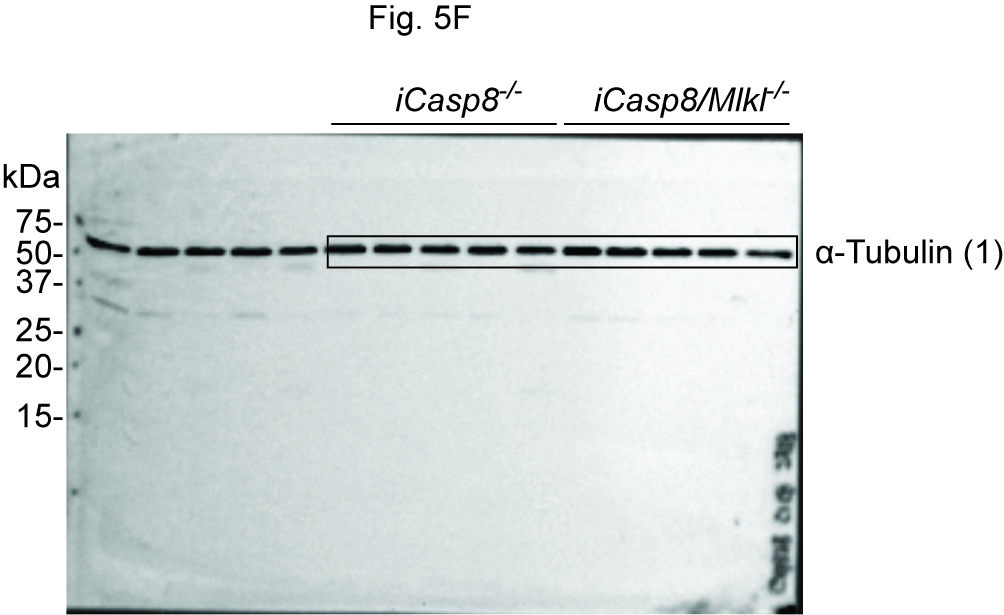

Supplement: Supplementary file 7 — Source data Fig. 5 [file 44318_2025_412_MOESM7_ESM.zip › Figure 5/5F/Fig5F Tubulin 1.tif]

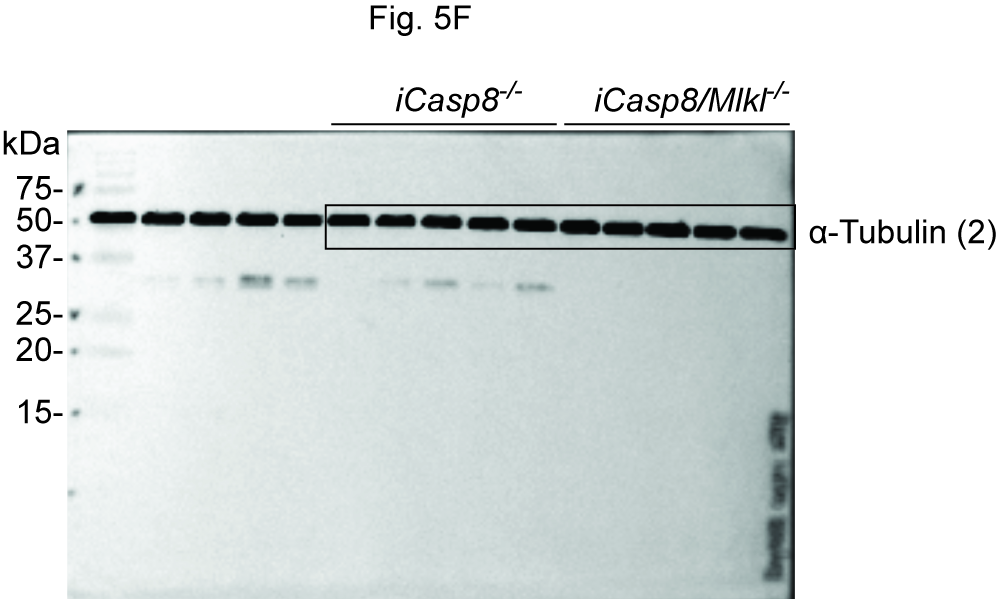

Supplement: Supplementary file 7 — Source data Fig. 5 [file 44318_2025_412_MOESM7_ESM.zip › Figure 5/5F/Fig5F Tubulin 2.tif]

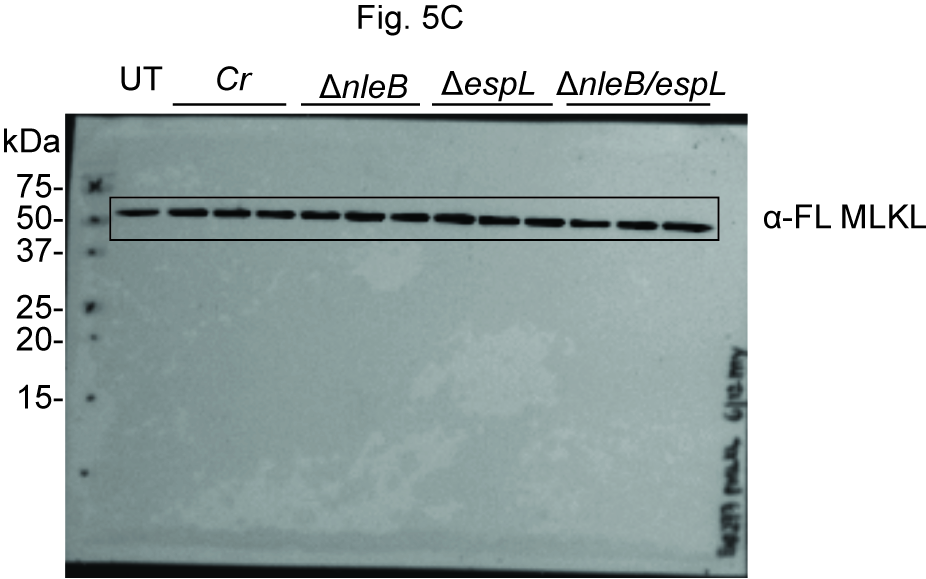

Supplement: Supplementary file 7 — Source data Fig. 5 [file 44318_2025_412_MOESM7_ESM.zip › Figure 5/5C/Fig5C MLKL.tif]

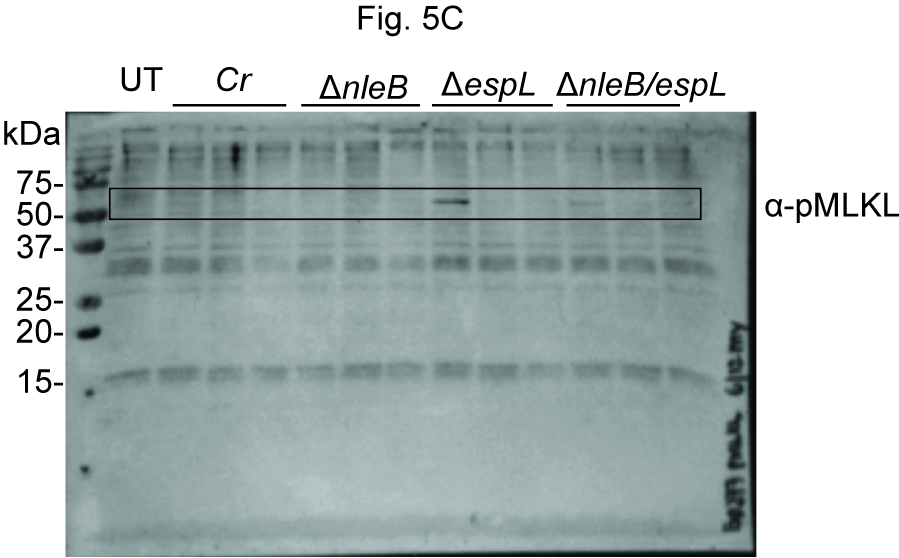

Supplement: Supplementary file 7 — Source data Fig. 5 [file 44318_2025_412_MOESM7_ESM.zip › Figure 5/5C/Fig5C pMLKL.tif]

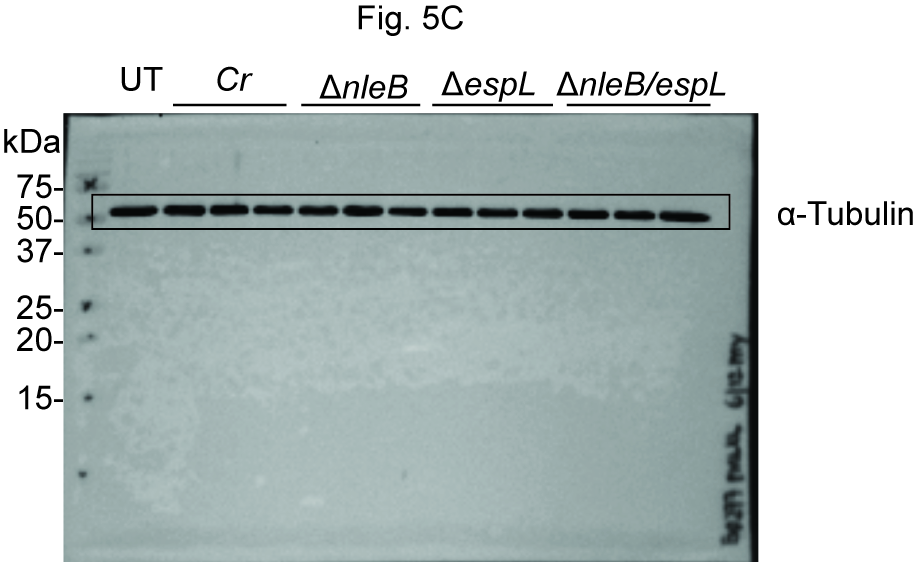

Supplement: Supplementary file 7 — Source data Fig. 5 [file 44318_2025_412_MOESM7_ESM.zip › Figure 5/5C/Fig5C Tubulin.tif]

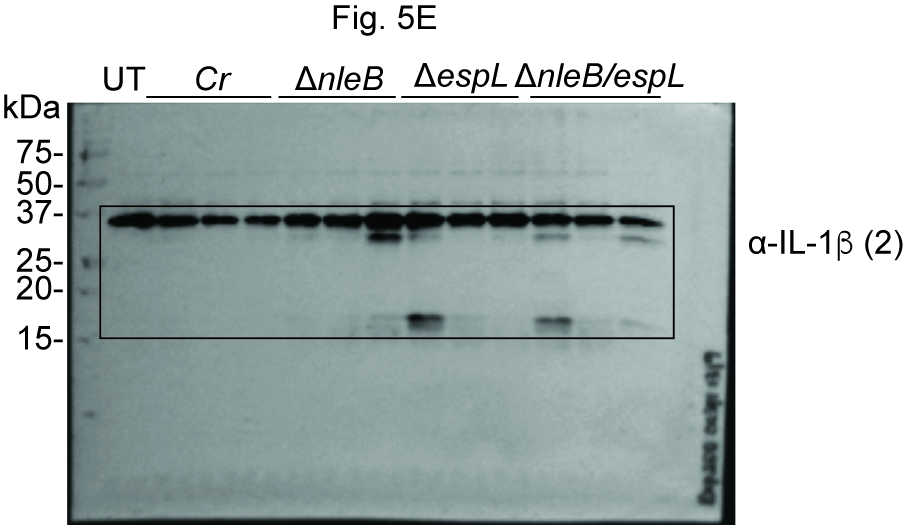

Supplement: Supplementary file 7 — Source data Fig. 5 [file 44318_2025_412_MOESM7_ESM.zip › Figure 5/5E/Fig5E IL-1b.tif]

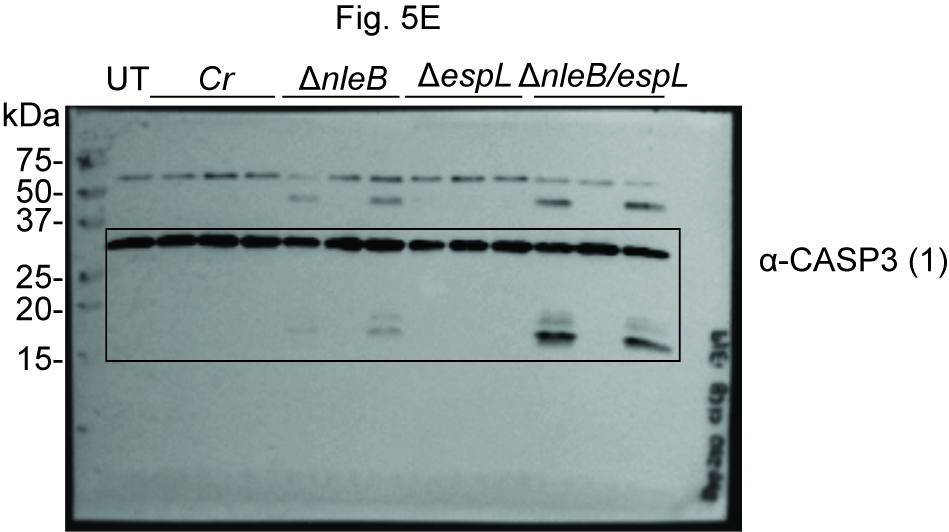

Supplement: Supplementary file 7 — Source data Fig. 5 [file 44318_2025_412_MOESM7_ESM.zip › Figure 5/5E/Fig5E Casp3.tif]

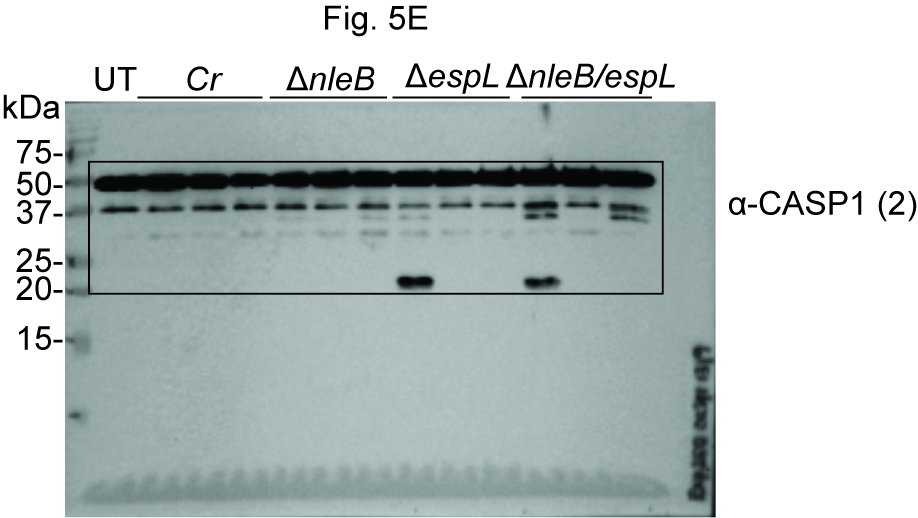

Supplement: Supplementary file 7 — Source data Fig. 5 [file 44318_2025_412_MOESM7_ESM.zip › Figure 5/5E/Fig5E Casp1.tif]

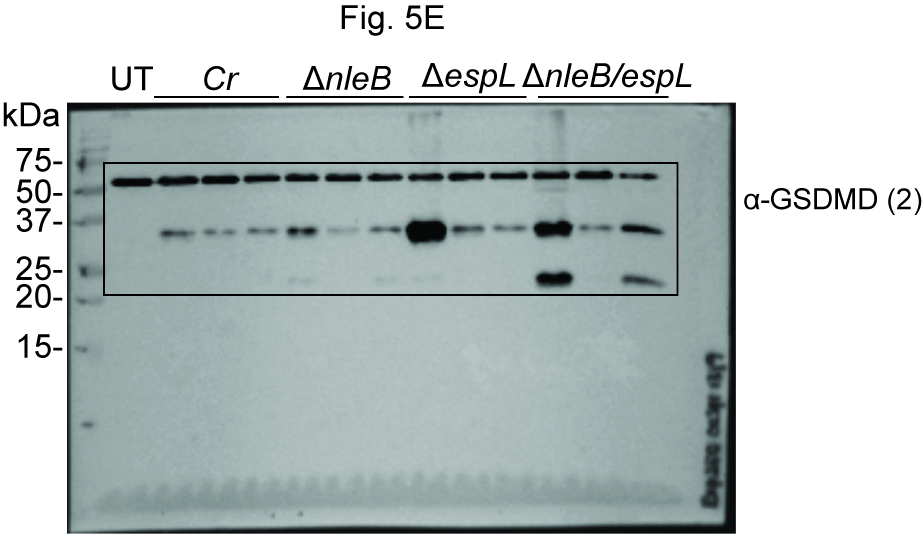

Supplement: Supplementary file 7 — Source data Fig. 5 [file 44318_2025_412_MOESM7_ESM.zip › Figure 5/5E/Fig5E GSDMD.tif]

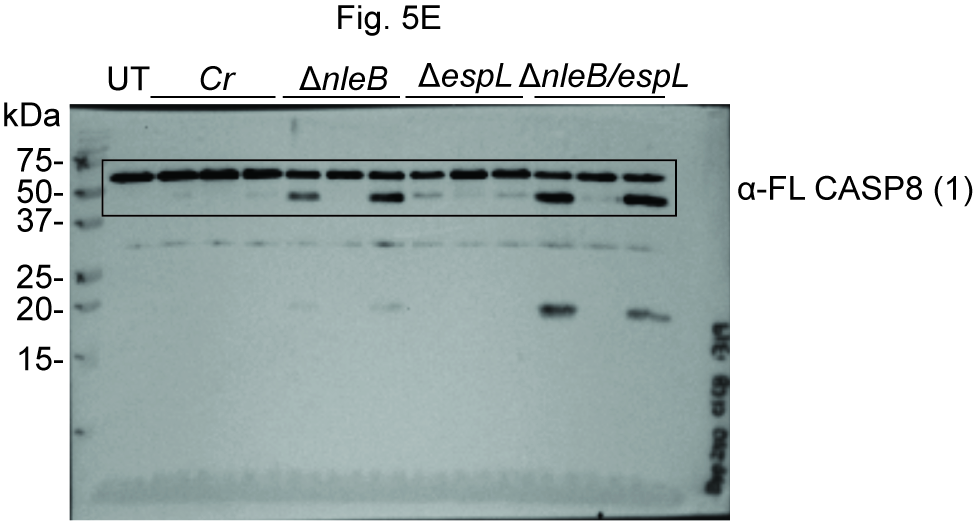

Supplement: Supplementary file 7 — Source data Fig. 5 [file 44318_2025_412_MOESM7_ESM.zip › Figure 5/5E/Fig5E FL Casp8.tif]

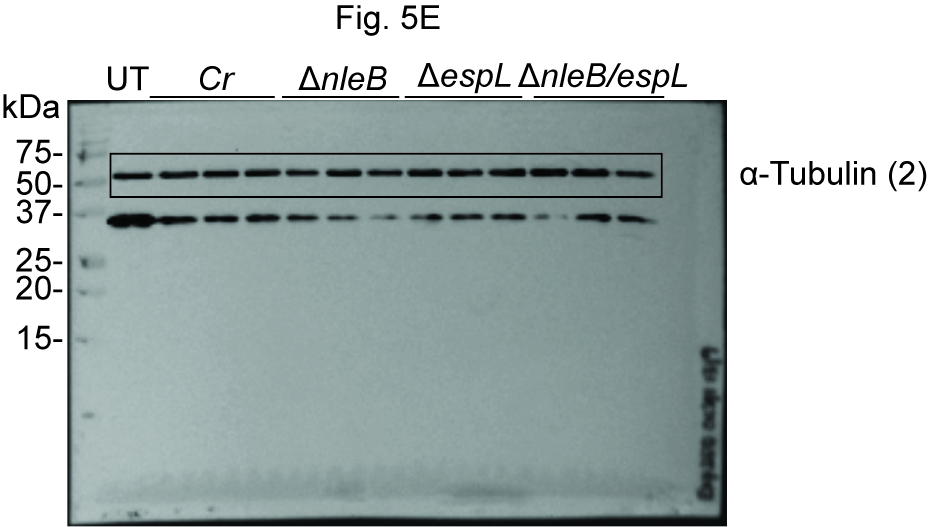

Supplement: Supplementary file 7 — Source data Fig. 5 [file 44318_2025_412_MOESM7_ESM.zip › Figure 5/5E/Fig5E Tubulin 2.tif]

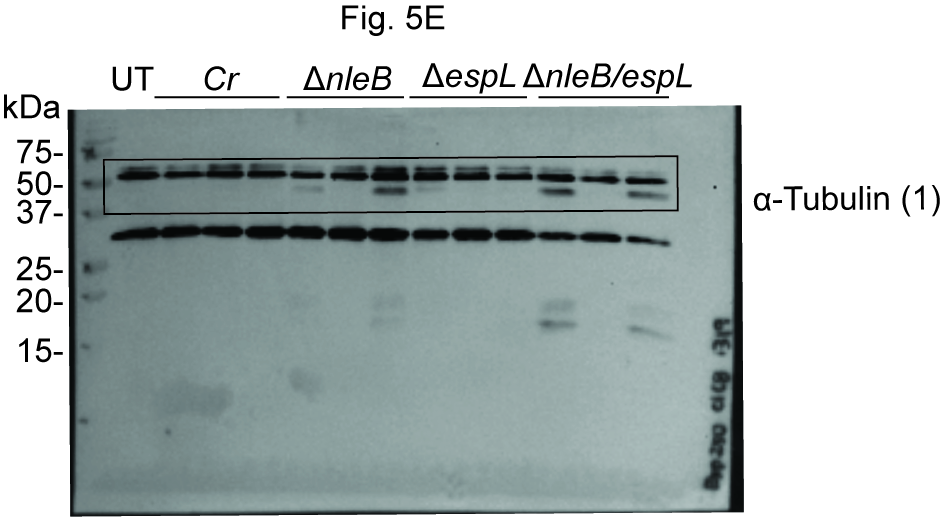

Supplement: Supplementary file 7 — Source data Fig. 5 [file 44318_2025_412_MOESM7_ESM.zip › Figure 5/5E/Fig5E Tubulin 1.tif]

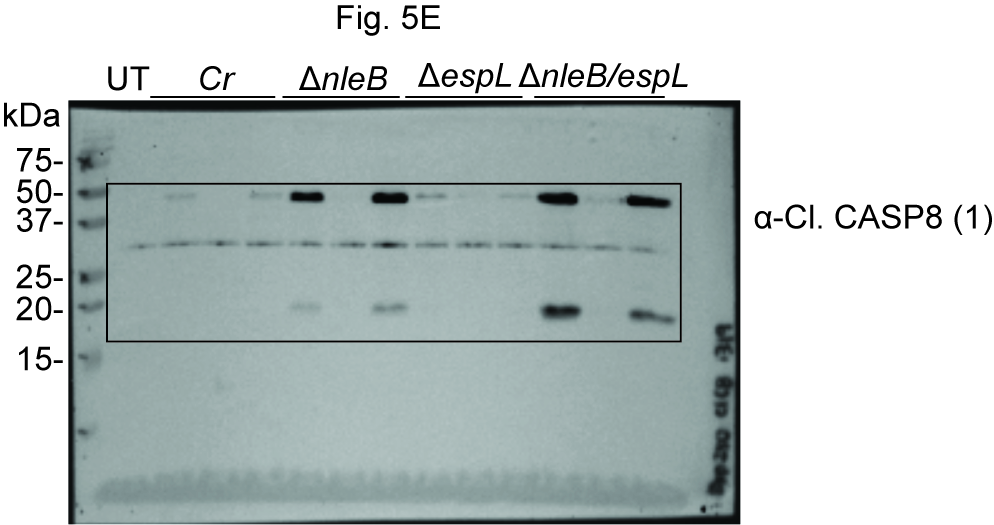

Supplement: Supplementary file 7 — Source data Fig. 5 [file 44318_2025_412_MOESM7_ESM.zip › Figure 5/5E/Fig5E Cl Casp8.tif]

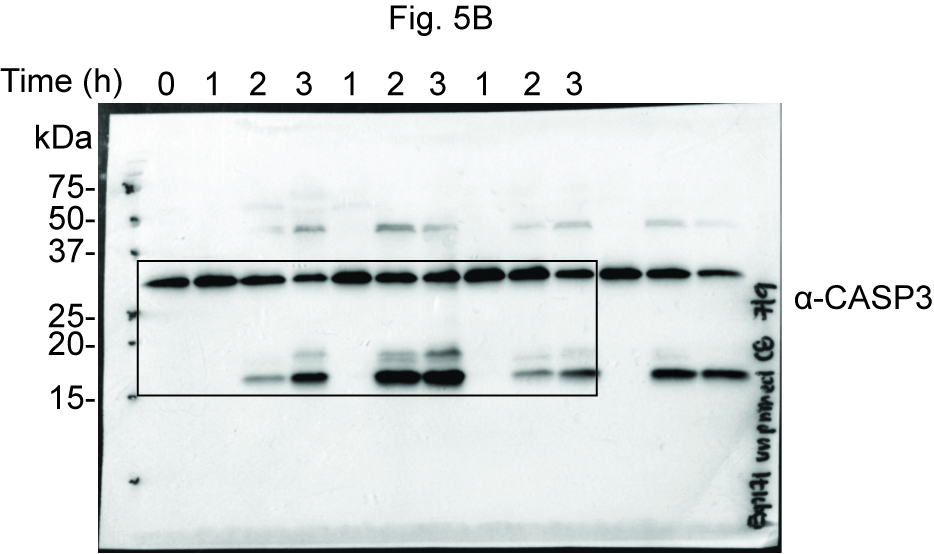

Supplement: Supplementary file 7 — Source data Fig. 5 [file 44318_2025_412_MOESM7_ESM.zip › Figure 5/5B/Fig5B Casp3.tif]

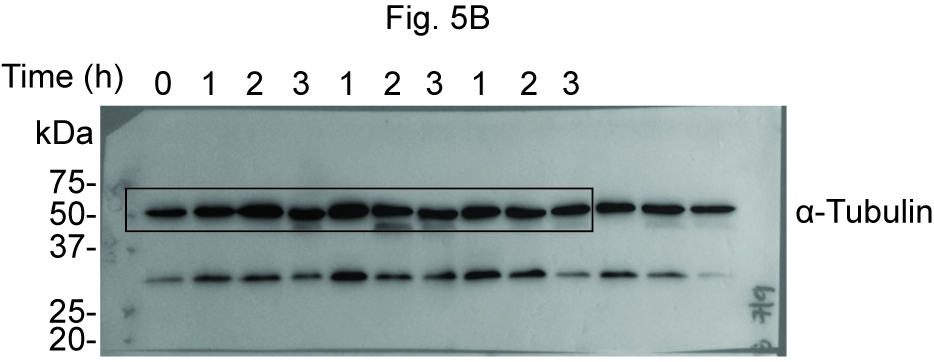

Supplement: Supplementary file 7 — Source data Fig. 5 [file 44318_2025_412_MOESM7_ESM.zip › Figure 5/5B/Fig5B Tubulin.tif]

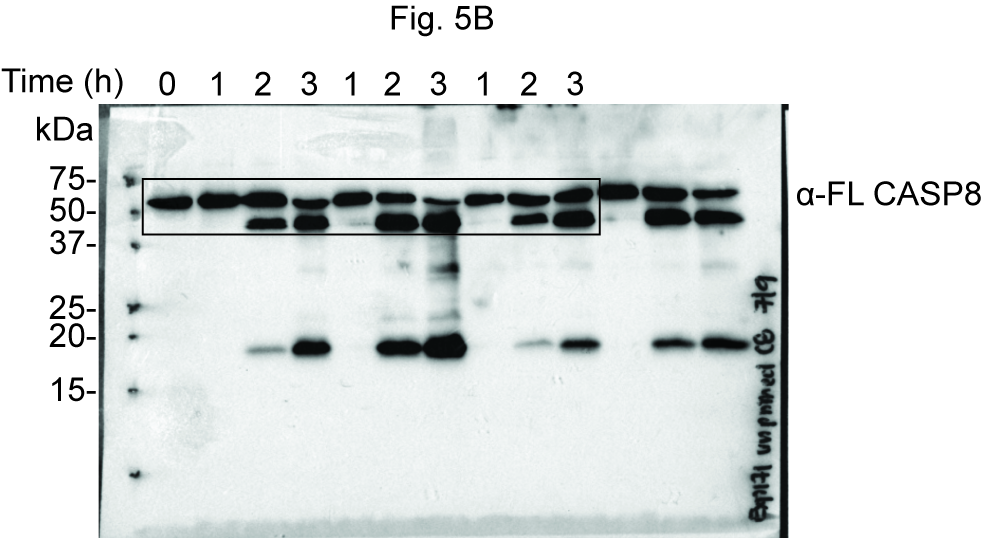

Supplement: Supplementary file 7 — Source data Fig. 5 [file 44318_2025_412_MOESM7_ESM.zip › Figure 5/5B/Fig5B FL Casp8.tif]

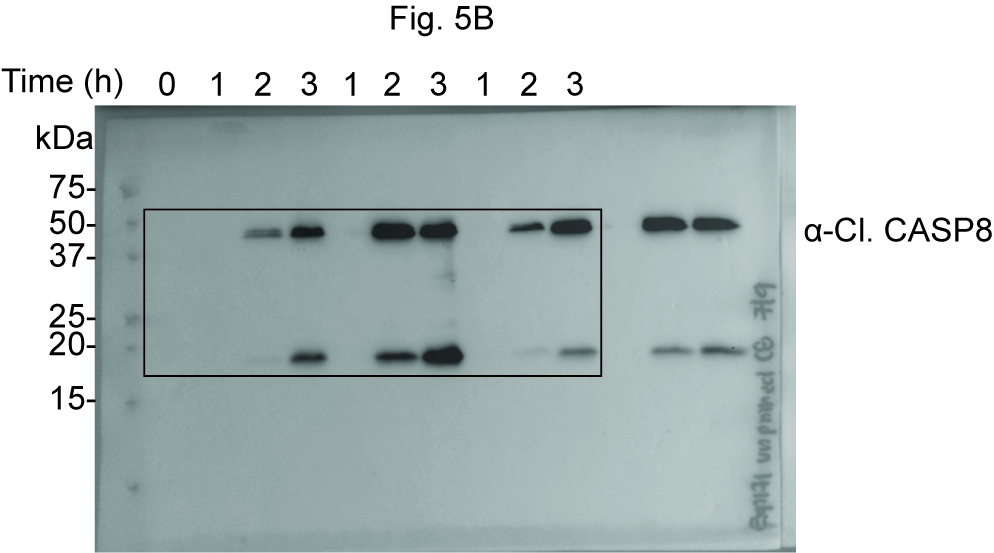

Supplement: Supplementary file 7 — Source data Fig. 5 [file 44318_2025_412_MOESM7_ESM.zip › Figure 5/5B/Fig5B Cl Casp8.tif]
