# Supplementary figures and images for: A bacterial network of T3SS effectors counteracts host pro-inflammatory responses and cell death to promote infection (part 2 of 2)
Source: EMBO J. 2025 Mar 24;44(9):2424–45. doi: 10.1038/s44318-025-00412-5 (PMC12048508; doi:10.1038/s44318-025-00412-5)

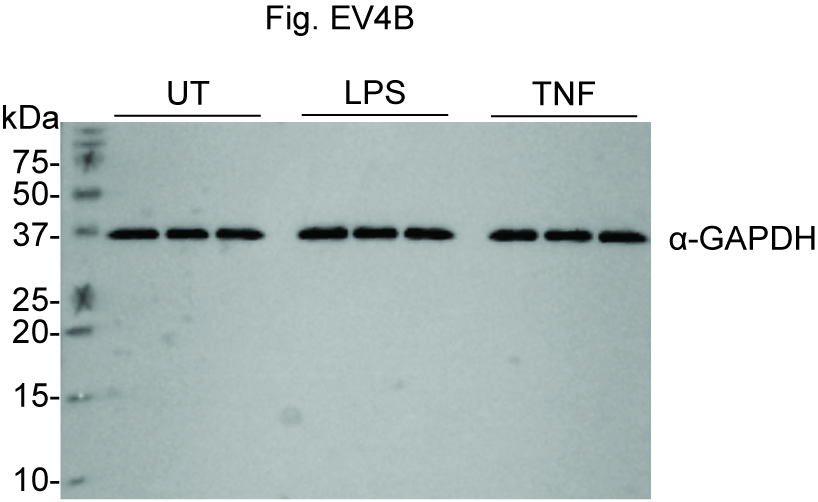

Supplement: Supplementary file 8 — EV Figure Source Data [file 44318_2025_412_MOESM8_ESM.zip › EMBOJ-2024-118621-SourceData_ExpandedViewAndAppendix/Expanded view 4/4B/EV4B GAPDH.tif]

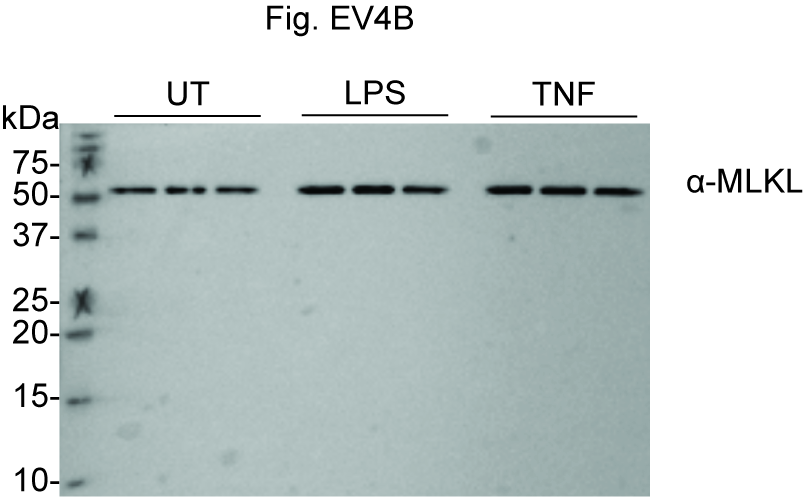

Supplement: Supplementary file 8 — EV Figure Source Data [file 44318_2025_412_MOESM8_ESM.zip › EMBOJ-2024-118621-SourceData_ExpandedViewAndAppendix/Expanded view 4/4B/EV4B MLKL.tif]

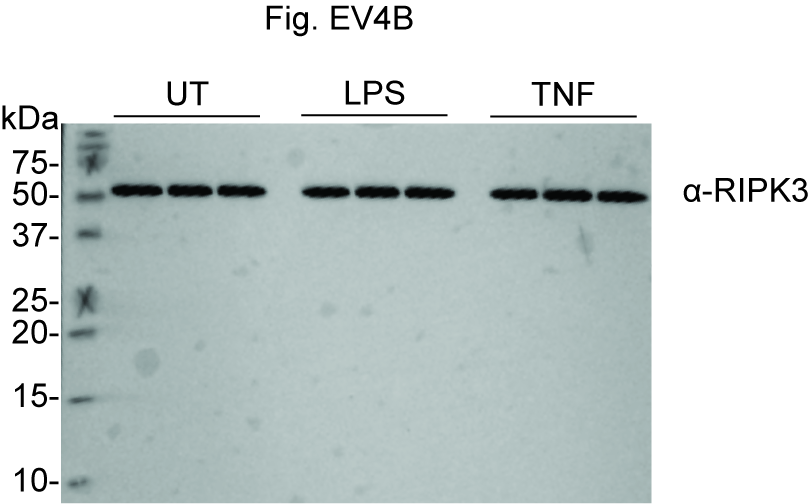

Supplement: Supplementary file 8 — EV Figure Source Data [file 44318_2025_412_MOESM8_ESM.zip › EMBOJ-2024-118621-SourceData_ExpandedViewAndAppendix/Expanded view 4/4B/EV4B RIPK3.tif]

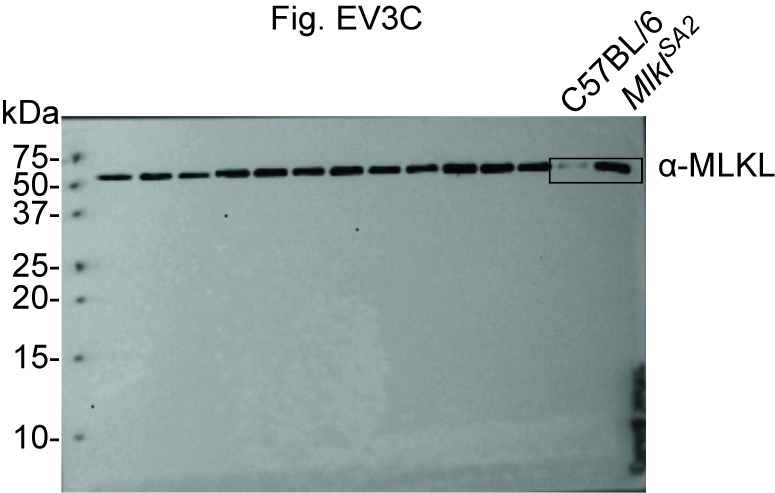

Supplement: Supplementary file 8 — EV Figure Source Data [file 44318_2025_412_MOESM8_ESM.zip › EMBOJ-2024-118621-SourceData_ExpandedViewAndAppendix/Expanded view 3/3C/EV3C MLKL.tif]

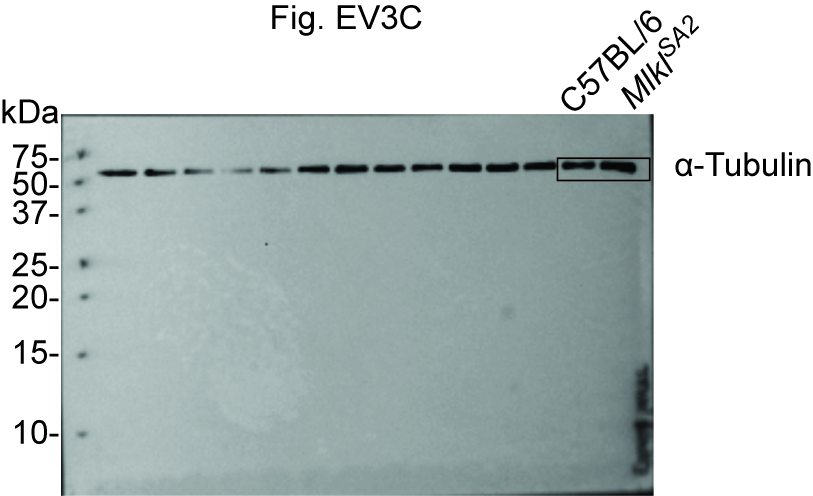

Supplement: Supplementary file 8 — EV Figure Source Data [file 44318_2025_412_MOESM8_ESM.zip › EMBOJ-2024-118621-SourceData_ExpandedViewAndAppendix/Expanded view 3/3C/EV3C Tubulin.tif]

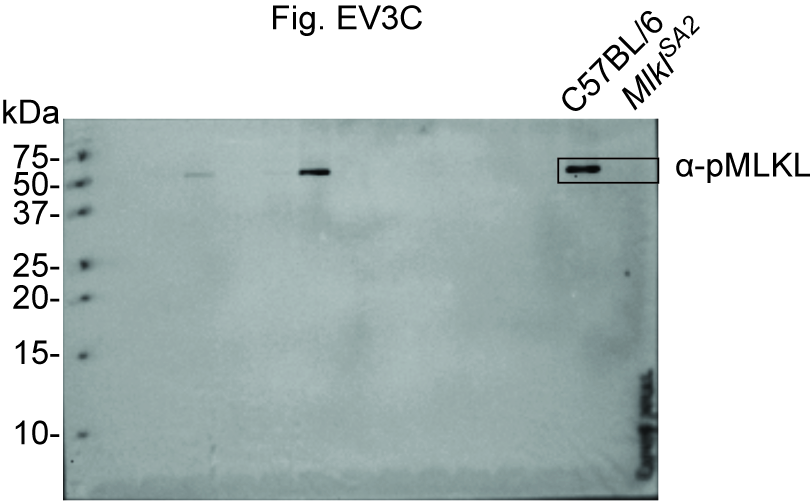

Supplement: Supplementary file 8 — EV Figure Source Data [file 44318_2025_412_MOESM8_ESM.zip › EMBOJ-2024-118621-SourceData_ExpandedViewAndAppendix/Expanded view 3/3C/EV3C pMLKL.tif]

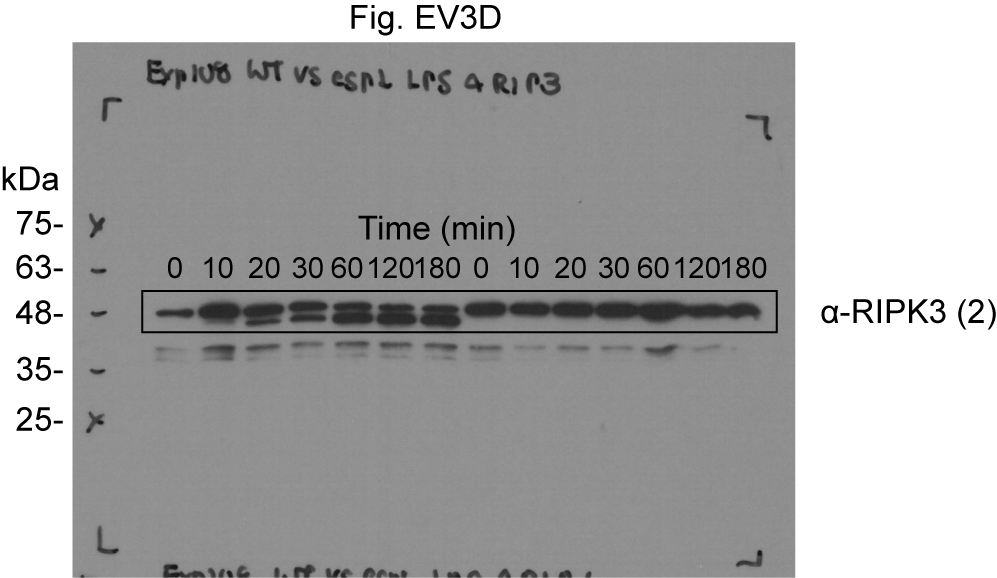

Supplement: Supplementary file 8 — EV Figure Source Data [file 44318_2025_412_MOESM8_ESM.zip › EMBOJ-2024-118621-SourceData_ExpandedViewAndAppendix/Expanded view 3/3D/EV3D RIPK3.tif]

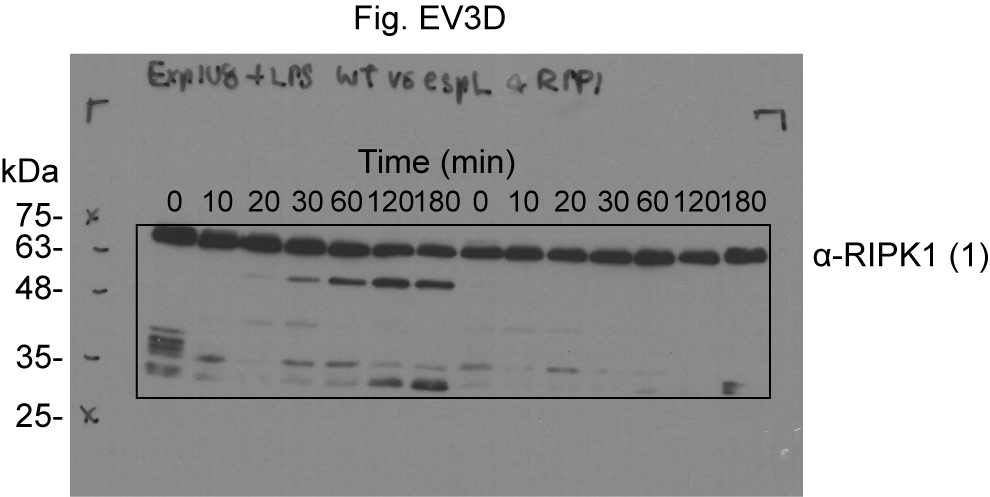

Supplement: Supplementary file 8 — EV Figure Source Data [file 44318_2025_412_MOESM8_ESM.zip › EMBOJ-2024-118621-SourceData_ExpandedViewAndAppendix/Expanded view 3/3D/EV3D RIPK1.tif]

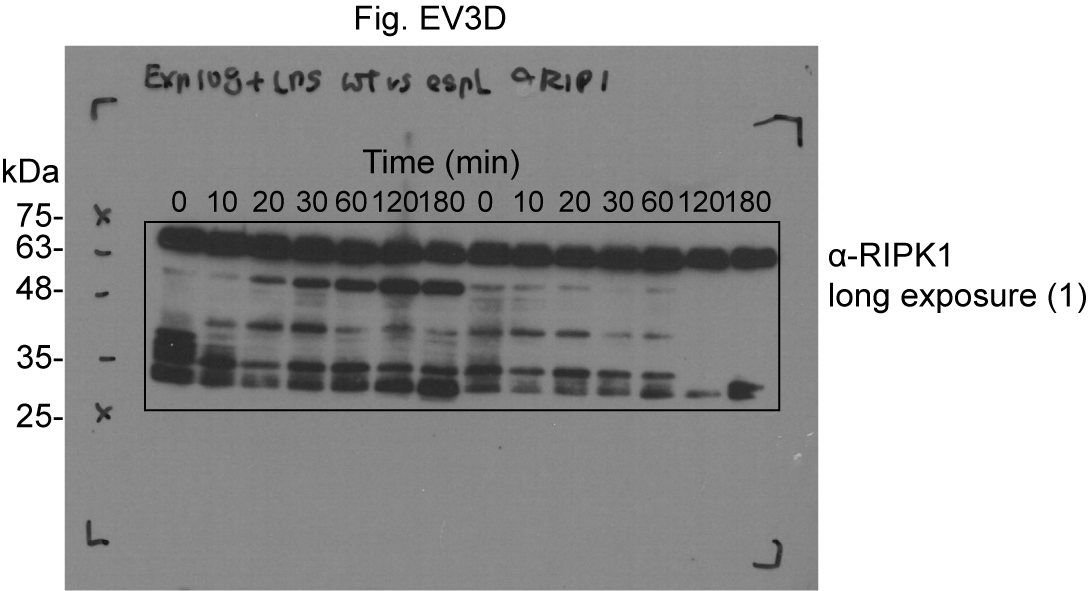

Supplement: Supplementary file 8 — EV Figure Source Data [file 44318_2025_412_MOESM8_ESM.zip › EMBOJ-2024-118621-SourceData_ExpandedViewAndAppendix/Expanded view 3/3D/EV3D RIPK1 long exposure.tif]

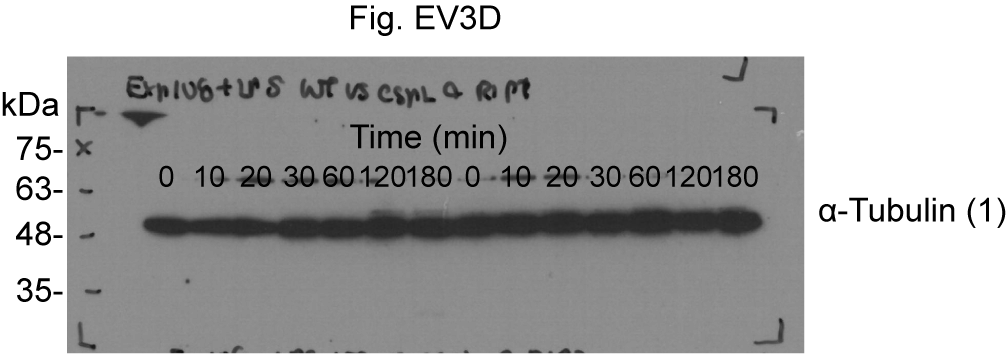

Supplement: Supplementary file 8 — EV Figure Source Data [file 44318_2025_412_MOESM8_ESM.zip › EMBOJ-2024-118621-SourceData_ExpandedViewAndAppendix/Expanded view 3/3D/EV3D Tubulin 1.tif]

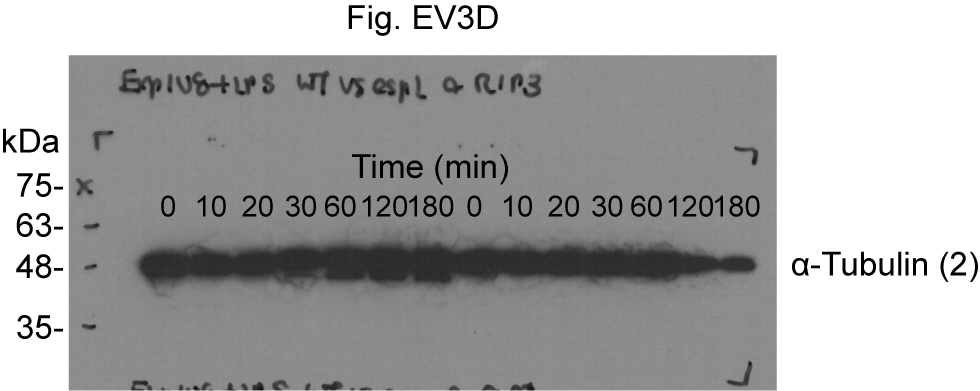

Supplement: Supplementary file 8 — EV Figure Source Data [file 44318_2025_412_MOESM8_ESM.zip › EMBOJ-2024-118621-SourceData_ExpandedViewAndAppendix/Expanded view 3/3D/EV3D Tubulin 2.tif]

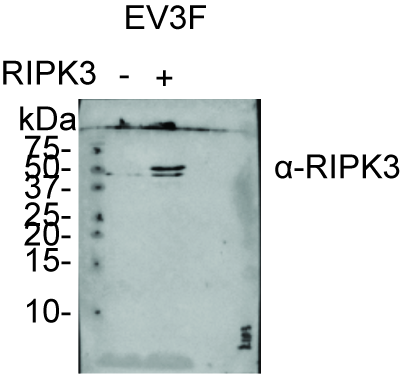

Supplement: Supplementary file 8 — EV Figure Source Data [file 44318_2025_412_MOESM8_ESM.zip › EMBOJ-2024-118621-SourceData_ExpandedViewAndAppendix/Expanded view 3/3F/EV3F RIPK3.tif]

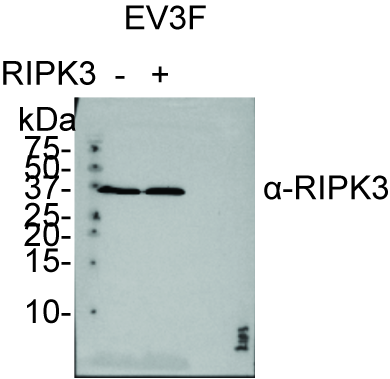

Supplement: Supplementary file 8 — EV Figure Source Data [file 44318_2025_412_MOESM8_ESM.zip › EMBOJ-2024-118621-SourceData_ExpandedViewAndAppendix/Expanded view 3/3F/EV3F GAPDH.tif]

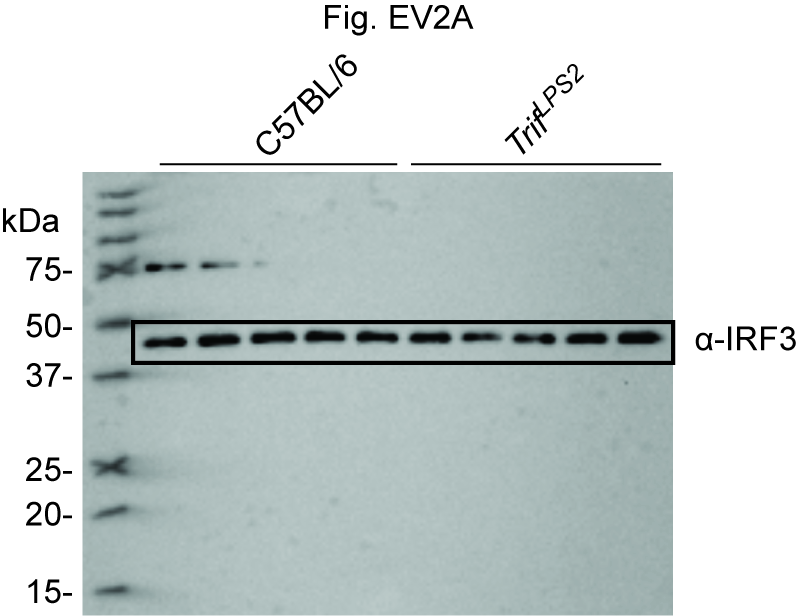

Supplement: Supplementary file 8 — EV Figure Source Data [file 44318_2025_412_MOESM8_ESM.zip › EMBOJ-2024-118621-SourceData_ExpandedViewAndAppendix/Expanded view 2/2A/EV2A IRF3.tif]

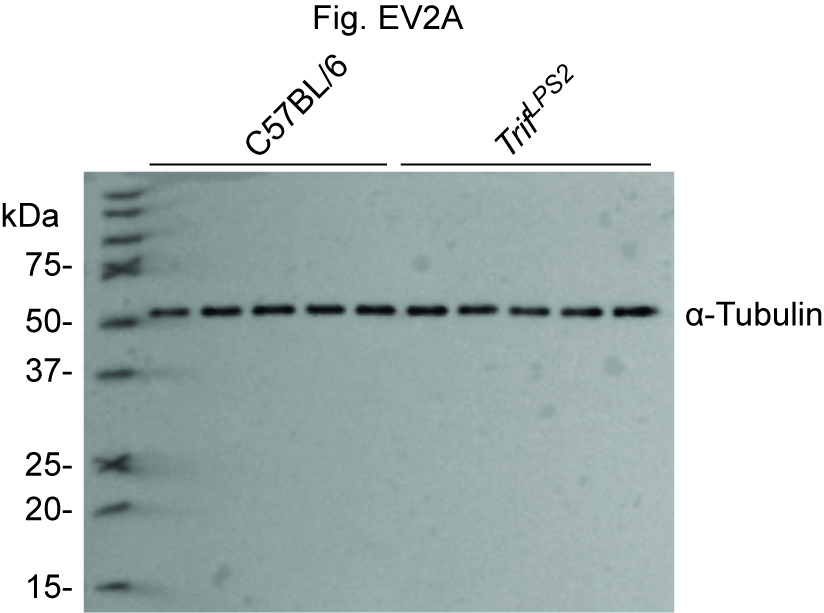

Supplement: Supplementary file 8 — EV Figure Source Data [file 44318_2025_412_MOESM8_ESM.zip › EMBOJ-2024-118621-SourceData_ExpandedViewAndAppendix/Expanded view 2/2A/EV2A Tubulin.tif]

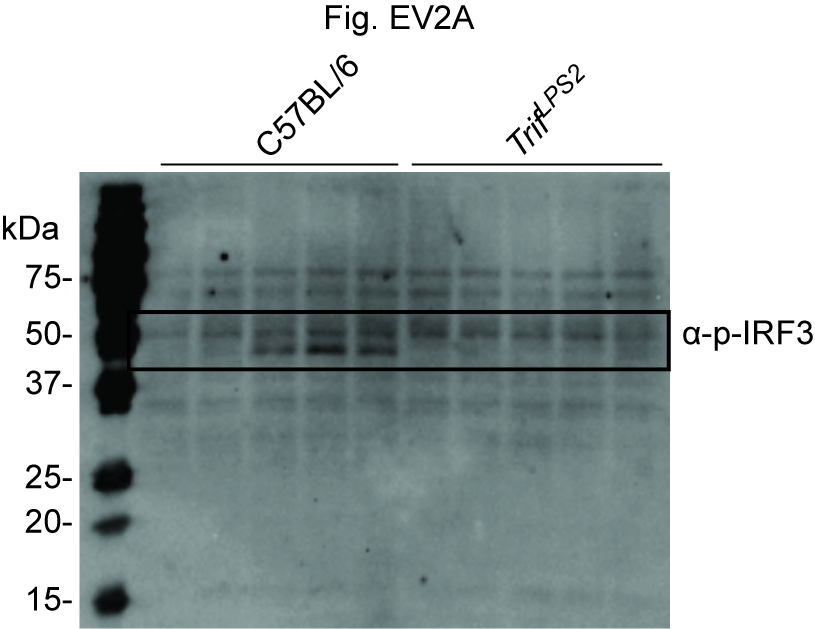

Supplement: Supplementary file 8 — EV Figure Source Data [file 44318_2025_412_MOESM8_ESM.zip › EMBOJ-2024-118621-SourceData_ExpandedViewAndAppendix/Expanded view 2/2A/EV2A p-IRF3.tif]

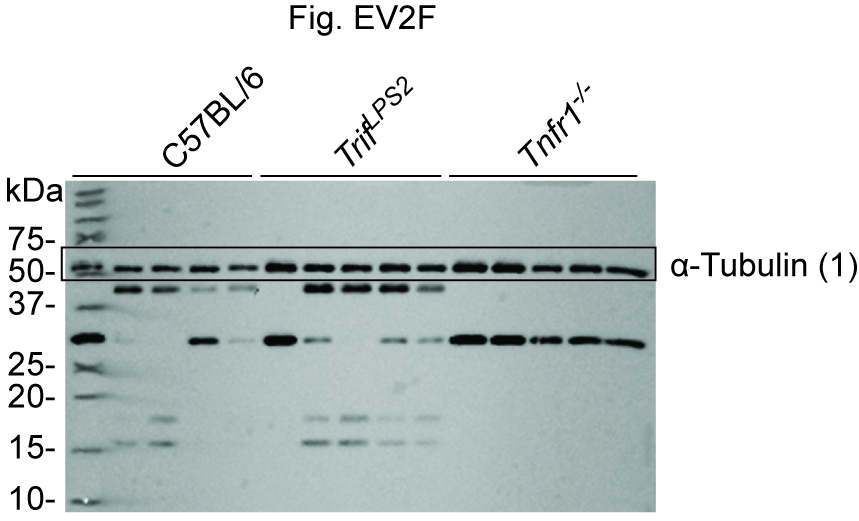

Supplement: Supplementary file 8 — EV Figure Source Data [file 44318_2025_412_MOESM8_ESM.zip › EMBOJ-2024-118621-SourceData_ExpandedViewAndAppendix/Expanded view 2/2F/EV2F Tubulin 1.tif]

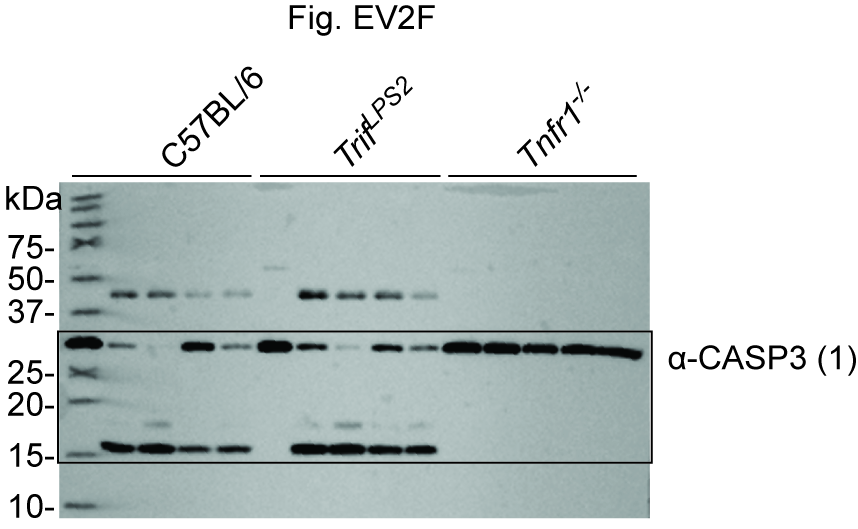

Supplement: Supplementary file 8 — EV Figure Source Data [file 44318_2025_412_MOESM8_ESM.zip › EMBOJ-2024-118621-SourceData_ExpandedViewAndAppendix/Expanded view 2/2F/EV2F Casp3.tif]

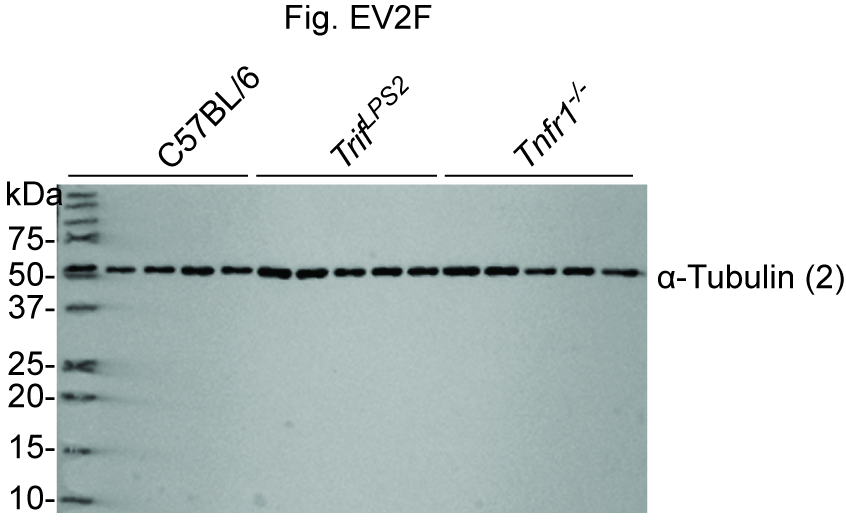

Supplement: Supplementary file 8 — EV Figure Source Data [file 44318_2025_412_MOESM8_ESM.zip › EMBOJ-2024-118621-SourceData_ExpandedViewAndAppendix/Expanded view 2/2F/EV2F Tubulin 2.tif]

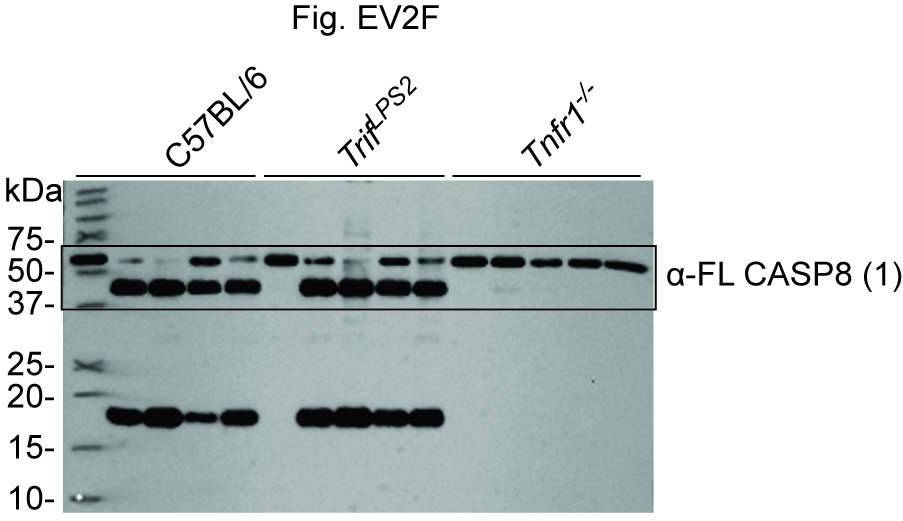

Supplement: Supplementary file 8 — EV Figure Source Data [file 44318_2025_412_MOESM8_ESM.zip › EMBOJ-2024-118621-SourceData_ExpandedViewAndAppendix/Expanded view 2/2F/EV2F FL Casp8.tif]

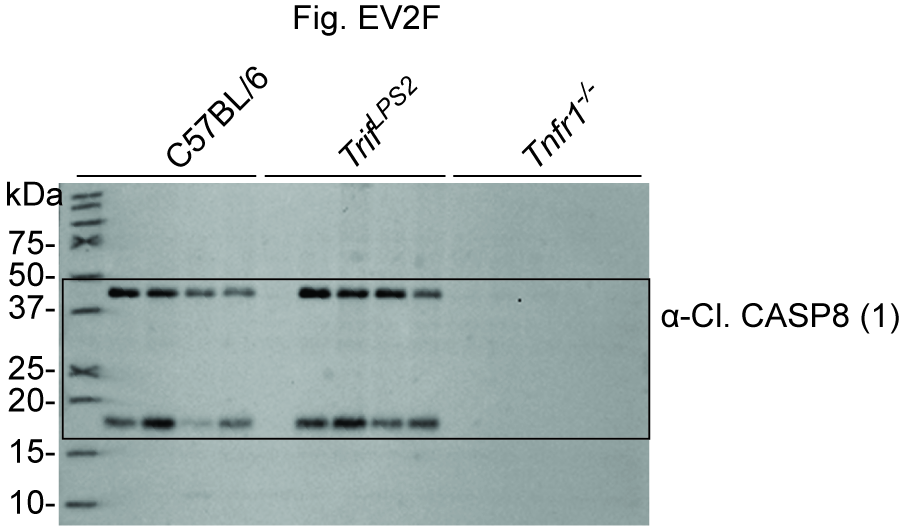

Supplement: Supplementary file 8 — EV Figure Source Data [file 44318_2025_412_MOESM8_ESM.zip › EMBOJ-2024-118621-SourceData_ExpandedViewAndAppendix/Expanded view 2/2F/EV2F Cl Casp8.tif]

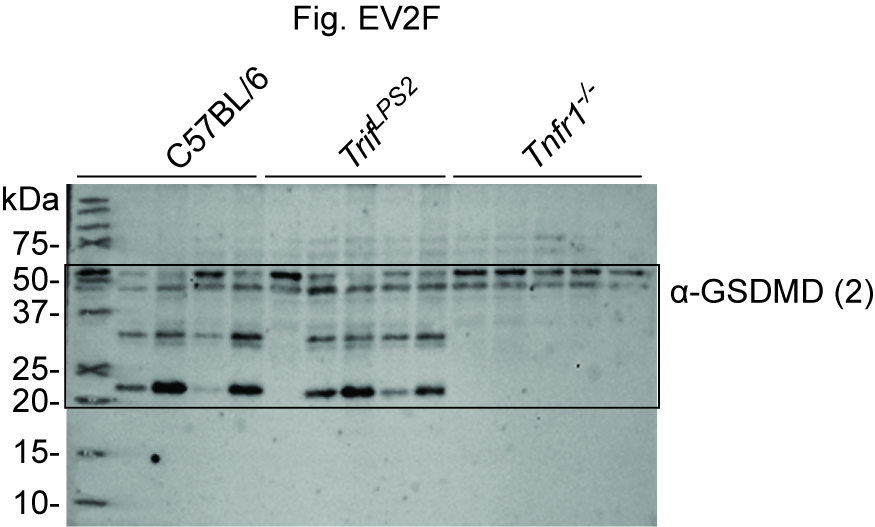

Supplement: Supplementary file 8 — EV Figure Source Data [file 44318_2025_412_MOESM8_ESM.zip › EMBOJ-2024-118621-SourceData_ExpandedViewAndAppendix/Expanded view 2/2F/EV2F GSDMD.tif]

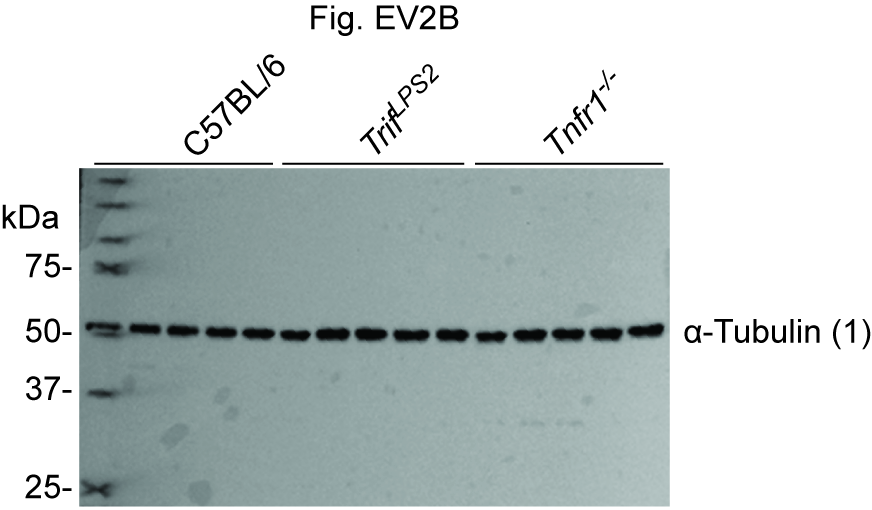

Supplement: Supplementary file 8 — EV Figure Source Data [file 44318_2025_412_MOESM8_ESM.zip › EMBOJ-2024-118621-SourceData_ExpandedViewAndAppendix/Expanded view 2/2B/EV2B Tubulin 1.tif]

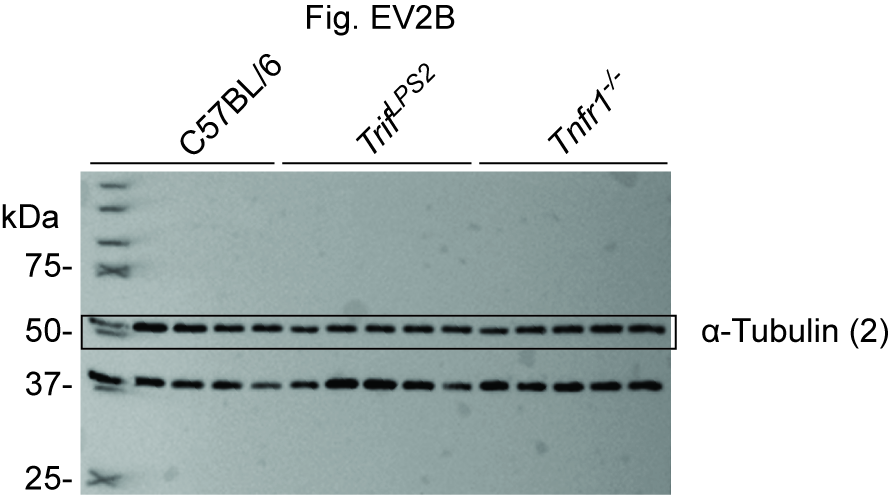

Supplement: Supplementary file 8 — EV Figure Source Data [file 44318_2025_412_MOESM8_ESM.zip › EMBOJ-2024-118621-SourceData_ExpandedViewAndAppendix/Expanded view 2/2B/EV2B Tubulin 2.tif]

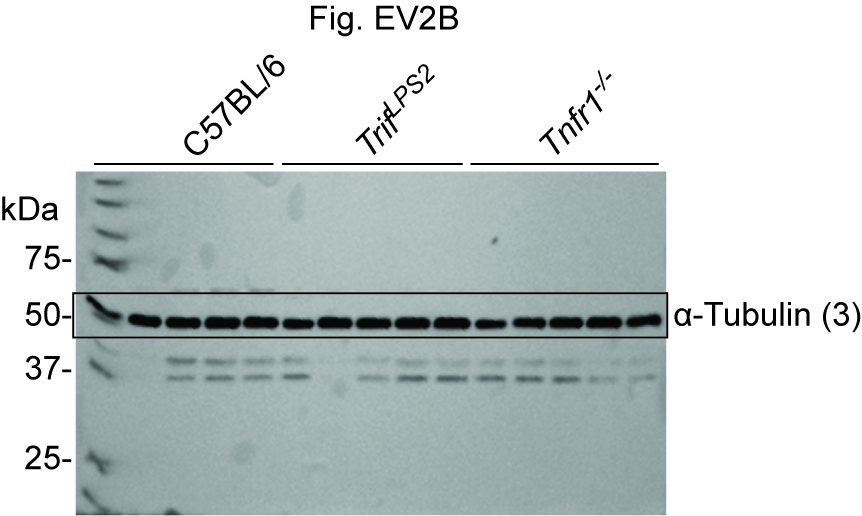

Supplement: Supplementary file 8 — EV Figure Source Data [file 44318_2025_412_MOESM8_ESM.zip › EMBOJ-2024-118621-SourceData_ExpandedViewAndAppendix/Expanded view 2/2B/EV2B Tubulin 3.tif]

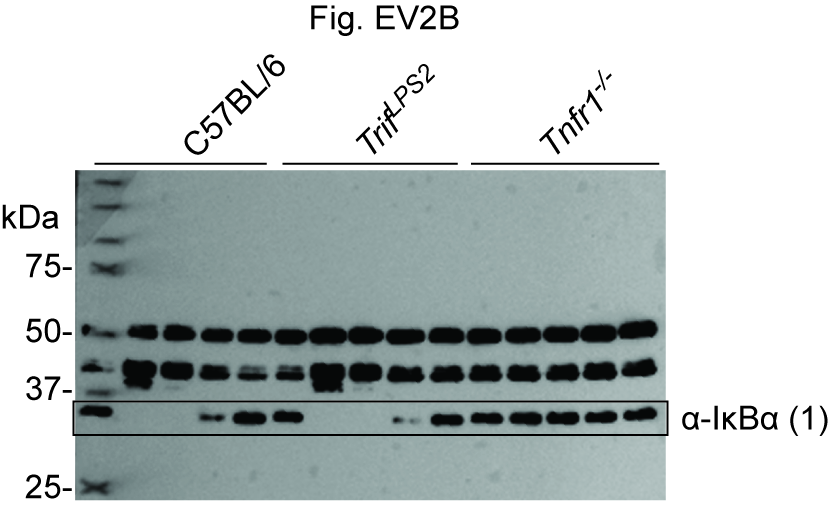

Supplement: Supplementary file 8 — EV Figure Source Data [file 44318_2025_412_MOESM8_ESM.zip › EMBOJ-2024-118621-SourceData_ExpandedViewAndAppendix/Expanded view 2/2B/EV2B IkBa.tif]

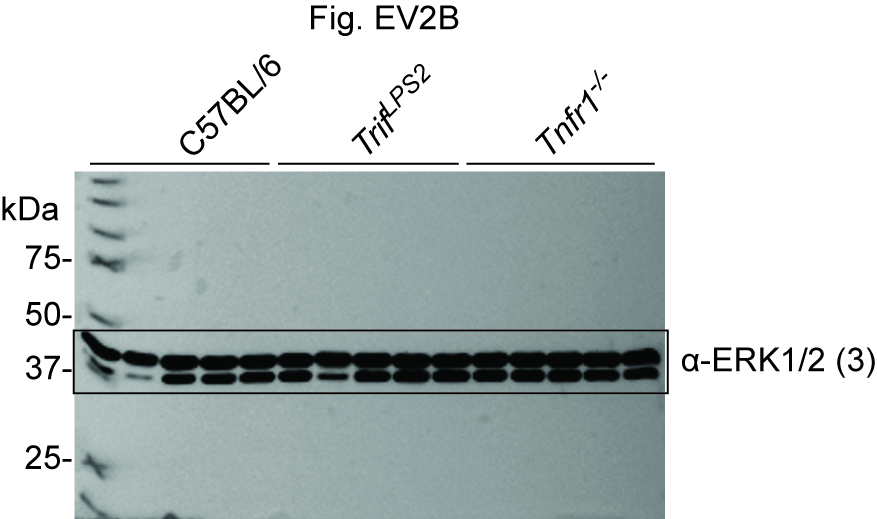

Supplement: Supplementary file 8 — EV Figure Source Data [file 44318_2025_412_MOESM8_ESM.zip › EMBOJ-2024-118621-SourceData_ExpandedViewAndAppendix/Expanded view 2/2B/EV2B ERK1-2.tif]

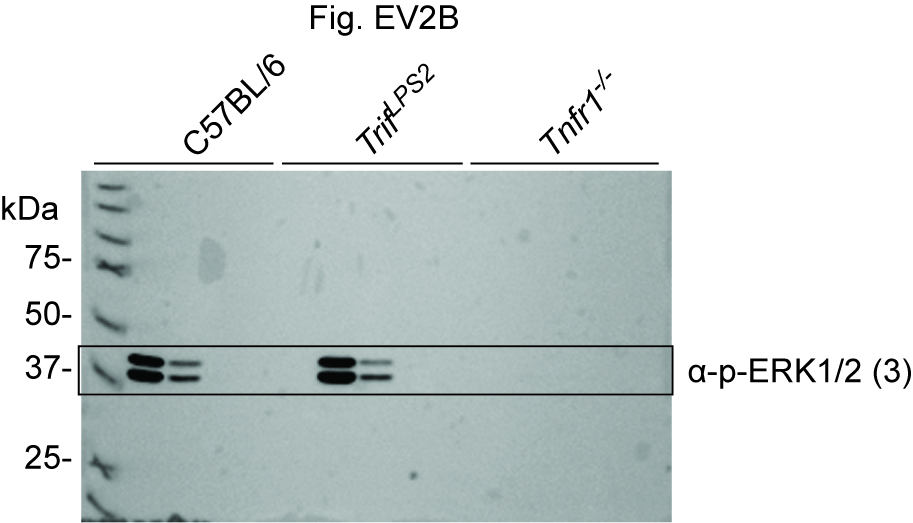

Supplement: Supplementary file 8 — EV Figure Source Data [file 44318_2025_412_MOESM8_ESM.zip › EMBOJ-2024-118621-SourceData_ExpandedViewAndAppendix/Expanded view 2/2B/EV2B p-ERK1-2.tif]

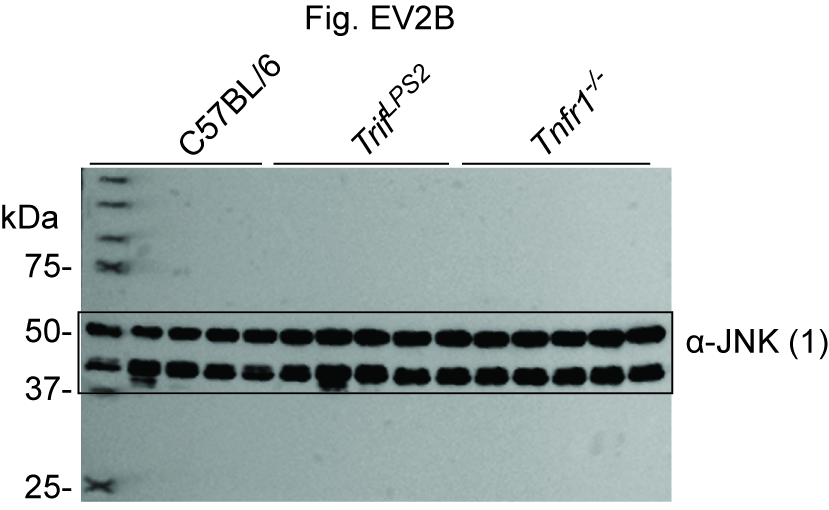

Supplement: Supplementary file 8 — EV Figure Source Data [file 44318_2025_412_MOESM8_ESM.zip › EMBOJ-2024-118621-SourceData_ExpandedViewAndAppendix/Expanded view 2/2B/EV2B JNK.tif]

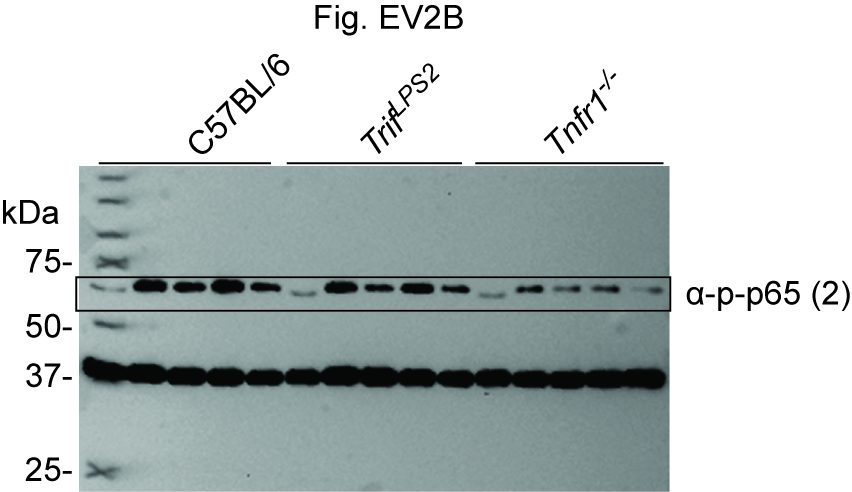

Supplement: Supplementary file 8 — EV Figure Source Data [file 44318_2025_412_MOESM8_ESM.zip › EMBOJ-2024-118621-SourceData_ExpandedViewAndAppendix/Expanded view 2/2B/EV2B p-p65.tif]

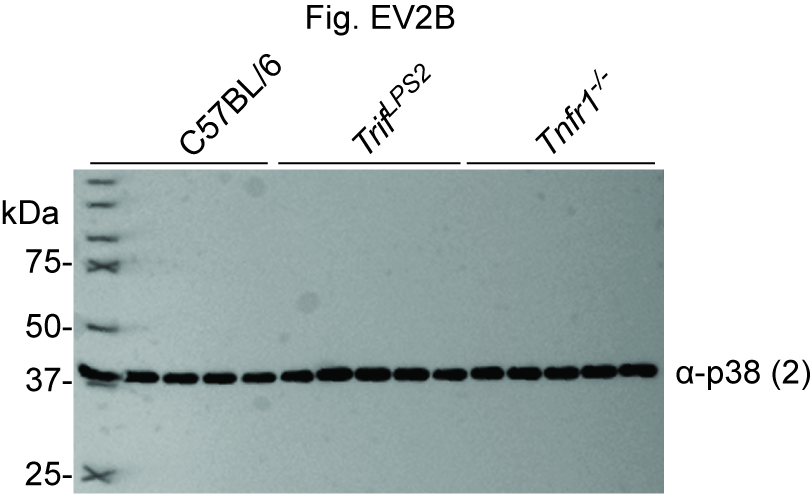

Supplement: Supplementary file 8 — EV Figure Source Data [file 44318_2025_412_MOESM8_ESM.zip › EMBOJ-2024-118621-SourceData_ExpandedViewAndAppendix/Expanded view 2/2B/EV2B p38.tif]

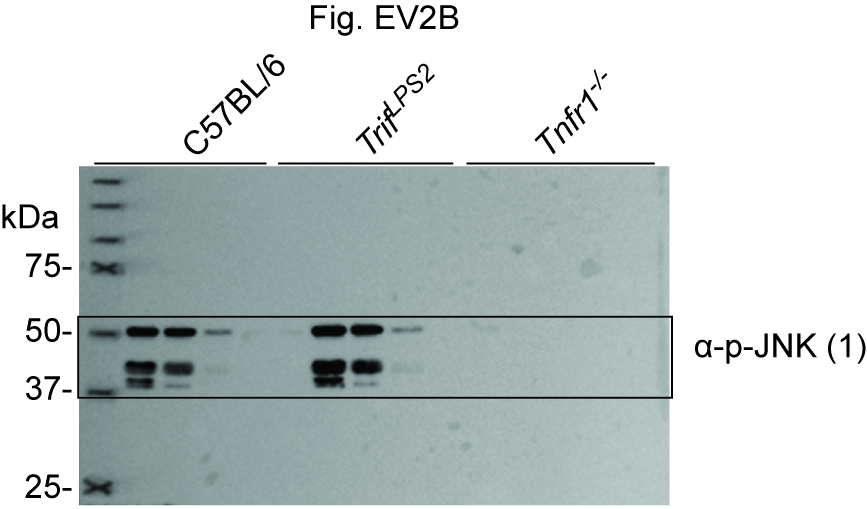

Supplement: Supplementary file 8 — EV Figure Source Data [file 44318_2025_412_MOESM8_ESM.zip › EMBOJ-2024-118621-SourceData_ExpandedViewAndAppendix/Expanded view 2/2B/EV2B p-JNK.tif]

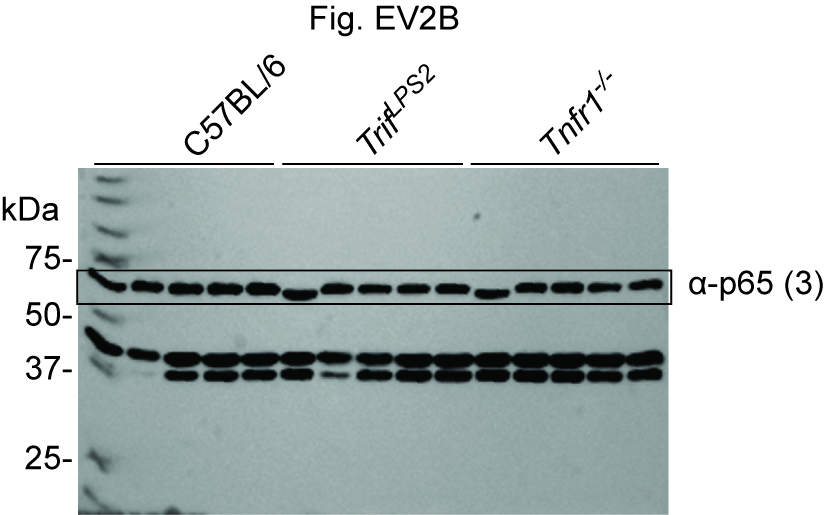

Supplement: Supplementary file 8 — EV Figure Source Data [file 44318_2025_412_MOESM8_ESM.zip › EMBOJ-2024-118621-SourceData_ExpandedViewAndAppendix/Expanded view 2/2B/EV2B p65.tif]

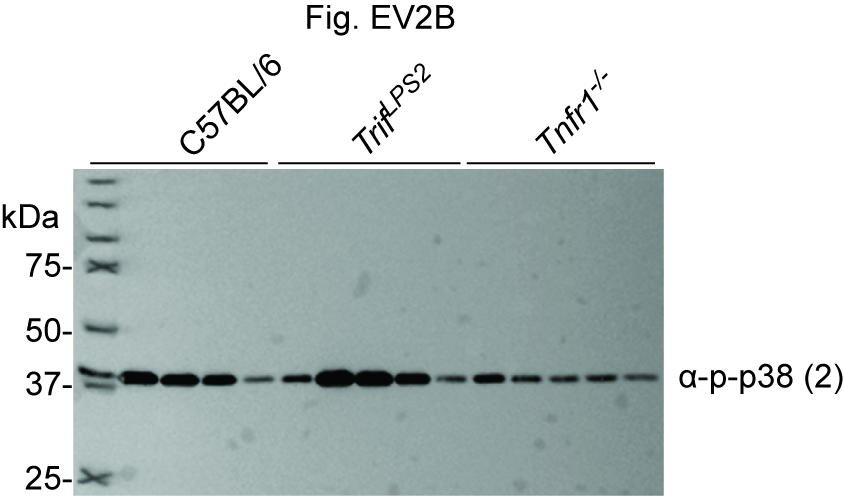

Supplement: Supplementary file 8 — EV Figure Source Data [file 44318_2025_412_MOESM8_ESM.zip › EMBOJ-2024-118621-SourceData_ExpandedViewAndAppendix/Expanded view 2/2B/EV2B p-p38.tif]

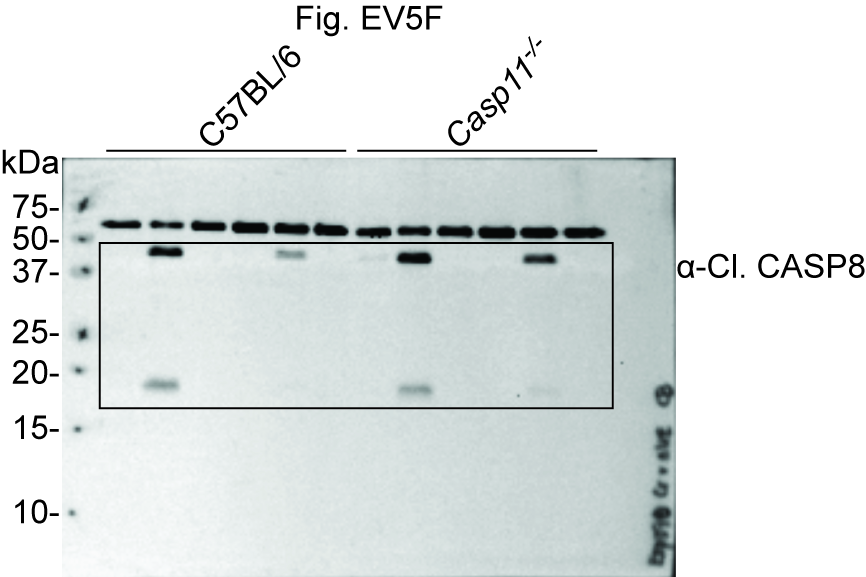

Supplement: Supplementary file 8 — EV Figure Source Data [file 44318_2025_412_MOESM8_ESM.zip › EMBOJ-2024-118621-SourceData_ExpandedViewAndAppendix/Expanded view 5/5F/EV5F Cl Casp8.tif]

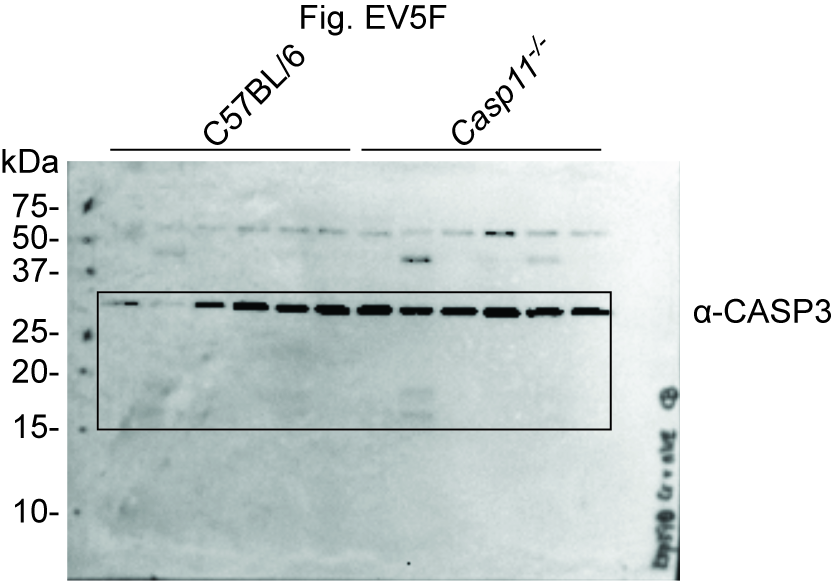

Supplement: Supplementary file 8 — EV Figure Source Data [file 44318_2025_412_MOESM8_ESM.zip › EMBOJ-2024-118621-SourceData_ExpandedViewAndAppendix/Expanded view 5/5F/EV5F Casp3.tif]

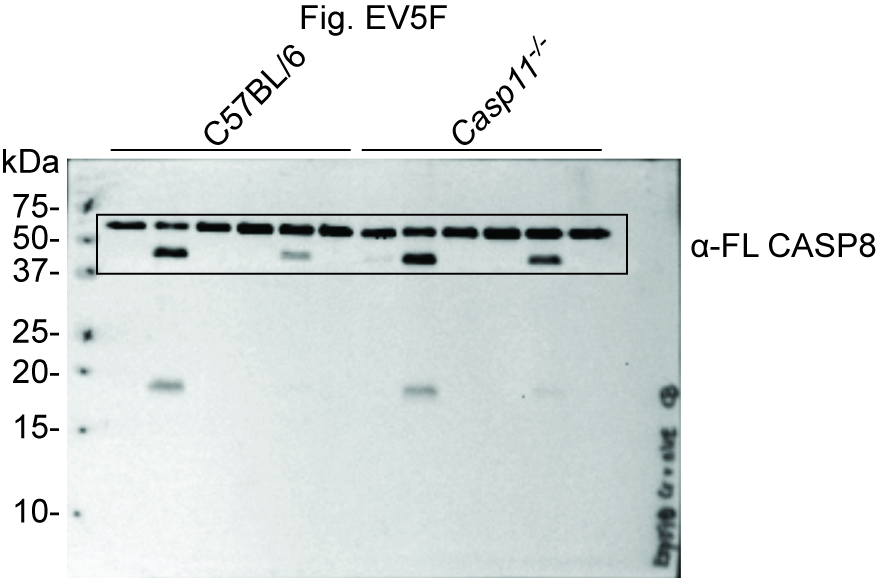

Supplement: Supplementary file 8 — EV Figure Source Data [file 44318_2025_412_MOESM8_ESM.zip › EMBOJ-2024-118621-SourceData_ExpandedViewAndAppendix/Expanded view 5/5F/EV5F FL Casp8.tif]

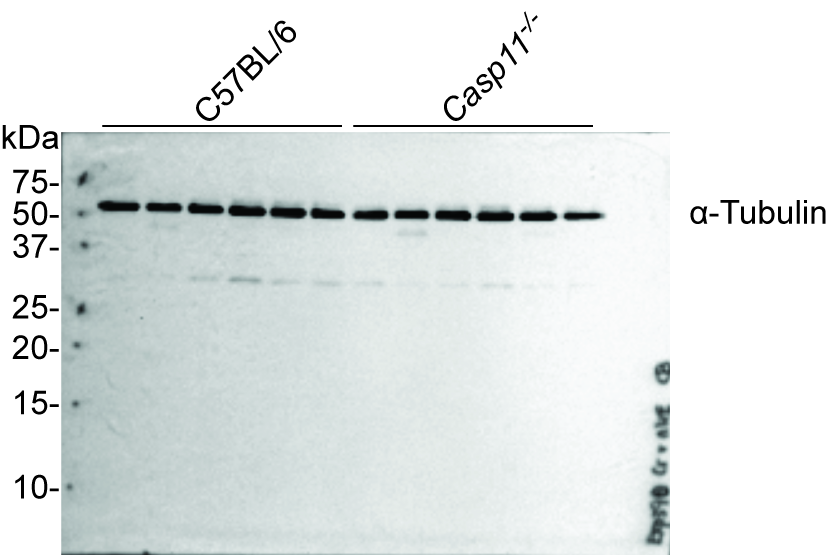

Supplement: Supplementary file 8 — EV Figure Source Data [file 44318_2025_412_MOESM8_ESM.zip › EMBOJ-2024-118621-SourceData_ExpandedViewAndAppendix/Expanded view 5/5F/EV5F Tubulin.tif]

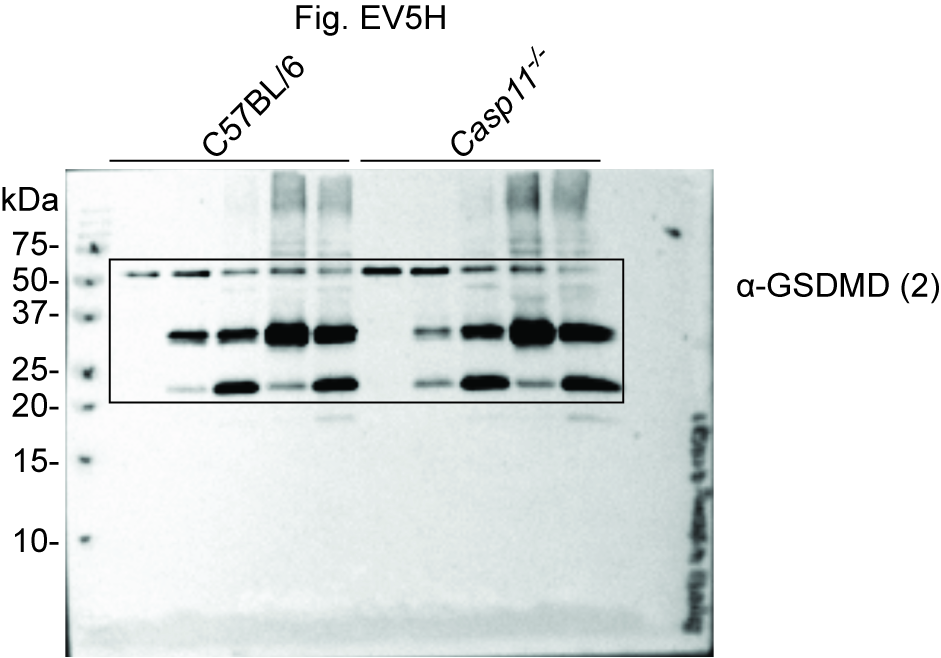

Supplement: Supplementary file 8 — EV Figure Source Data [file 44318_2025_412_MOESM8_ESM.zip › EMBOJ-2024-118621-SourceData_ExpandedViewAndAppendix/Expanded view 5/5H/EV5H GSDMD.tif]

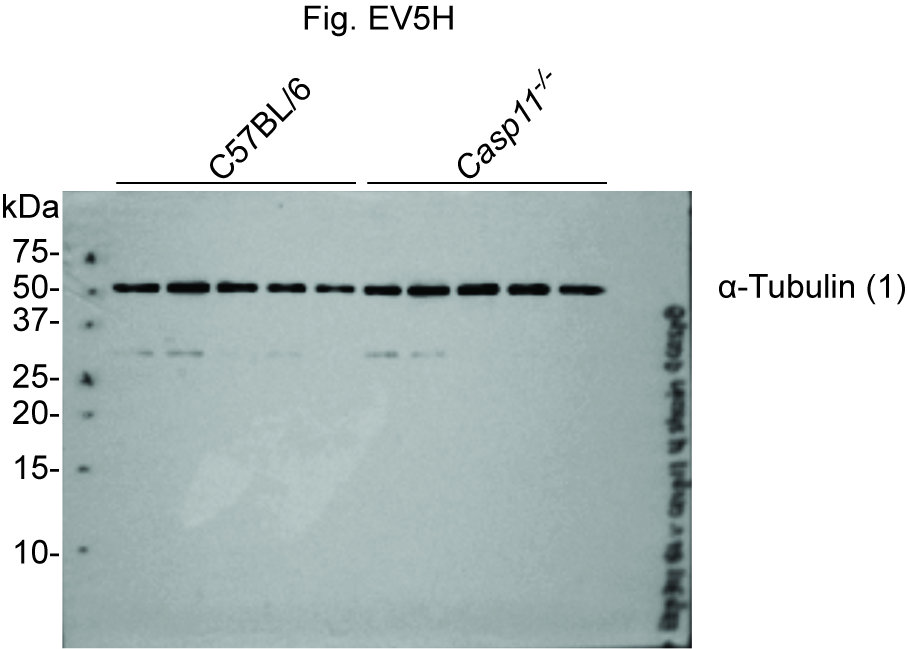

Supplement: Supplementary file 8 — EV Figure Source Data [file 44318_2025_412_MOESM8_ESM.zip › EMBOJ-2024-118621-SourceData_ExpandedViewAndAppendix/Expanded view 5/5H/EV5H Tubulin 1.tif]

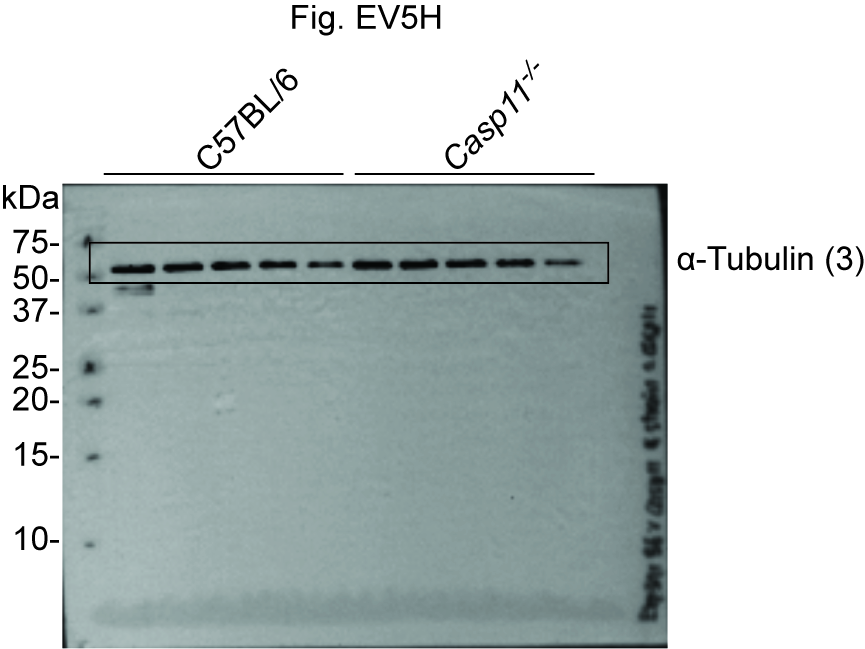

Supplement: Supplementary file 8 — EV Figure Source Data [file 44318_2025_412_MOESM8_ESM.zip › EMBOJ-2024-118621-SourceData_ExpandedViewAndAppendix/Expanded view 5/5H/EV5H Tubulin 3.tif]

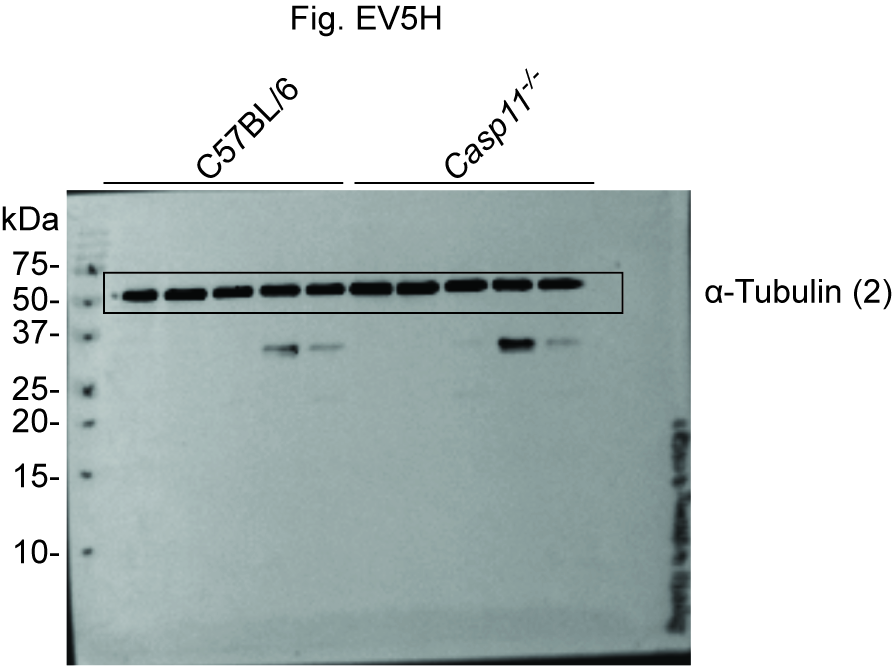

Supplement: Supplementary file 8 — EV Figure Source Data [file 44318_2025_412_MOESM8_ESM.zip › EMBOJ-2024-118621-SourceData_ExpandedViewAndAppendix/Expanded view 5/5H/EV5H Tubulin 2.tif]

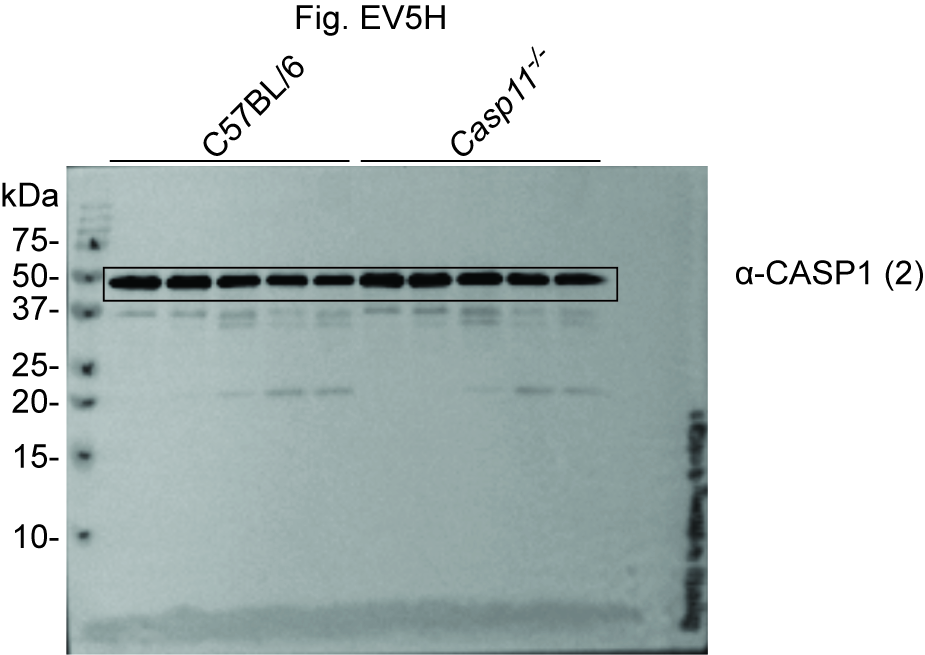

Supplement: Supplementary file 8 — EV Figure Source Data [file 44318_2025_412_MOESM8_ESM.zip › EMBOJ-2024-118621-SourceData_ExpandedViewAndAppendix/Expanded view 5/5H/EV5H Casp1.tif]

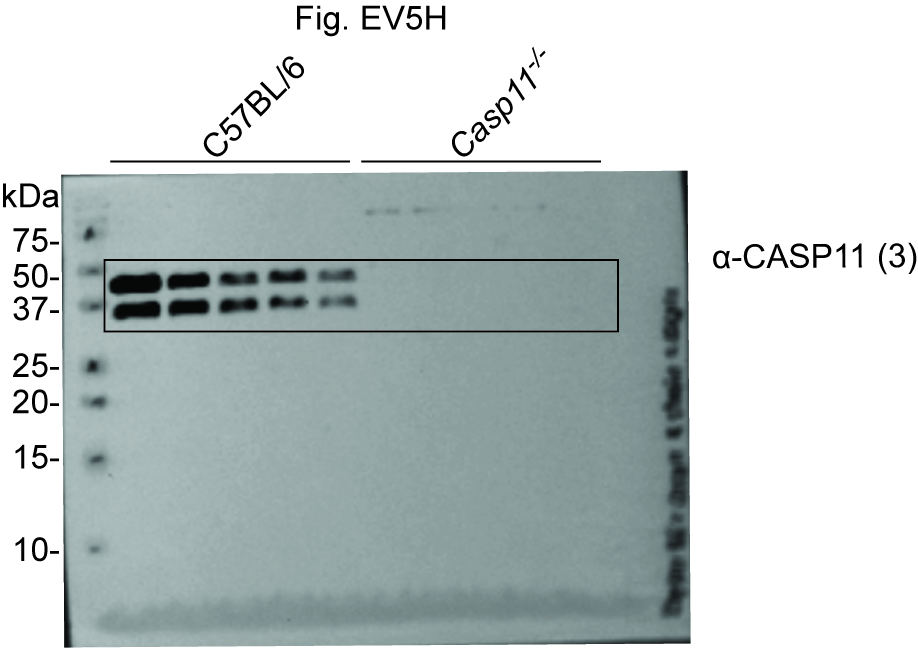

Supplement: Supplementary file 8 — EV Figure Source Data [file 44318_2025_412_MOESM8_ESM.zip › EMBOJ-2024-118621-SourceData_ExpandedViewAndAppendix/Expanded view 5/5H/EV5H Casp11.tif]

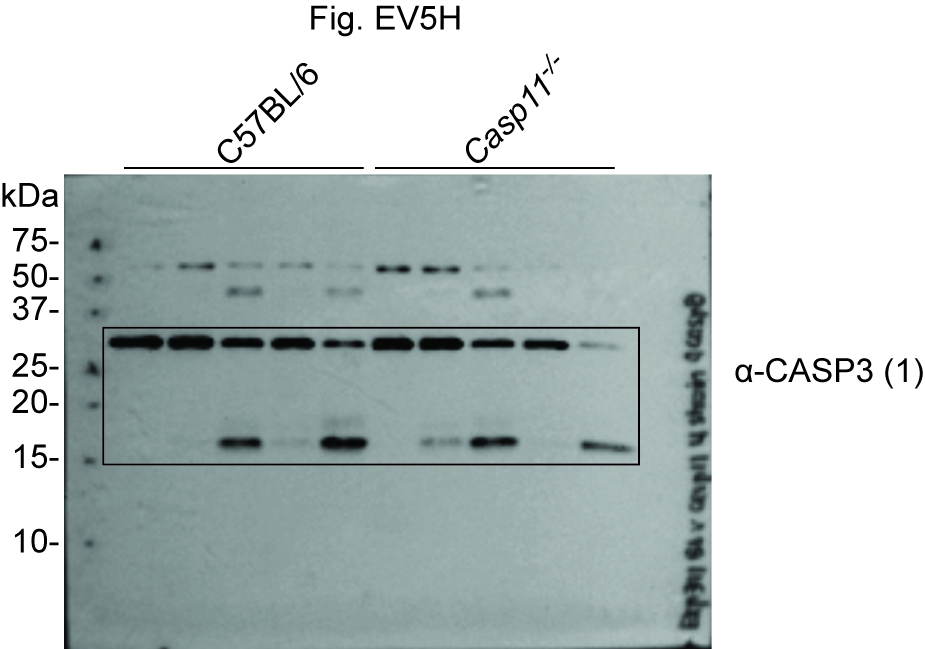

Supplement: Supplementary file 8 — EV Figure Source Data [file 44318_2025_412_MOESM8_ESM.zip › EMBOJ-2024-118621-SourceData_ExpandedViewAndAppendix/Expanded view 5/5H/EV5H Casp3.tif]

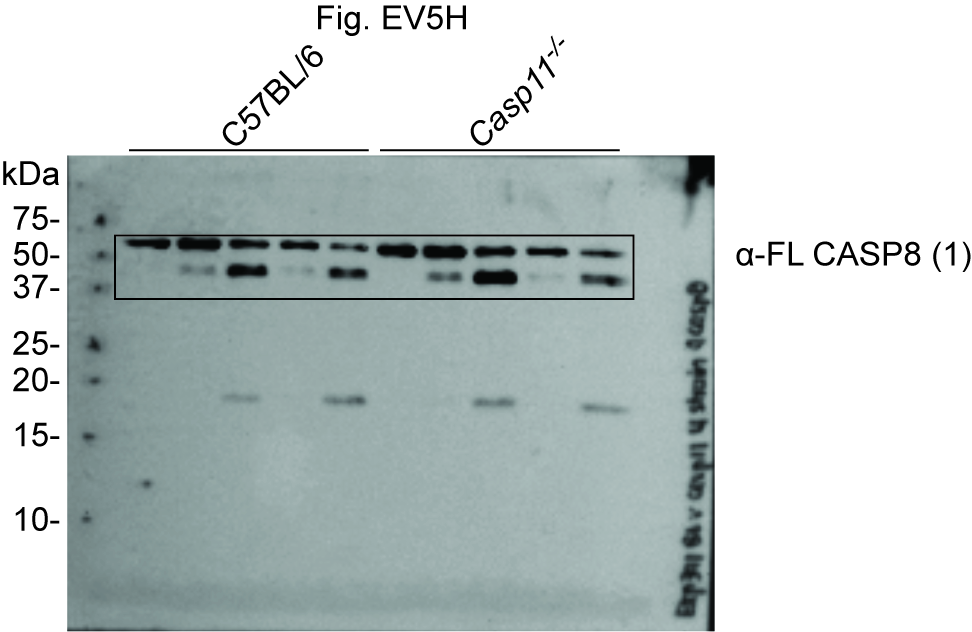

Supplement: Supplementary file 8 — EV Figure Source Data [file 44318_2025_412_MOESM8_ESM.zip › EMBOJ-2024-118621-SourceData_ExpandedViewAndAppendix/Expanded view 5/5H/EV5H FL Casp8.tif]

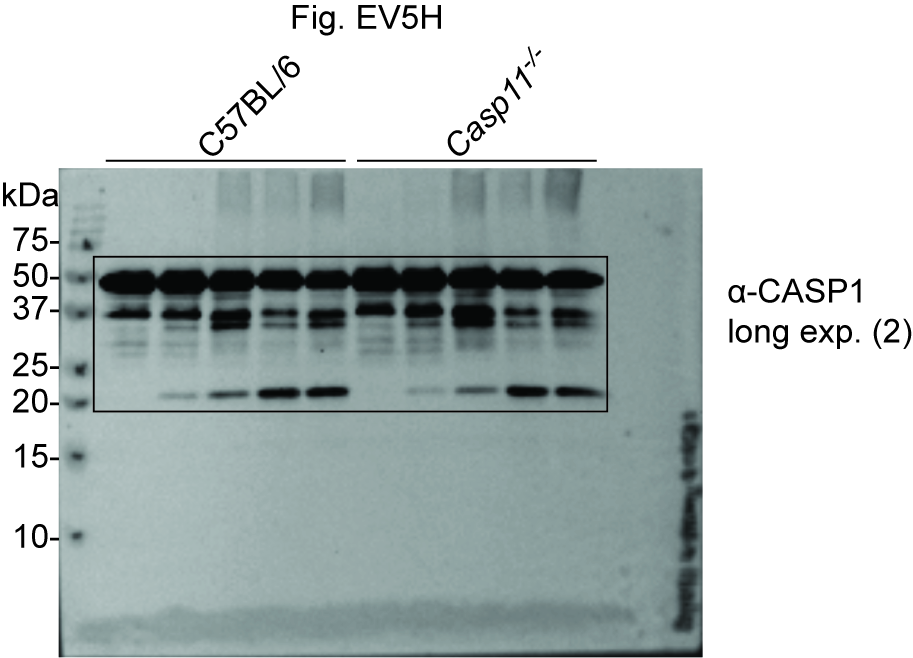

Supplement: Supplementary file 8 — EV Figure Source Data [file 44318_2025_412_MOESM8_ESM.zip › EMBOJ-2024-118621-SourceData_ExpandedViewAndAppendix/Expanded view 5/5H/EV5H Casp1 long exposure.tif]

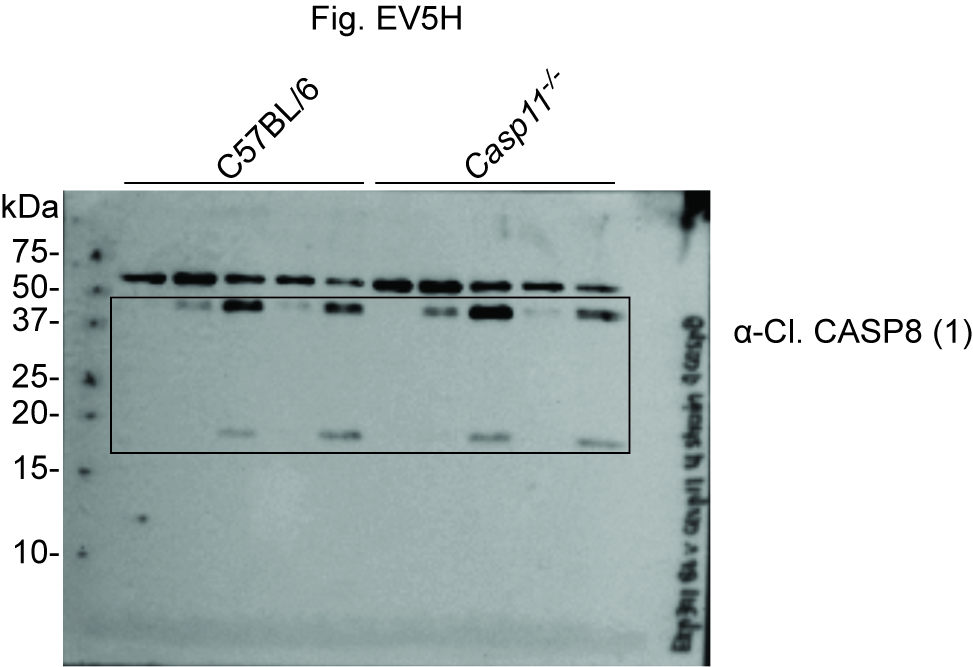

Supplement: Supplementary file 8 — EV Figure Source Data [file 44318_2025_412_MOESM8_ESM.zip › EMBOJ-2024-118621-SourceData_ExpandedViewAndAppendix/Expanded view 5/5H/EV5H Cl Casp8.tif]

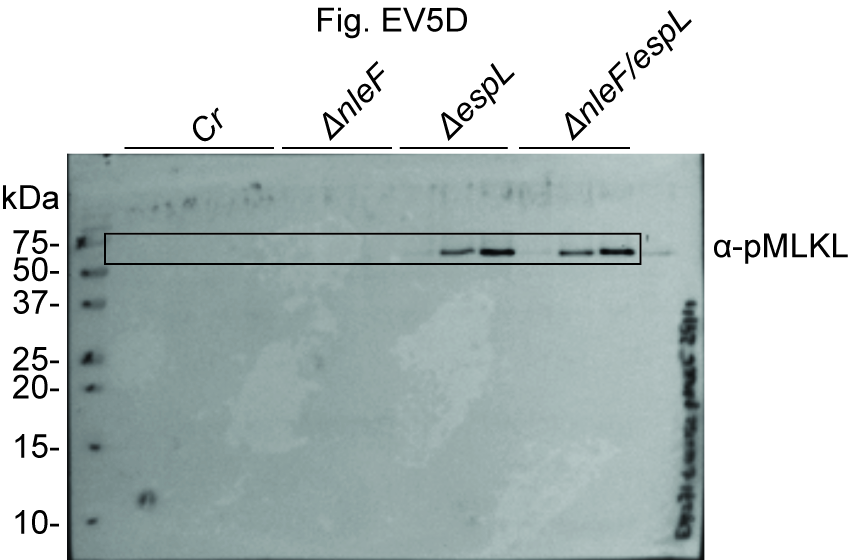

Supplement: Supplementary file 8 — EV Figure Source Data [file 44318_2025_412_MOESM8_ESM.zip › EMBOJ-2024-118621-SourceData_ExpandedViewAndAppendix/Expanded view 5/5D/EV5D pMLKL.tif]

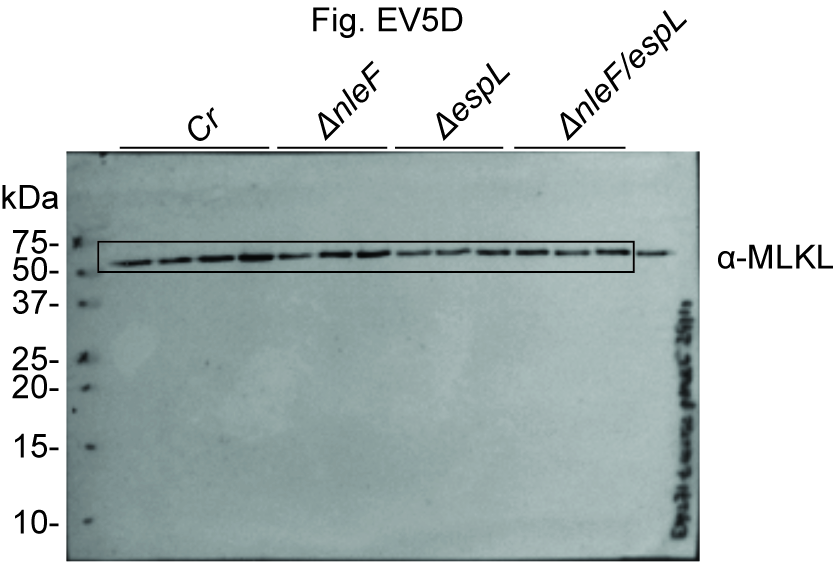

Supplement: Supplementary file 8 — EV Figure Source Data [file 44318_2025_412_MOESM8_ESM.zip › EMBOJ-2024-118621-SourceData_ExpandedViewAndAppendix/Expanded view 5/5D/EV5D MLKL.tif]

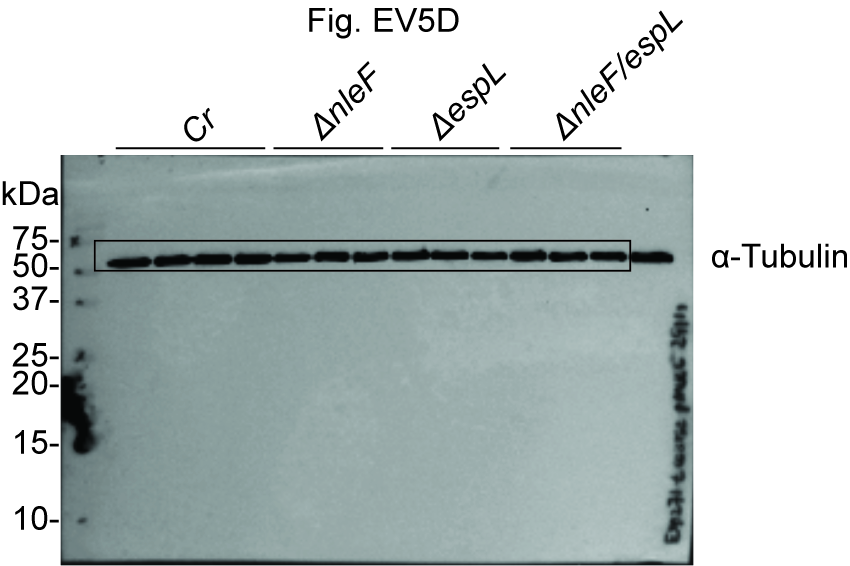

Supplement: Supplementary file 8 — EV Figure Source Data [file 44318_2025_412_MOESM8_ESM.zip › EMBOJ-2024-118621-SourceData_ExpandedViewAndAppendix/Expanded view 5/5D/EV5D Tubulin.tif]

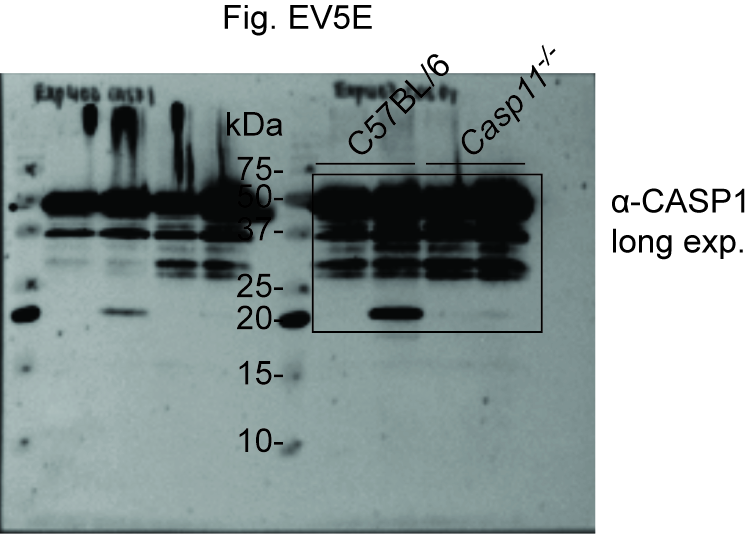

Supplement: Supplementary file 8 — EV Figure Source Data [file 44318_2025_412_MOESM8_ESM.zip › EMBOJ-2024-118621-SourceData_ExpandedViewAndAppendix/Expanded view 5/5E/EV5E Casp1 long exposure.tif]

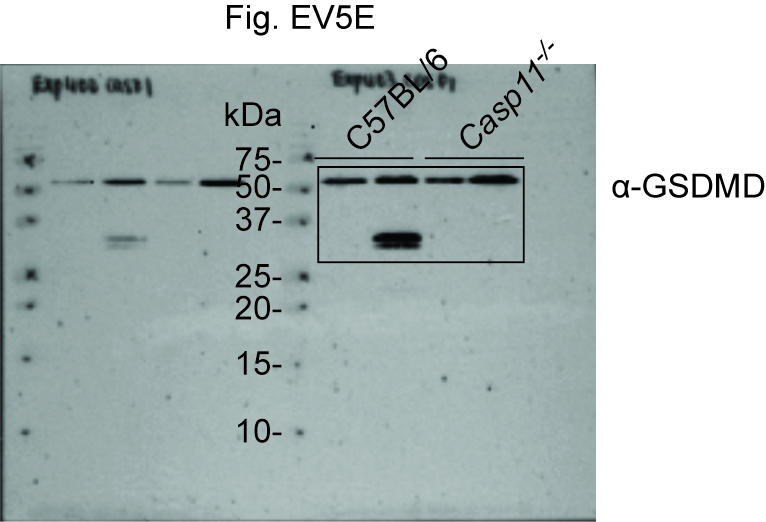

Supplement: Supplementary file 8 — EV Figure Source Data [file 44318_2025_412_MOESM8_ESM.zip › EMBOJ-2024-118621-SourceData_ExpandedViewAndAppendix/Expanded view 5/5E/EV5E GSDMD.tif]

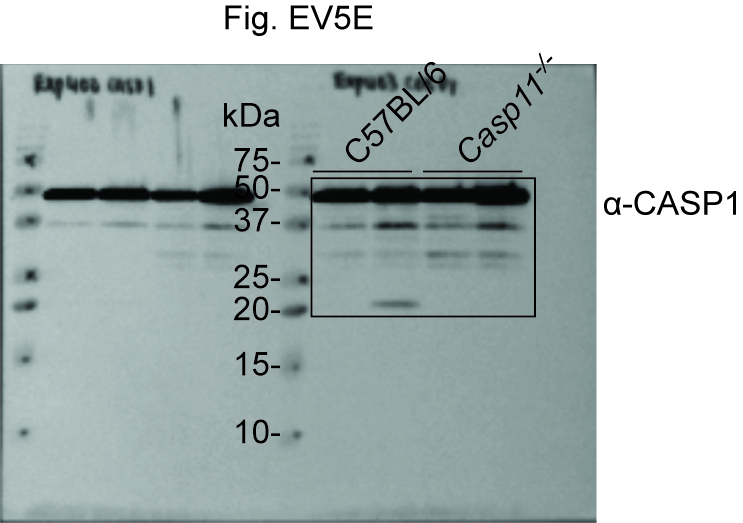

Supplement: Supplementary file 8 — EV Figure Source Data [file 44318_2025_412_MOESM8_ESM.zip › EMBOJ-2024-118621-SourceData_ExpandedViewAndAppendix/Expanded view 5/5E/EV5E Casp1.tif]

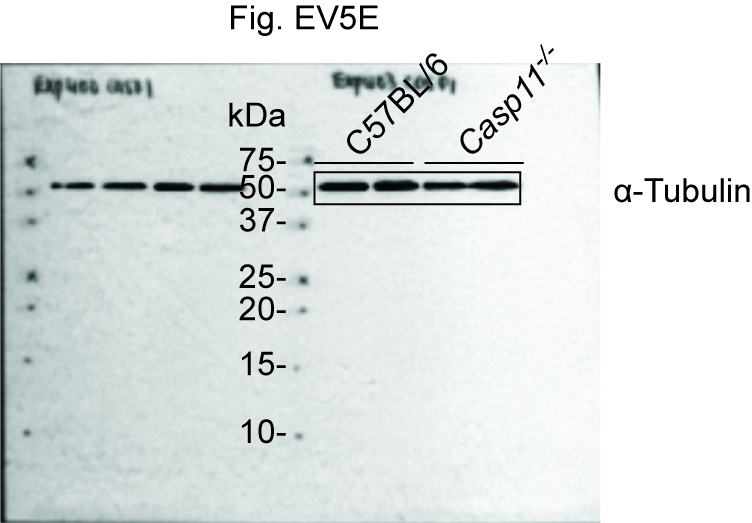

Supplement: Supplementary file 8 — EV Figure Source Data [file 44318_2025_412_MOESM8_ESM.zip › EMBOJ-2024-118621-SourceData_ExpandedViewAndAppendix/Expanded view 5/5E/EV5E Tubulin.tif]

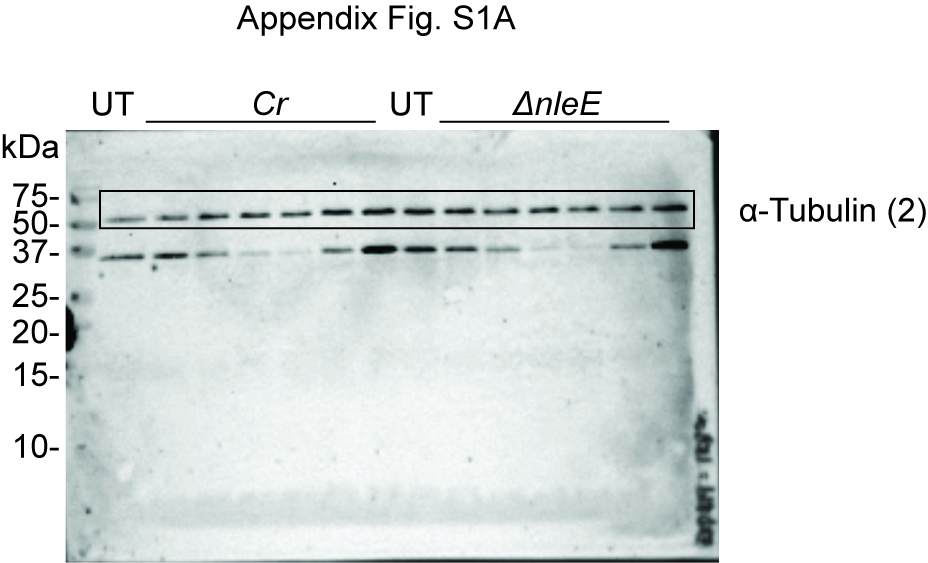

Supplement: Supplementary file 8 — EV Figure Source Data [file 44318_2025_412_MOESM8_ESM.zip › EMBOJ-2024-118621-SourceData_ExpandedViewAndAppendix/Appendix Figure 1/1A/S1A Tubulin 2.tif]

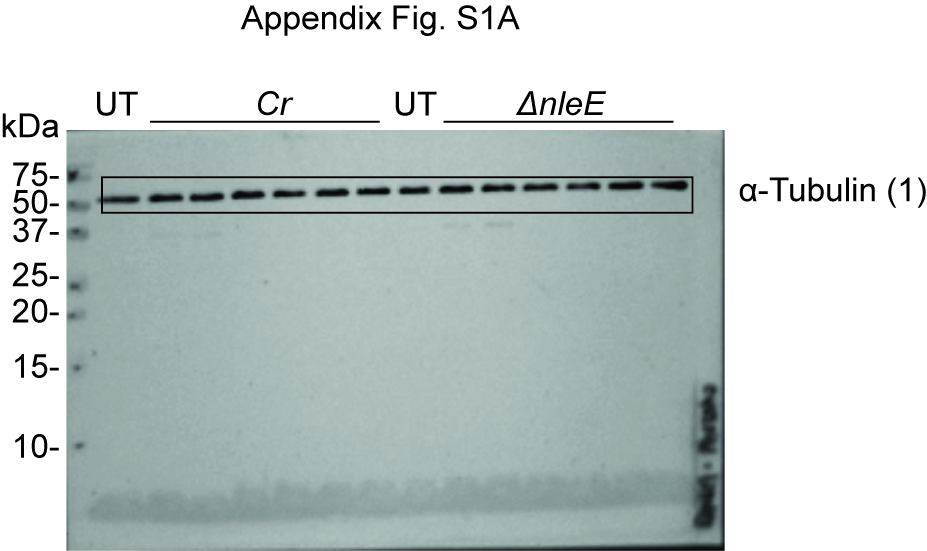

Supplement: Supplementary file 8 — EV Figure Source Data [file 44318_2025_412_MOESM8_ESM.zip › EMBOJ-2024-118621-SourceData_ExpandedViewAndAppendix/Appendix Figure 1/1A/S1A Tubulin 1.tif]

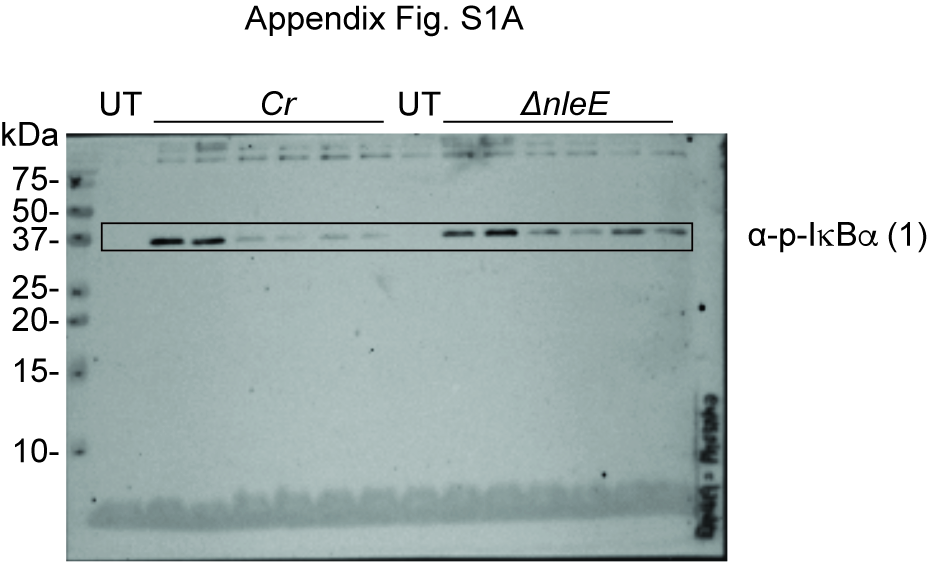

Supplement: Supplementary file 8 — EV Figure Source Data [file 44318_2025_412_MOESM8_ESM.zip › EMBOJ-2024-118621-SourceData_ExpandedViewAndAppendix/Appendix Figure 1/1A/S1A pIkBa.tif]

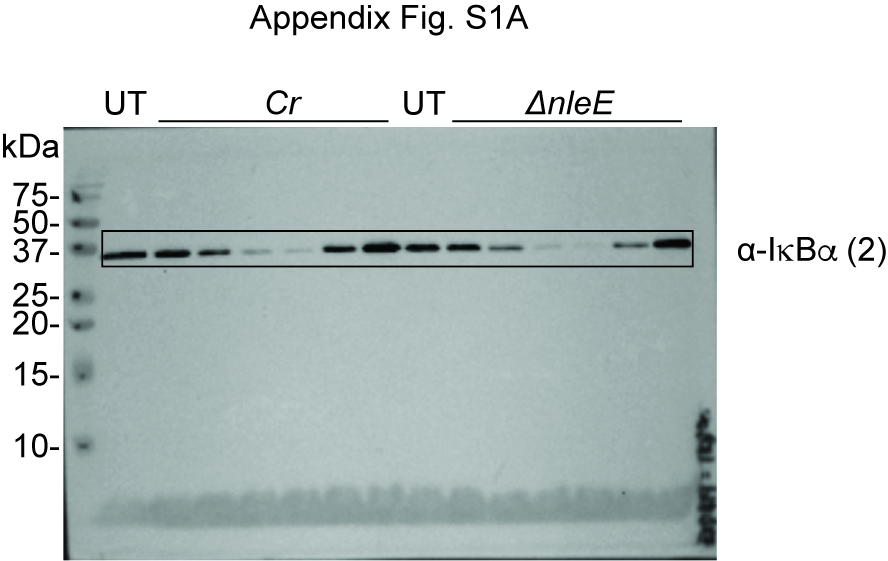

Supplement: Supplementary file 8 — EV Figure Source Data [file 44318_2025_412_MOESM8_ESM.zip › EMBOJ-2024-118621-SourceData_ExpandedViewAndAppendix/Appendix Figure 1/1A/S1A IkBa.tif]

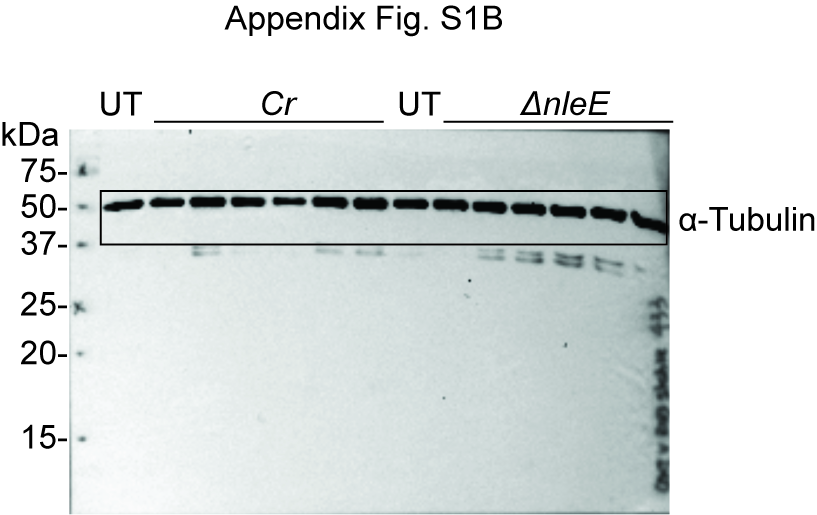

Supplement: Supplementary file 8 — EV Figure Source Data [file 44318_2025_412_MOESM8_ESM.zip › EMBOJ-2024-118621-SourceData_ExpandedViewAndAppendix/Appendix Figure 1/1B/S1B Tubulin.tif]

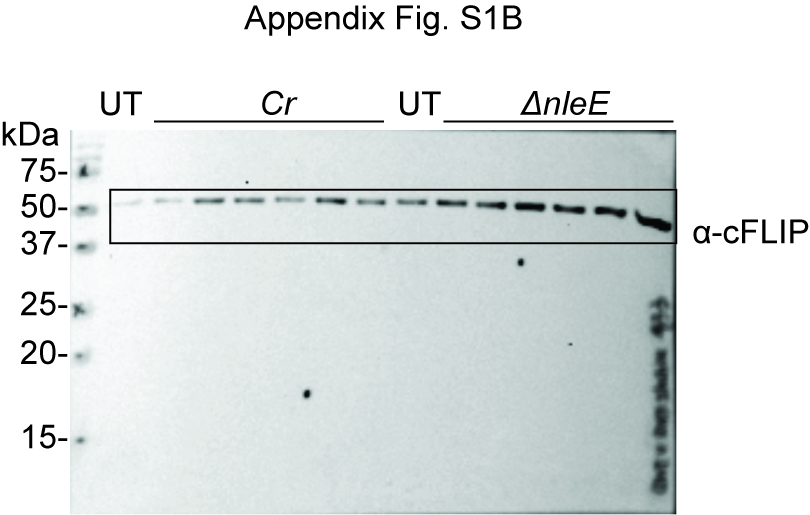

Supplement: Supplementary file 8 — EV Figure Source Data [file 44318_2025_412_MOESM8_ESM.zip › EMBOJ-2024-118621-SourceData_ExpandedViewAndAppendix/Appendix Figure 1/1B/S1B cFLIP.tif]

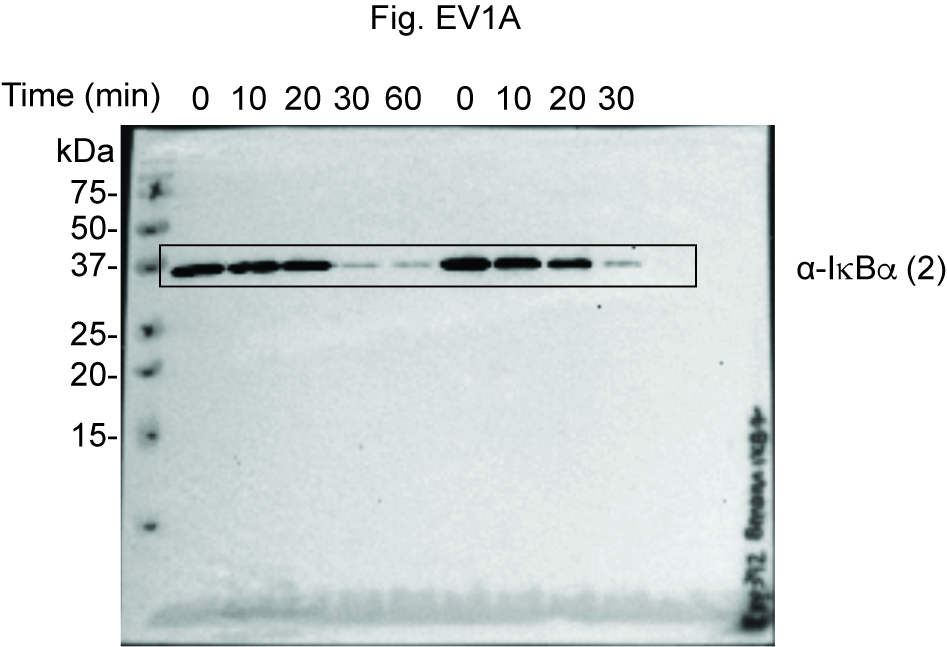

Supplement: Supplementary file 8 — EV Figure Source Data [file 44318_2025_412_MOESM8_ESM.zip › EMBOJ-2024-118621-SourceData_ExpandedViewAndAppendix/Expanded view 1/1A/EV1A IkBa.tif]

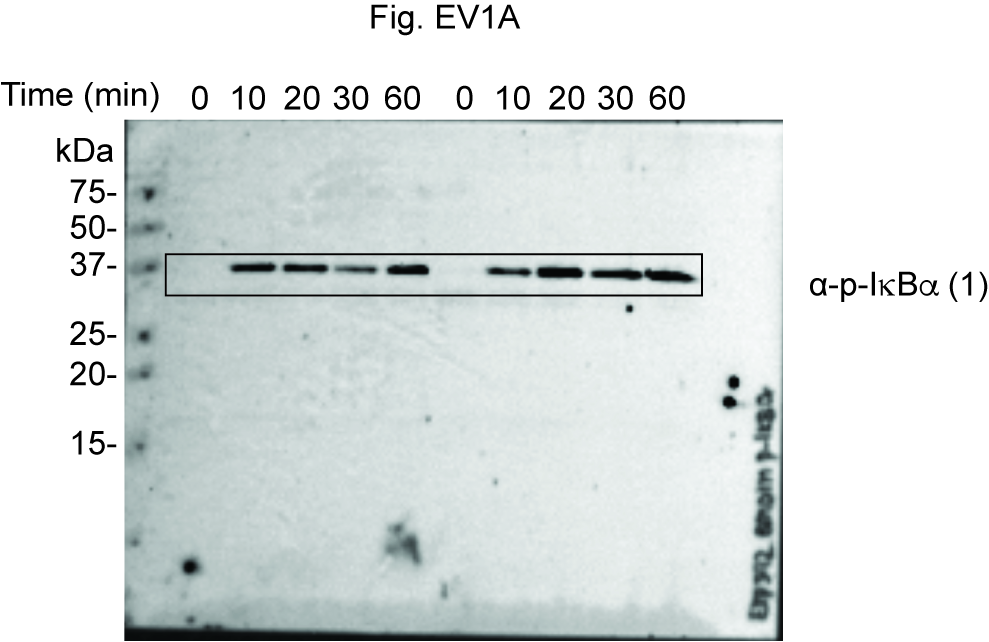

Supplement: Supplementary file 8 — EV Figure Source Data [file 44318_2025_412_MOESM8_ESM.zip › EMBOJ-2024-118621-SourceData_ExpandedViewAndAppendix/Expanded view 1/1A/EV1A p-IkBa.tif]

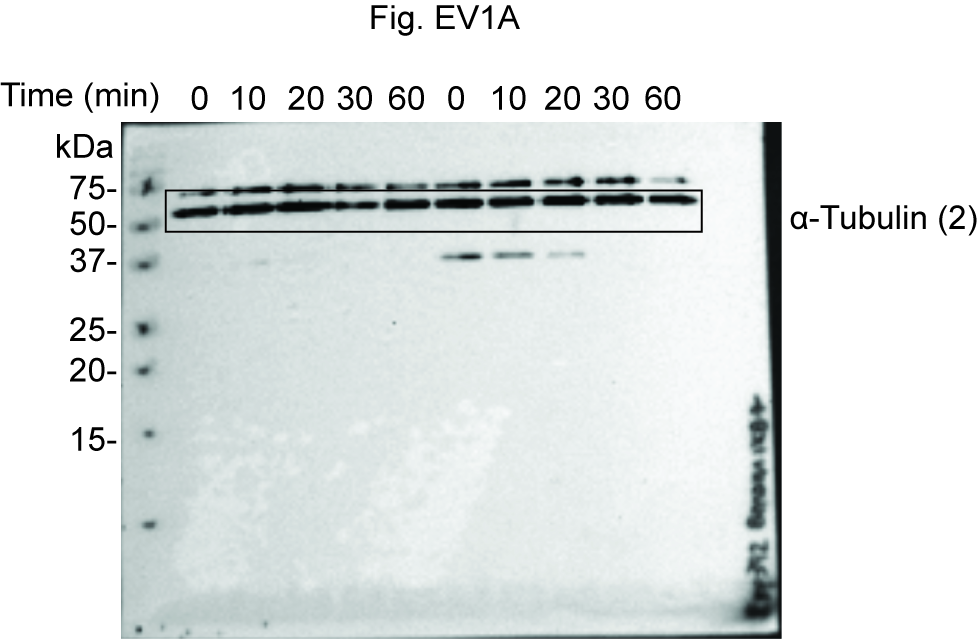

Supplement: Supplementary file 8 — EV Figure Source Data [file 44318_2025_412_MOESM8_ESM.zip › EMBOJ-2024-118621-SourceData_ExpandedViewAndAppendix/Expanded view 1/1A/EV1A Tubulin 2.tif]

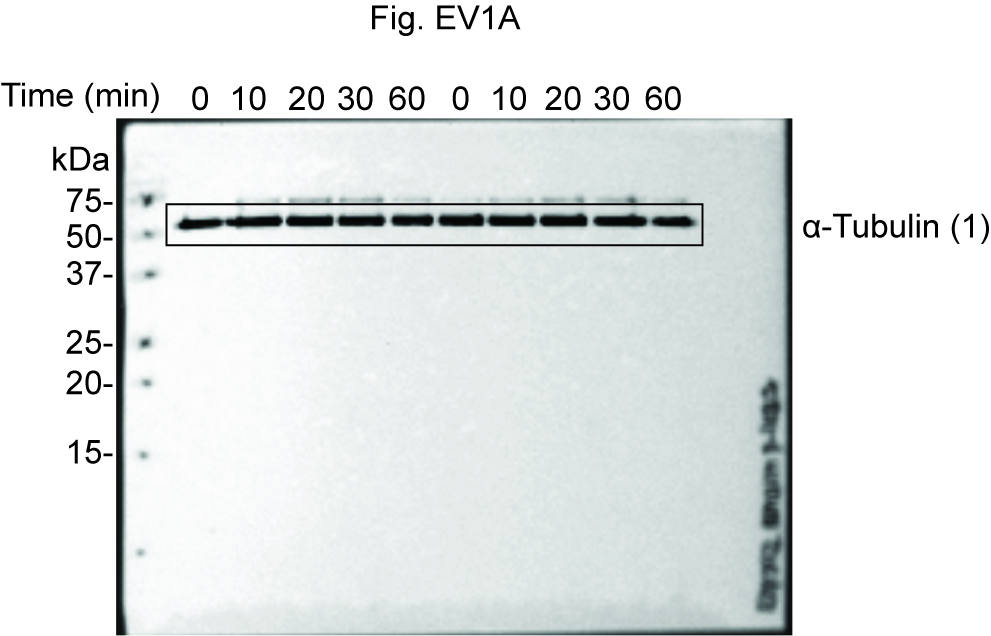

Supplement: Supplementary file 8 — EV Figure Source Data [file 44318_2025_412_MOESM8_ESM.zip › EMBOJ-2024-118621-SourceData_ExpandedViewAndAppendix/Expanded view 1/1A/EV1A Tubulin 1.tif]

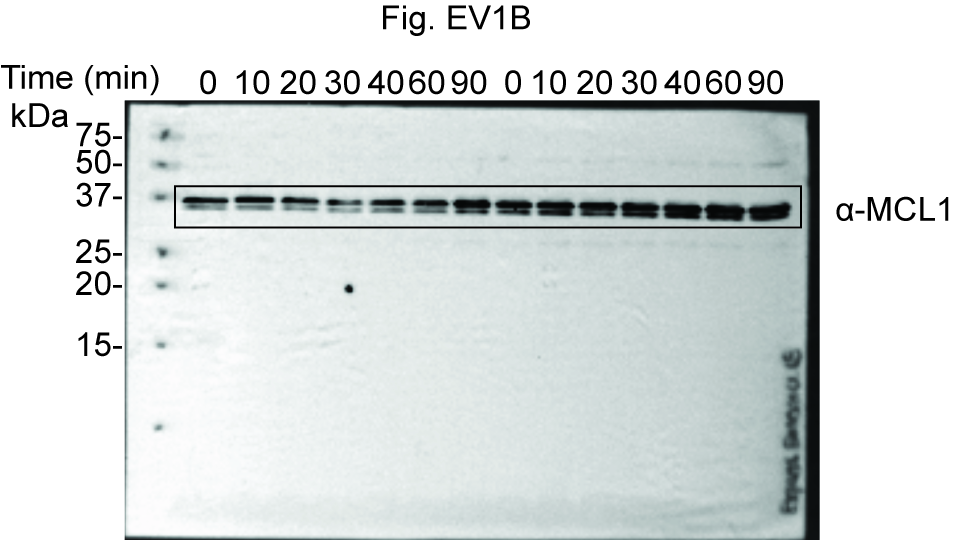

Supplement: Supplementary file 8 — EV Figure Source Data [file 44318_2025_412_MOESM8_ESM.zip › EMBOJ-2024-118621-SourceData_ExpandedViewAndAppendix/Expanded view 1/1B/EV1B MCL-1.tif]

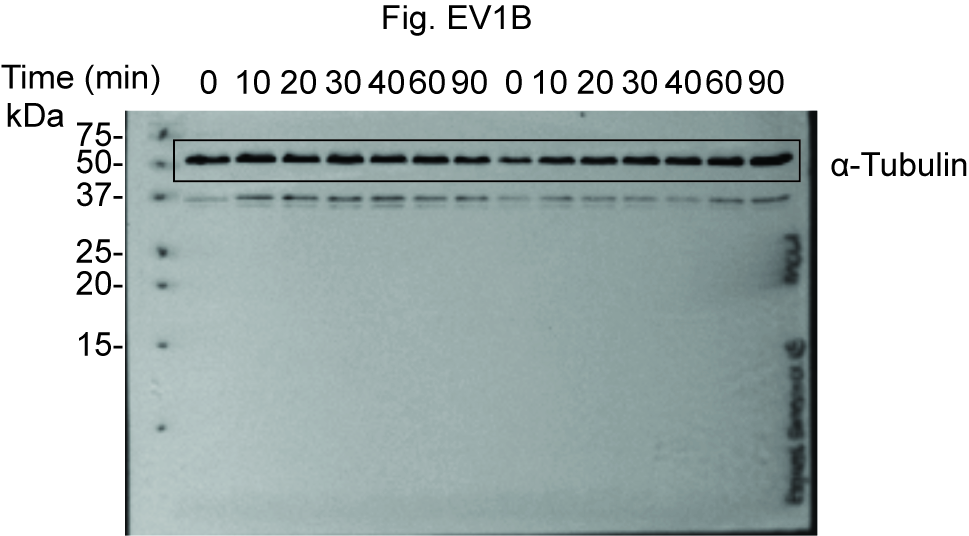

Supplement: Supplementary file 8 — EV Figure Source Data [file 44318_2025_412_MOESM8_ESM.zip › EMBOJ-2024-118621-SourceData_ExpandedViewAndAppendix/Expanded view 1/1B/EV1B Tubulin.tif]

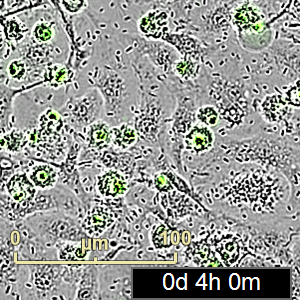

Supplement: Supplementary file 8 — EV Figure Source Data [file 44318_2025_412_MOESM8_ESM.zip › EMBOJ-2024-118621-SourceData_ExpandedViewAndAppendix/Appendix Figure 2/2A/Cr_4h.tif]

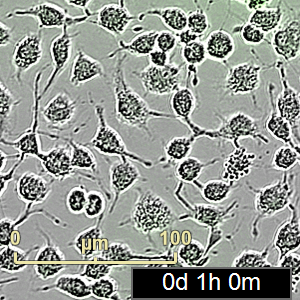

Supplement: Supplementary file 8 — EV Figure Source Data [file 44318_2025_412_MOESM8_ESM.zip › EMBOJ-2024-118621-SourceData_ExpandedViewAndAppendix/Appendix Figure 2/2A/Yptb_1h.tif]

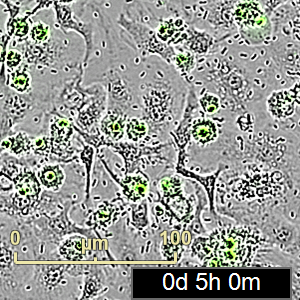

Supplement: Supplementary file 8 — EV Figure Source Data [file 44318_2025_412_MOESM8_ESM.zip › EMBOJ-2024-118621-SourceData_ExpandedViewAndAppendix/Appendix Figure 2/2A/Cr_5h.tif]

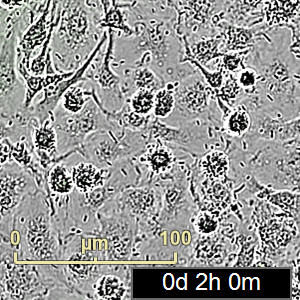

Supplement: Supplementary file 8 — EV Figure Source Data [file 44318_2025_412_MOESM8_ESM.zip › EMBOJ-2024-118621-SourceData_ExpandedViewAndAppendix/Appendix Figure 2/2A/Cr_2h.tif]

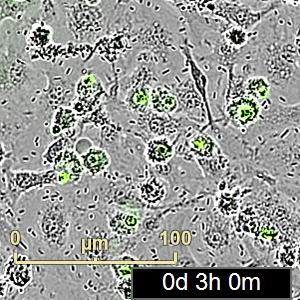

Supplement: Supplementary file 8 — EV Figure Source Data [file 44318_2025_412_MOESM8_ESM.zip › EMBOJ-2024-118621-SourceData_ExpandedViewAndAppendix/Appendix Figure 2/2A/Cr_3h.tif]

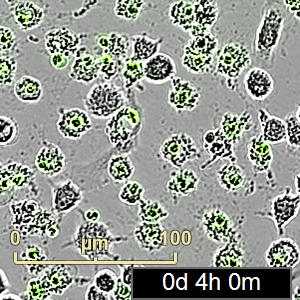

Supplement: Supplementary file 8 — EV Figure Source Data [file 44318_2025_412_MOESM8_ESM.zip › EMBOJ-2024-118621-SourceData_ExpandedViewAndAppendix/Appendix Figure 2/2A/Yptb_4h.tif]

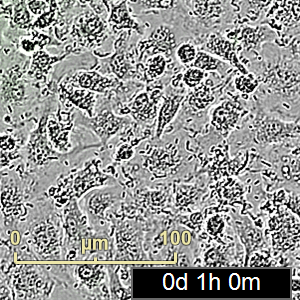

Supplement: Supplementary file 8 — EV Figure Source Data [file 44318_2025_412_MOESM8_ESM.zip › EMBOJ-2024-118621-SourceData_ExpandedViewAndAppendix/Appendix Figure 2/2A/Cr_1h.tif]

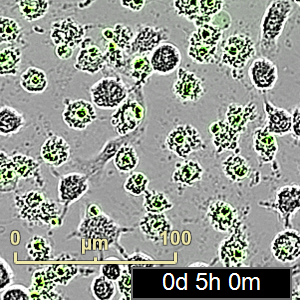

Supplement: Supplementary file 8 — EV Figure Source Data [file 44318_2025_412_MOESM8_ESM.zip › EMBOJ-2024-118621-SourceData_ExpandedViewAndAppendix/Appendix Figure 2/2A/Yptb_5h.tif]

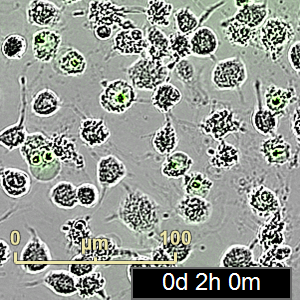

Supplement: Supplementary file 8 — EV Figure Source Data [file 44318_2025_412_MOESM8_ESM.zip › EMBOJ-2024-118621-SourceData_ExpandedViewAndAppendix/Appendix Figure 2/2A/Yptb_2h.tif]

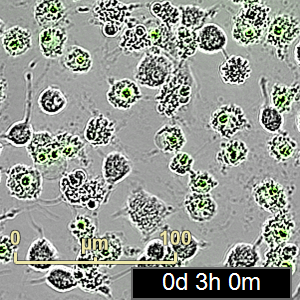

Supplement: Supplementary file 8 — EV Figure Source Data [file 44318_2025_412_MOESM8_ESM.zip › EMBOJ-2024-118621-SourceData_ExpandedViewAndAppendix/Appendix Figure 2/2A/Yptb_3h.tif]
