# Supplementary material for: A bacterial network of T3SS effectors counteracts host pro-inflammatory responses and cell death to promote infection
Source: EMBO J. 2025 Mar 24;44(9):2424–45. doi: 10.1038/s44318-025-00412-5 (PMC12048508; doi:10.1038/s44318-025-00412-5)
Supplement: Supplementary file 9 — Expanded View Figures [file 44318_2025_412_MOESM9_ESM.pdf]

Expanded View Figures

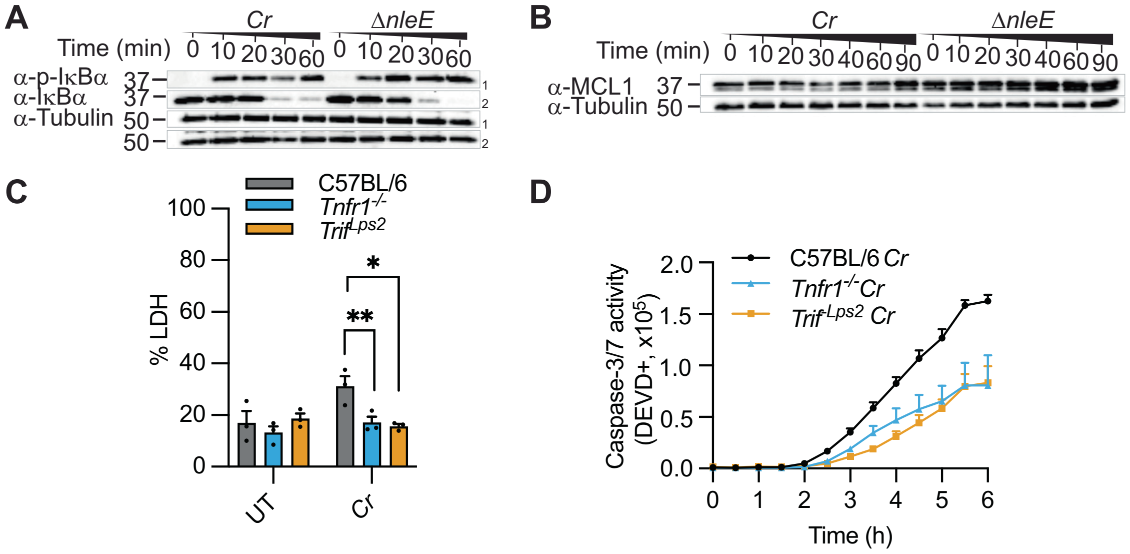

Figure EV1. NleE marginally suppresses NF-κB signalling in macrophages.

(**A–D**) Unprimed BMDMs were challenged with log-phase *C. rodentium* (*Cr*) or Δ*nleE* for (**A**, **B**, **D**) the indicated time points or (**C**) 3 h. (**A**, **B**) Cell lysates were examined by immunoblotting. (**C**) LDH release was measured. (**D**) Caspase-3/7 activity (DEVD-positive) was quantified using IncuCyte. (**C**) Pooled data are mean ± SEM of three independent experiments ($P = 0.0104$ for *Tnfr1*$^{-/-}$ and $P = 0.0056$ for *Trif*^{Lps2}). (**D**) Data are mean ± SD of technical triplicates representative of three independent experiments. All *P* values were calculated with two-way ANOVA test. Data are considered significant when $P \le 0.05$, with $^*P \le 0.05$ or $^{**}P \le 0.01$. Source data are available online for this figure.

© The Author(s)

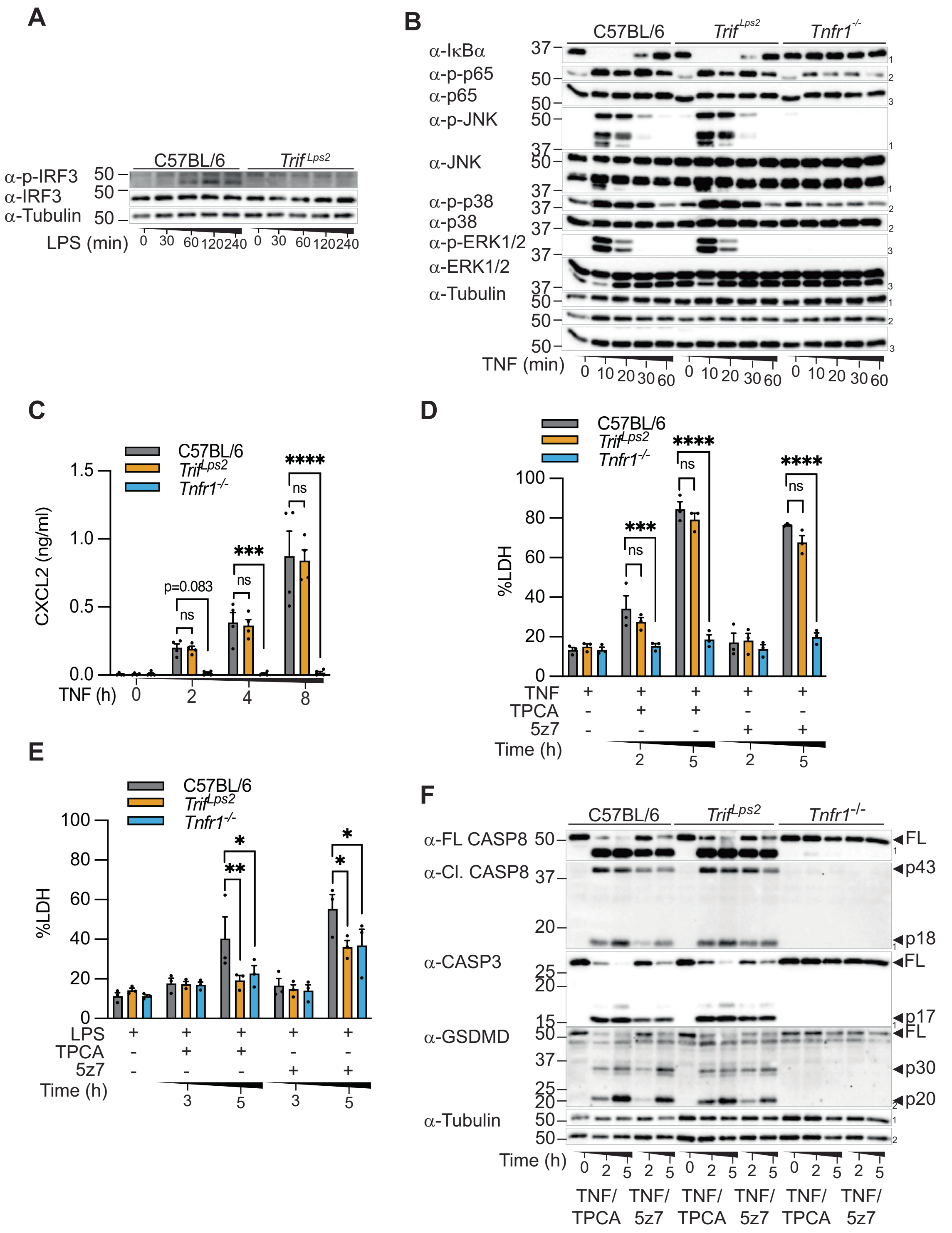

Figure EV2. WT and *Trif*^{Lps2} BMDMs display comparable TNF-mediated inflammation and cell death.

BMDMs were treated with (**A**) LPS (100 ng/ml) or (**B, C**) TNF (100 ng/ml) for the indicated time points. BMDMs were co-stimulated with (**D, F**) TNF (100 ng/ml) and TPCA (IKK inhibitor; 2.5 μM) or 5z7 (TAK1 inhibitor; 125 nM) or (**E**) LPS (100 ng/ml) and TPCA (2.5 μM) or 5z7 (125 nM) for the indicated time points. (**A, B**) Cell lysates or (**F**) mixed supernatant and lysates were examined by immunoblotting. (**C**) Cytokine secretion and (**D, E**) LDH release were quantified. (**C**) Pooled data are mean ± SEM of four independent experiments ($P = 0.0003$ for 4 h C57BL/6 vs *Tnfr1*$^{-/-}$ and $P \leq 0.0001$ for 8 h C57BL/6 vs *Tnfr1*$^{-/-}$). (**D**) Pooled data are mean ± SEM of three independent experiments ($P = 0.0003$ for 2 h TNF/TPCA treatment in C57BL/6 vs *Tnfr1*$^{-/-}$ and $P \leq 0.0001$ for 5 h TNF/TPCA or TNF/5z7 treatment in C57BL/6 vs *Tnfr1*$^{-/-}$). (**E**) Pooled data are mean ± SEM of three independent experiments (for 5 h LPS/TPCA treatment, $P = 0.0056$ in C57BL/6 vs *Trif*^{Lps2} and $P = 0.0208$ in C57BL/6 vs *Tnfr1*$^{-/-}$; for 5 h LPS/5z7 treatment, $P = 0.0112$ in C57BL/6 vs *Trif*^{Lps2} and $P = 0.0152$ in C57BL/6 vs *Tnfr1*$^{-/-}$). All P values were calculated with two-way ANOVA test. Data are considered significant when $P \leq 0.05$, with $^{*}P \leq 0.05$, $^{**}P \leq 0.01$, $^{***}P \leq 0.001$ or $^{****}P \leq 0.0001$. $^{\#}$non-specific band. Source data are available online for this figure.

© The Author(s)

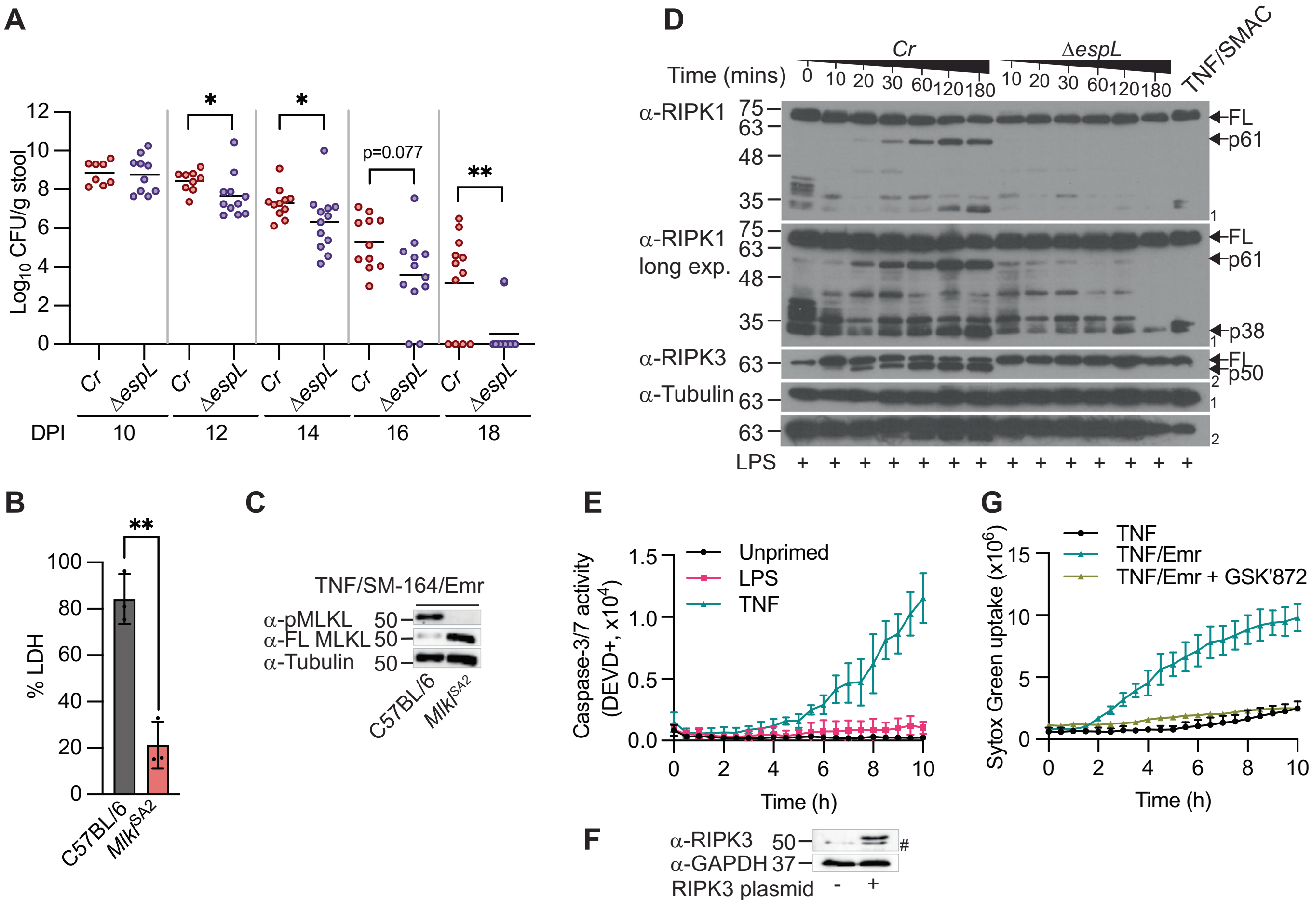

Figure EV3. EspL is essential for in vivo colonisation and for subverting macrophage necroptosis.

(**A**) C57BL/6 mice were infected with *C. rodentium* (*Cr*) or Δ*espL* and bacterial load in stool was enumerated. Each data point represents $\log_{10}$ colony forming unit (CFU) per gram of stool of an individual mouse and means are indicated. Eight to twelve mice were included per group ($P = 0.0251$ for day 12, $P = 0.0225$ for day 14 and $P = 0.0032$ for day 18). (**B, C**) BMDMs were primed with TNF (100 ng/ml) for 3 h and stimulated with emricasan (10 µM) in the last 30 min of priming followed by 5 h of stimulation with SM-164 (5 µM). (**D**) BMDMs were primed with LPS (100 ng/ml) for 3 h before infected with log-phase *C. rodentium* (*Cr*) and Δ*espL* for the indicated time points. (**E**) CMT-93 were stimulated with LPS (100 ng/ml) or TNF (100 ng/ml) over time. (**F**) RIPK3 expression in CMT-93 parental cells and CMT-93 stably overexpressing RIPK3 were confirmed by immunoblotting. (**G**) RIPK3-expressing CMT-93 cells were co-treated with TNF (100 ng/ml) and emricasan (10 µM). (**C, D**) Mixed supernatant and lysates or (**F**) cell lysates were examined by immunoblotting. (**D**) BMDMs co-stimulated with TNF (100 ng/ml)/ SMAC mimetic (1 µM) as positive control showing caspase-8 active cleavage of RIPK1. (**B**) LDH release were quantified. (**E**) Caspase-3/7 activity (DEVD-positive) and (**G**) SYTOX green uptake were quantified using IncuCyte. (**B**) Pooled data are mean ± SEM of three independent experiments ($P = 0.0018$). Data are mean ± SD of technical (**E**) triplicates or (**G**) duplicates representative of three independent experiments. *P* values in (**A**) and (**B**) were calculated with Mann–Whitney *t*-test and unpaired *t*-test respectively. Data are considered significant when $P \leq 0.05$, with $^*P \leq 0.05$ or $^{**}P \leq 0.01$. Source data are available online for this figure.

 © The Author(s)

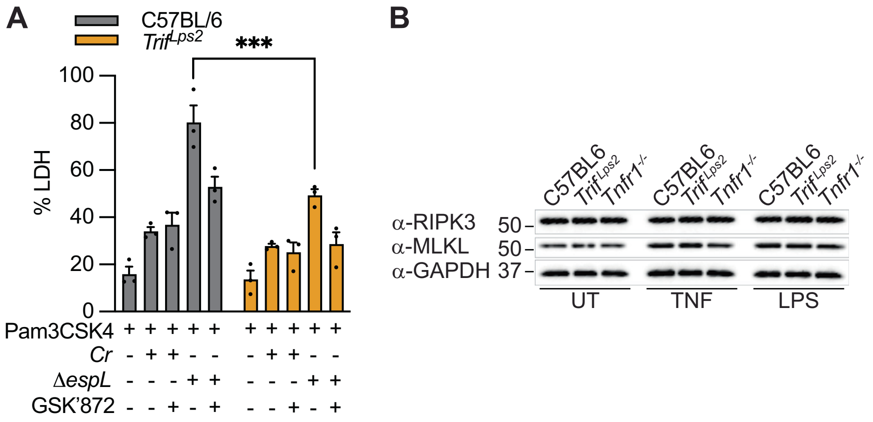

Figure EV4. ***Trif*** ^{Lps2} **mutation protects Pam3CSK4-primed macrophages from Δ*espL*-induced necroptosis.**

(**A**) BMDMs were primed with Pam3CSK4 (1 µg/ml) for 3 h before infected with log-phase *C. rodentium* (*Cr*) or Δ*espL* for 5 h. LDH release was quantified. Where indicated, cells were treated with GSK'872 (5 µM) for 30 min before infection. (**B**) BMDMs were stimulated with TNF (100 ng/ml) or LPS (100 ng/ml) for 3 h and cell extracts were analysed by immunoblotting. (**A**) Pooled data are mean ± SEM of three independent experiments ($P = 0.0002$). P value was calculated with two-way ANOVA test. Data are considered significant when $P \leq 0.05$, with ***$P \leq 0.001$. Source data are available online for this figure.

© The Author(s)

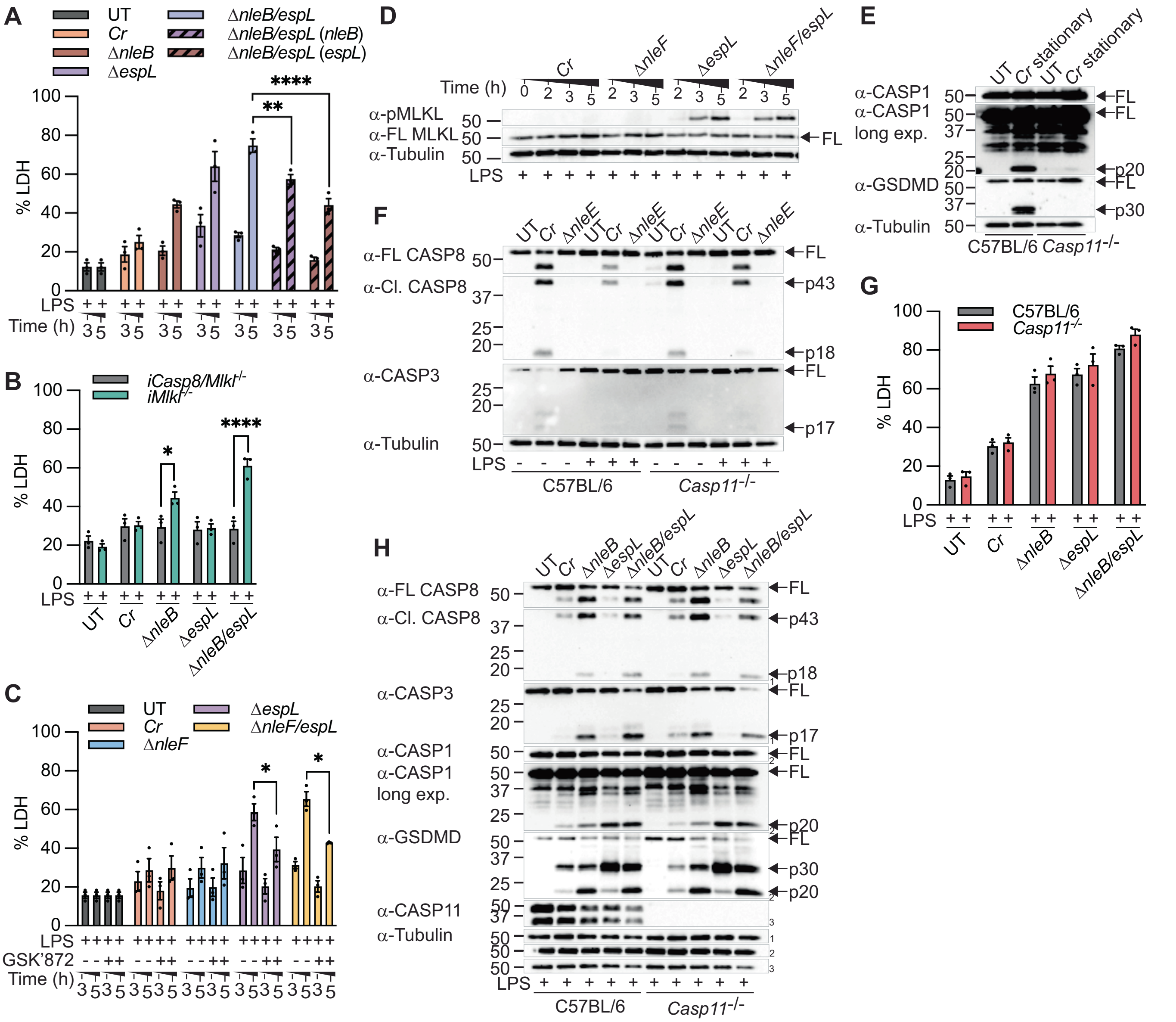

Figure EV5. Log-phase *C. rodentium* activates caspase-8 but not caspase-11 in macrophages.

(**A–H**) BMDMs were unprimed or primed with LPS (100 ng/ml) for 3 h before infected with (**A–D**, **F–H**) log- or (**E**) stationary-phase *C. rodentium* and the various mutants for (**A**, **C**, **D**) the indicated time points, (**B**, **F–H**) 5 h or (**E**) 16 h. (**A–C**, **G**) LDH release was quantified. (**D–F**, **H**) Mixed supernatant and lysates were examined by immunoblotting. (**A**) Pooled data are mean ± SEM of three independent experiments ($P = 0.0080$ for Δ*nleB/espL* vs Δ*nleB/espL* (*nleB*) and $P \leq 0.0001$ for Δ*nleB/espL* vs Δ*nleB/espL* (*espL*)). (**B**) Pooled data are mean ± SEM of three independent experiments ($P = 0.0153$ for Δ*nleB* and $P \leq 0.0001$ for Δ*nleB/espL*). (**C**) Pooled data are mean ± SEM of three independent experiments ($P = 0.0494$ for Δ*espL* treated with GSK'872 and $P = 0.0166$ for Δ*nleF/espL* treated with GSK'872). (**G**) Pooled data are mean ± SEM of three independent experiments. All *P* values were calculated with two-way ANOVA test. Data are considered significant when $P \leq 0.05$, with $^{*}P \leq 0.05$, $^{**}P \leq 0.01$, or $^{****}P \leq 0.0001$. Source data are available online for this figure.

 © The Author(s)
